# Identification of disulfidptosis-related subtypes, characterization of tumor microenvironment infiltration, and development of a prognosis model in breast cancer

**DOI:** 10.3389/fimmu.2023.1198826

**Published:** 2023-11-15

**Authors:** Jiahui Liang, Xin Wang, Jing Yang, Peng Sun, Jingjing Sun, Shengrong Cheng, Jincheng Liu, Zhiyao Ren, Min Ren

**Affiliations:** ^1^ Department of Breast Surgery, Department of General Surgery, The First Affiliated Hospital of Anhui Medical University, Hefei, Anhui, China; ^2^ Faculty of Medicine and Health Sciences, Ghent University, Ghent, Belgium; ^3^ Department of Respiratory and Critical Care Medicine, The First Affiliated Hospital of Anhui Medical University, Hefei, China; ^4^ Department of Plastic Surgery, The First Affiliated Hospital of Anhui Medical University, Hefei, China

**Keywords:** breast cancer, disulfidptosis, tumor microenvironments, prognosis, immunotherapy

## Abstract

**Introduction:**

Breast cancer (BC) is now the most common type of cancer in women. Disulfidptosis is a new regulation of cell death (RCD). RCD dysregulation is causally linked to cancer. However, the comprehensive relationship between disulfidptosis and BC remains unknown. This study aimed to explore the predictive value of disulfidptosis-related genes (DRGs) in BC and their relationship with the TME.

**Methods:**

This study obtained 11 disulfidptosis genes (DGs) from previous research by Gan et al. RNA sequencing data of BC were downloaded from the Cancer Genome Atlas (TCGA) and Gene Expression Omnibus database (GEO) databases. First, we examined the effect of DG gene mutations and copy number changes on the overall survival of breast cancer samples. We then used the expression profile data of 11 DGs and survival data for consensus clustering, and BC patients were divided into two clusters. Survival analysis, gene set variation analysis (GSVA) and ss GSEA were used to compare the differences between them. Subsequently, DRGs were identified between the clusters used to perform Cox regression and least absolute shrinkage and selection operator regression (LASSO) analyses to construct a prognosis model. Finally, the immune cell infiltration pattern, immunotherapy response, and drug sensitivity of the two subtypes were analyzed. CCK-8 and a colony assay obtained by knocking down genes and gene sequencing were used to validate the model.

**Result:**

Two DG clusters were identified based on the expression of 11DGs. Then, 225 DRGs were identified between them. RS, composed of six genes, showed a significant relationship with survival, immune cell infiltration, clinical characteristics, immune checkpoints, immunotherapy response, and drug sensitivity. Low-RS shows a better prognosis and higher immunotherapy response than high-RS. A nomogram with perfect stability constructed using signature and clinical characteristics can predict the survival of each patient. CCK-8 and colony assay obtained by knocking down genes have demonstrated that the knockdown of high-risk genes in the RS model significantly inhibited cell proliferation.

**Discussion:**

This study elucidates the potential relationship between disulfidptosis-related genes and breast cancer and provides new guidance for treating breast cancer.

## 1 Introduction

In recent years, the incidence of breast cancer in women has continued to grow at a rate of 0.5% per year. By 2022, breast cancer has surpassed lung cancer and has become the world’s highest incidence of tumors. According to the latest estimates, there will be 297,790 new breast cancer cases and 43,170 deaths among women in the United States in 2023, making it the leading cause of cancer death among women aged 20-49 (1). Many treatment options for BC have been developed, including surgery, chemotherapy, endocrine therapy, immune antibody (trastuzumab) therapy, and radiotherapy, based on disease stage and pathological characteristics (2, 3). Although the prognosis of breast cancer has improved significantly in the past few decades, even if patients pass standard diagnosis and treatment, 20–30% of BC patients still have distant metastasis, accounting for about 90% of all breast cancer deaths (2).

As a highly complex and heterogeneous disease with different molecular spectrums, breast cancer limits the broad application of classification and standard treatment to some extent. Furthermore, it is difficult to predict the prognosis of BC (2, 4). Therefore, we urgently need to explore the characteristics of the high-risk population of breast cancer patients to obtain the key markers of prognosis and to find potential therapeutic targets to designate individualized treatment to improve the prognosis of patients.

SLC7A11-mediated cystine reduction to cysteine highly depends on the reduced nicotinamide adenine dinucleotide phosphate (NADPH) generated by the glucose–pentose phosphate pathway. Under glucose starvation, the NADPH in SLC7A11-high-expression cells is consumed in large quantities, and the abnormal accumulation of disulfides, such as those in cystine, induces disulfide stress. This causes actin filaments to aggregate and contract rapidly, peeling off the plasma membrane before apparent cell death, called disulfidptosis. During this process, SLC7A11 and SLC3A2, which encode the SLC7A11 chaperone protein, mediate the reduction of ingested cystine to cysteine. Next, the WAVE regulatory complex (WRC) can activate seven subunits of actin-related protein 2 and 3 (Arp2/3) complexes to promote actin polymerization and plate pseudopod formation. This produces a branched cortical actin network under the plasma membrane, thereby stripping actin filaments from the plasma membrane. Nck-related protein 1 encoded by NCKAP1 is a WRC component. Its deletion will reduce the protein levels of other components in the WRC, including WAVE-2, CYFIP 1, Abi 2, and HSPC 300, and inhibit disulfidptosis. Similarly, knocking out the other four components of the WRC will also repress disulfidptosis. Finally, Rac can activate WRC to promote plate pseudopod formation and disulfidptosis. Knockout of the RPN1 gene, which encodes an N-oligosaccharide transferase in the endoplasmic reticulum, will offer UMRC6 cells stronger resistance to disulfidptosis.

However, DRGs have not been demonstrated in the survival prognosis, tumor immune microenvironment, TMB, immunity, and clinical treatment of BC patients. We still lack direct evidence of the predictive ability of DRGs for BC prognosis and immunotherapy. This study aimed to construct a risk score (RS) model based on DRGs to predict the prognosis of BC patients. In this study, TCGA and GEO databases were used to obtain DRGs by analyzing the difference in consensus clustering of DGs. The RS model of DRGs was built using the LASSO-Cox method. Further, we verified that this feature could be used as a reliable, independent predictor of prognosis and immune-sensitive response and could predict the prognosis of BC patients. Through this model, patients were divided into high-risk and low-risk groups. The differences in survival outcomes, tumor immune microenvironment, and immunotherapy response of BC patients were analyzed. According to the differences between groups, drug sensitivity studies were conducted to find sensitive drugs in different populations, and individualized intervention was implemented to improve the prognosis of BC patients.

## 2 Methods

### 2.1 Data collection and processing

**Figure 1:**
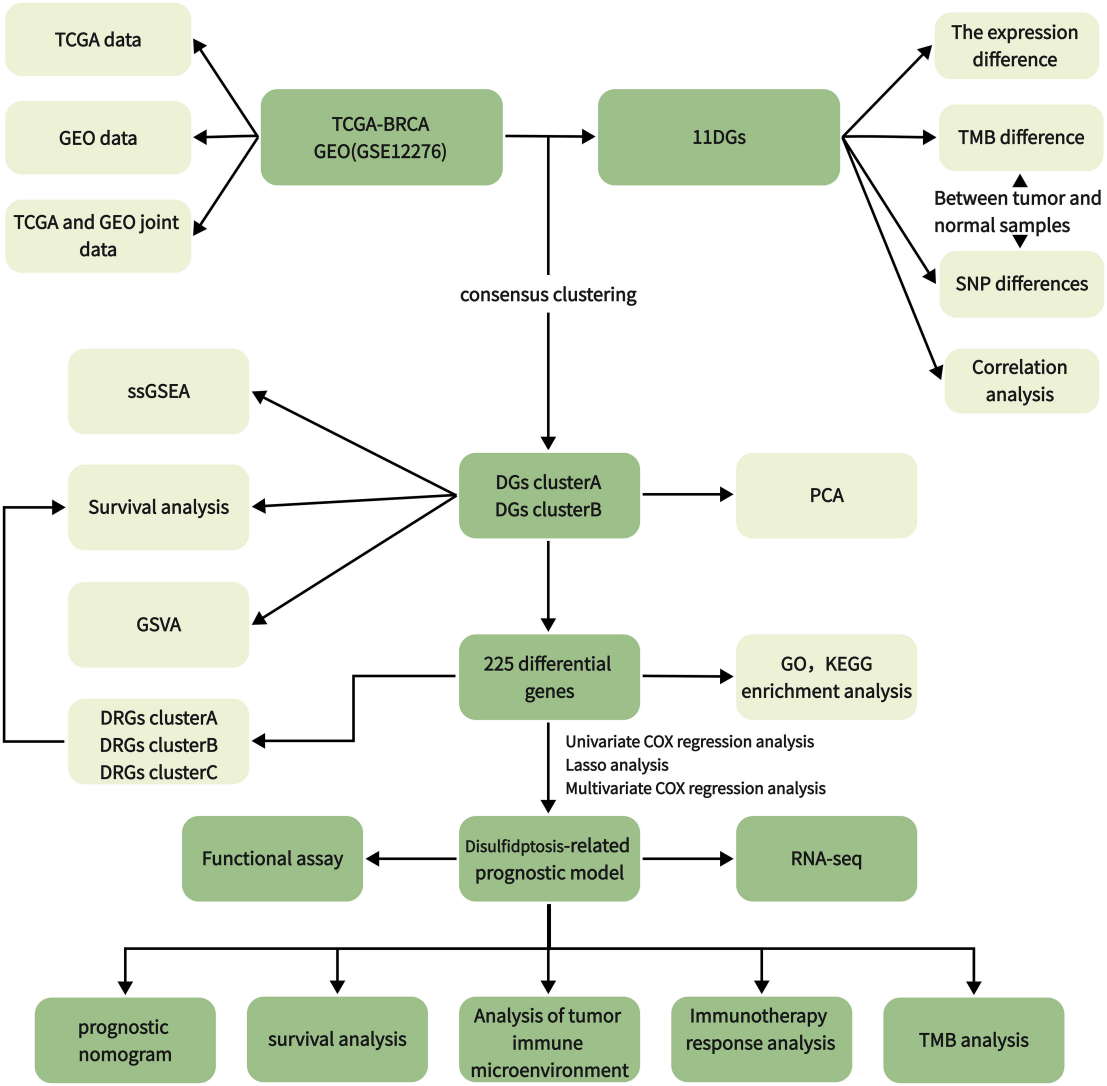
Workflow diagram.

We downloaded and organized 1,180 samples (1,081 tumor and 99 normal samples) from the TCGA database using the TCGAbiolinks and SummaryExperiment package in R v.4.2.2 (**Supplementary Table 1**). We searched the GEO database with “breast cancer” and “survival information” as keywords and screened according to the following criteria: first, all samples were from humans; second, all data sets included matched cancer tissue samples and clinical information; and third, the data set contained at least 200 samples. We downloaded the GSE12276 and GSE20685 gene expression profile, GPL570 platform annotation information, and clinical information using GEOquery, the data transmission tool of the GDC application. Gene expression profiles were standardized using the scaling method provided in the limma R package (5). TCGA and GEO gene expression profile information and clinical data are publicly available and open access. Therefore, no ethical issues were involved. Clinicopathologic features include gender, age, T (tumor) stage, N (lymph node metastasis) status, M (distant metastasis) status, tumor grade, survival status, and survival time. The tumor mutation data were derived from TCGA [GDC (cancer.gov)], and the gene copy number was downloaded from the Xena database (UCSC Xena). We obtained 11 disulfidptosis genes (DGs) from a previous study (6). The workflow of the current study is shown in **Figure 1**.

### 2.2 Survival, TMB, and CNV difference analysis between DGs

We used the Limma package using wilcox.test to test the difference in DG expression between tumor and normal samples (p < 0.05). R package survival and survminer were used for the survival analysis of DGs. DGs were divided into high- and low-expression groups according to gene expression. The survival differences between the two groups were compared (p < 0.05). Tumor mutation burden (TMB) refers to the number of somatic nonsynonymous mutations or all mutations per megabase in the gene region detected by whole exome sequencing or targeted sequencing in a tumor sample (7). Mutation data were downloaded from the Cancer Genome Atlas Breast Cancer (TCGA-BRCA) collection using the GDCquery package with 988 data downloads. According to the data.category = Simple Nucleotide Variation, data.type = Masked Somatic Mutation. The mutation data were analyzed with the R package maftools (8). The mutation probability of DGs in each tumor sample was calculated and analyzed. CNV (Copy Number Variation) is the increase or decrease in the copy number of genomic fragments caused by genome rearrangement. Xena downloaded and collated copy number variation data and used R-package RCircos to visualize the CNV of DGs.

### 2.3 Establishment of the disulfidptosis gene cluster

We used the R software package “ConsensusCluster Plus” for consensus clustering analysis and identified clusters of BC patients based on DGs. We set the cluster count (k) between two and nine and selected the optimal k value based on the sum of squared errors (SSE) inflection point. The stability of the DG group was verified by the PCA algorithm. Additionally, Kaplan-Meier survival analysis evaluated the OS of different DG clusters, and the heat map showed the degree of difference in DGs between the two groups.

### 2.4 TME infiltration and functional enrichment analysis of different clusters

Gene set variation analysis (GSVA) is a particular gene set enrichment method. This method works on single samples and enables pathway-centric analyses of molecular data by performing a conceptually simple but powerful change in the functional unit of analysis from genes to gene sets. We used the GSVA and GSEABase packages to evaluate the pathways enriched in groups A and B. We attempted to explain the reasons for the difference in survival between the groups from the bioinformatics perspective (9). Single sample gene set enrichment analysis (ssGSEA) was used to quantitatively analyze the immune infiltration of the overall sample and to observe the difference in component immune infiltration (10). We used the “limma” R package to analyze the DRGs between the two disulfidptosis clusters (p < 0.05, |logFC| = 0.585). Finally, we used the “ggplot2” package. The gene ontology (GO) analysis of DRGs was performed, and the histogram, bubble diagram, and circle diagram were drawn to explore its function and biological process. The Kyoto Encyclopedia of Genes and Genomes (KEGG) pathway enrichment analysis, histogram, and bubble diagram were drawn to explore its function and biological processes.

### 2.5 Identification of DRGs and construction of the prognostic signature

Before constructing the risk prediction model, we screened the features. First, a univariate Cox model was used to explore the relationship between 225 DRGs and OS of patients. A total of 114 single-factor DRGs related to BC prognosis were obtained. The least absolute shrinkage and selection operator (LASSO) was used to avoid overfitting in the TCGA training cohort (11). The prognostic DRG model was established using multivariate Cox regression analysis and the step Akaike information standard (stepAIC) value. The RS for each patient was calculated by combining the expression of each gene (Ei), LASSO coefficients (Li), and RS = Σ*^n^_i_ Ei* * *Li*. The patients were divided into high-risk and low-risk groups according to the median RS. Finally, the Kaplan–Meier survival curve was used to analyze the difference in overall survival between the two groups.

The sensitivity and specificity of the prognostic indicators were evaluated using the receiver operating characteristic (ROC) curve and the area under the ROC curve (AUC). A bootstrap method based on 1,000 resamplings was used to obtain the test set (12, 13) to verify the effectiveness of the prediction model. The training set was a combined dataset established using TCGA and GEO based on common genes. In the test set, the AUC of the prognostic model was calculated using the R package “riskRegression.” Subsequently, the stability of the ROC model was tested in the separate TCGA and GEO datasets, and the TCGA and GEO joint datasets merged based on common genes. Analyze the relationship between RSs and clinical factors, verify the effectiveness of risk markers, and further compare the survival prediction ability of prognostic factors. The independence of the prognostic model was verified using univariate and multivariate Cox regression analyses by comparing the clinical characteristics of the patients. At the same time, the nomogram was constructed with the Cox regression coefficient of the package “rms,” and the calibration curve was drawn.

### 2.6 Establishment of DRG cluster

We used the “ConsensusCluster Plus” package for consistency clustering analysis and identified clusters of BC patients based on DRGs. We set the cluster count (k) between two and nine and selected the optimal k value based on the sum of the squared errors (SSE) inflection point. The stability of the DG group was verified using a PCA algorithm. Additionally, Kaplan–Meier survival analysis evaluated the OS of different DG clusters, and the heat map showed the difference in DGs between groups.

### 2.7 Different tumor immune microenvironment patterns with RS

We used the CIBERSORT algorithm to calculate the proportion of tumor-infiltrating immune cells (14). The difference in the proportion of tumor-infiltrating immune cells between the high- and low-risk groups was compared. The ESTIMATE algorithm was used to evaluate the differences in immune, stromal, and tumor purity scores between the high- and low-risk groups. The tumor mutation burden was assessed based on whether the sample was considered high or low risk. Additionally, we used the Maftools package to perform somatic mutation analysis on breast cancer patients to view and analyze somatic mutation data. We also studied the relationship between BC RSs and cancer stem cells.

### 2.8 The role of RS based on DRGs in predicting drug sensitivity and clinical immune efficacy

For a long time, the research and development of new drugs have been a hot spot in breast cancer treatment. We used the pRRophetic package to calculate the half inhibitory concentration (IC50) of commonly used drugs in breast cancer patients. We screened out potential drugs with sensitivity differences between the two groups according to the risk level of breast cancer patients (p < 0.001) and drew a box plot. We used ggplot2, ggpubr, limma, and reforme2 R software packages to analyze the statistical differences in the expression levels of 79 common ICI-related immunosuppressive molecules (15). Tumor Immune Dysfunction and Exclusion (TIDE) [Tumor Immune Dysfunction and Exclusion (TIDE) (harvard.edu)] is a simple method to predict the immune escape of patients based on the evaluation of the tumor microenvironment using gene expression profiles. Patients with high TIDE scores have a high chance of antitumor immune escape (16). We obtained information on the immune escape ability after submitting the transcriptome data of TCGA-BRCA patients to the website. TCGA is a quantitative scoring scheme developed by developers using machine learning: it is a better predictor of anti-cytotoxic T lymphocyte antigen 4 (CTLA-4) and anti-programmed cell death protein 1 (anti-PD-1) antibody responses. We downloaded BC immunophenotype score (IPS) from TCIA database (https://tcia.at/). In order to predict the sensitivity of immunotherapy, we compared the IPS of high and low risk groups in different immunotherapy decisions (17).

### 2.9 Cell lines, cell culture, cell transfection, and real-time quantitative PCR

The Tumor Cell Line Comprehensive Analysis Database (DepMap Portal) was utilized to screen for cell lines for further experimental validation (18). Breast cancer cell lines (MDA-MB-468) were obtained from Sichuan Huijixin Biotechnology Co., Ltd. The MDA-MB-468 cells were grown in Dulbecco’s Modified Eagle Medium (DMEM) culture medium, supplemented with 10% Fetal Bovine Serum (FBS) in a standard humidified incubator with 5% CO2 at 37°C. The TMEM45A and SHCBP1 specific short hairpin RNAs (shRNAs) were synthesized from Chengdu Youkangjianxing Biotechnology Co., Ltd.

The sequences of shRNAs are as the following: sh- TMEM45A -1: 5′- TGCTGTTGACAGTGAGCGCGGTTAAAGTATTTGAATTTAATAGTGAAGCCACAGATGTATTAAATTCAAATACTTTAACCATGCCTACTGCCTCGGA-3′; sh- TMEM45A -2: 5′- TGCTGTTGACAGTGAGCGCGGTGTACAAAGAGTATTCTGATAGTGAAGCCACAGATGTATCAGAATACTCTTTGTACACCATGCCTACTGCCTCGGA′. sh- SHCBP1 -1: 5′-TGCTGTTGACAGTGAGCGCCACATTGATTTTTCAATTGAATAGTGAAGCCACAGATGTATTCAATTGAAAAATCAATGTGATGCCTACTGCCTCGGA-3′; sh- SHCBP1 -2: 5′- TGCTGTTGACAGTGAGCGCCAGCCAAATGTTGATATTAAATAGTGAAGCCACAGATGTATTTAATATCAACATTTGGCTGATGCCTACTGCCTCGGA -3′.

The knockdown efficiency was evaluated using real-time quantitative PCR (RT-qPCR) after 48 h transfection. The primer sequences used in the experiment are as follows. For the TMEM45A gene, the primers include qpcr-TMEM45A-F (forward primer): TTGGATGCCCACACTATGA and qpcr-TMEM45A-R (reverse primer): TCCATGGTCAAGGAGTTACA. For the SHCBP1 gene, the primers consist of qpcr-SHCBP1-F (forward primer): CTGGAGTTACAGAAGGATGGTG and qpcr-SHCBP1-R (reverse primer): CCATAGAAGCCTGTGGAATGT. After the knockdown of TMEM45A and SHCBP1 in cell line MDA-MB-468, total mRNA from cells was extracted with TRIzol reagent (TaKaRa, Japan). Then, concentration and purity were evaluated by Nanodrop 2000 (Thermo Fisher, USA). After the RNA was reversely transcribed into cDNA with PrimeScript RT kit (TaKaRa, Japan) according to the instructions, SYBR Premix Ex Taq TM kit (TaKaRa, Japan) was applied for RT-qPCR, with β-actin as the endogenous control gene. The RT-qPCR amplification instrument (ABI StepOne Plus) was used to detect the SYBR Green fluorescence signal level after each amplification cycle. Data processing was performed using GraphPad Prism 10.0.0, and T-test was conducted to compare the experimental group to the control group. This supplementary material has been reflected in the preceding text.

### 2.10 Cell proliferation assay

For three days, proliferation assays were conducted daily on BC cells in 96-well plates using Cell Counting Kit 8 (CCK8) reagent (Beyotime, China). Incubation occurred at 37°C for 2 h, and the plate was analyzed with a microplate reader at 450 nm to measure absorbance.

### 2.11 Colony formation assays

Approximately 500 cells per well were seeded into a 6-well culture plate and incubated at 37°C for two weeks. After washing with PBS twice, cells were fixed with 4% paraformaldehyde for 15 min and then dyed with crystal violet. Each experiment was repeated three times. ImageJ was used for image analysis to convert images into cellular count data (19). The acquired counts were normalized by dividing them by the corresponding cell count in the control group, yielding percentage data. Data and image processing were performed using GraphPad Prism 10.0.0. The statistical analysis consisted of a t-test conducted on three replicate datasets, comparing the experimental and control groups.

### 2.12 RNA-seq

Cells were used for RNA sequencing after the knockdown of TMEM45A and SHCBP1 in cell line MDA-MB-468. Approximately 2 μg of total RNA was extracted from each specimen and pretreated with Epicentre Ribo-zeroTM rRNA Removal Kit. Then, the RNA expression profile library was constructed in line with the manufacturer’s protocol of NEBNext R Ultratdirectional RNA Library Prep Kit(NEB, USA). The steps are as follows: First, RNA was lysed into small fragments after being treated with NEBNext first strand synthesis reaction buffer at high-temperature treatment, and the first strand cDNA was synthesized using random hexmer primers and M-MULv reverse transcriptase. The second strand dsDNA was then obtained, and the fragment residues were converted into blunt ends by exonuclease or polymerase. Subsequently, the 3’end of each dsDNA fragment was adenylated and connected to the NEBNext adapter with a hairpin structure. After purification using the AMPure XP system (Beckman Coulter, Beverly, USA), 150–200 bp DNA fragments were obtained and sequenced using HiSeq 2500 (Illumina, CA, USA).

### 2.13 RNA-seq data processing and analysis

FastQC (http://www.bioinformatics.babraham.ac.uk/projects/fastqc/) was utilized to check the sequencing quality of all the sample data trimmed using the FASTX Toolkit. The sequencing reads against the human assembly GRCh37 were mapped using TopHat (v 2.0.9). We perform differential analysis using the gene expression matrix in counts format. We employed the R package edgeR to perform differential analysis between snSHCBP1 and NC, as well as snTMEM45A and NC, using predetermined criteria (fold change > 1, padj < 0.05). Following this analysis, we created volcano plots to visualize the differential expression of genes. We conducted an intersection analysis to identify genes that exhibited consistent differential expression in both replicates. Subsequently, we performed Gene Ontology (GO) and Kyoto Encyclopedia of Genes and Genomes (KEGG) enrichment analyses (p-value < 0.05) on the genes found in the intersection.

## 3 Results

### 3.1 11DGs in breast cancer: expression, genetic variants, and prognostic values

**Figure 2:**
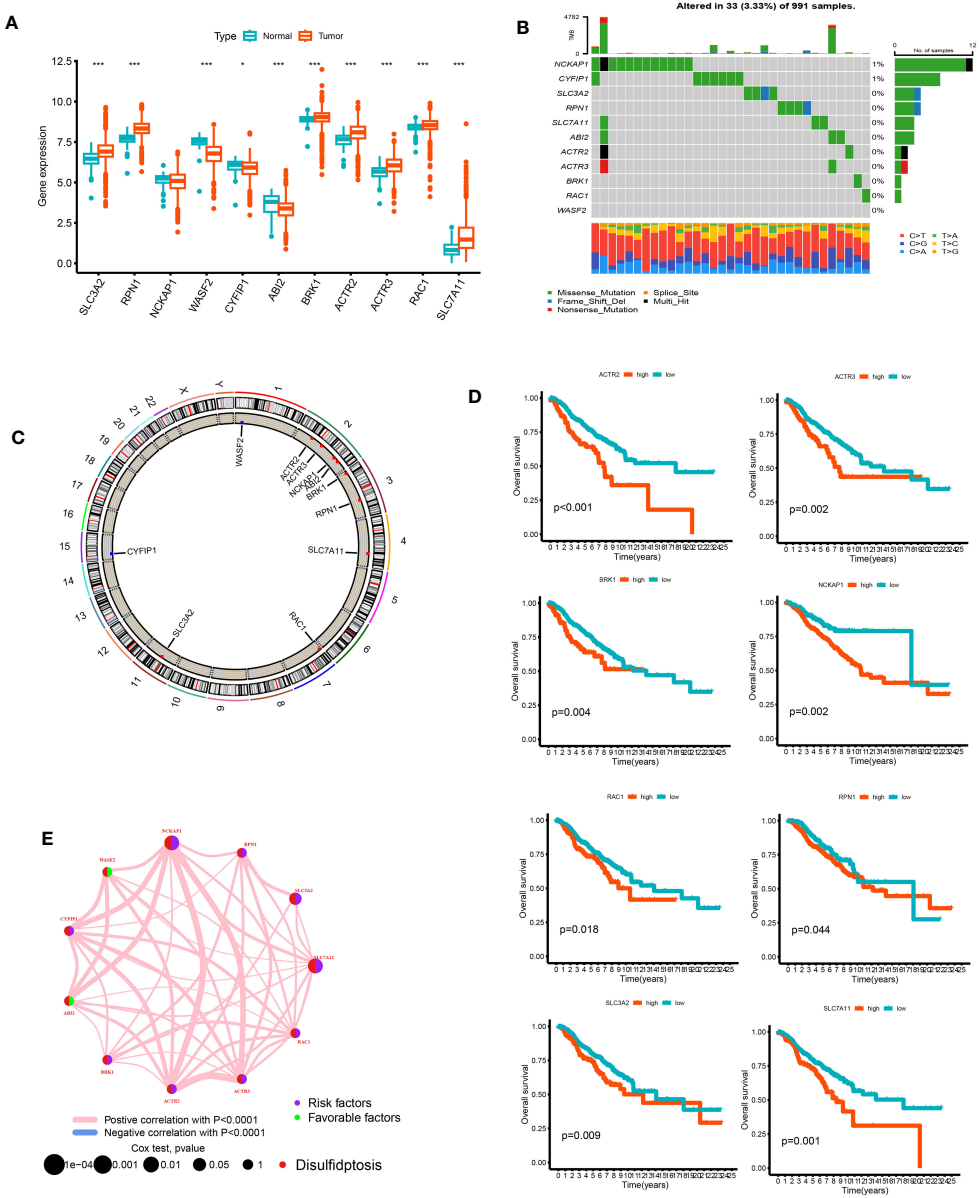
The analysis of 11 DGs' expression and association in the TCGA cohort. **(A)** The expression of the 11 DGs in BC tissues and healthy breast tissues (*p<0.05; ***p< 0.001). **(B)** Data on the frequency of DGs' mutations for 991 BC patients. **(C)** The sites of CNV variation in DGs on the 23 chromosomes. **(D)** The relationship between 8 DGs and overall s survival. **(E)** The interactions between DGs in BC (the red and blue strings denote positive and negative correlation, respectively; the intensity of the correlation is indicated by the color shades).

We analyzed the differences between 11 DGs in breast cancer and normal breast tissue samples. Except for NCKAP1, other genes significantly differed between tumor and normal groups (p < 0.05). The expression levels of SLC3A2, RPN1, BRK1, ACTR2, ACTR3, RAC1, SLC7A11, and WASF2 in the tumor samples were higher than those in the normal samples. On the other hand, the expression levels of WASF2, CYFIP1, and ABI2 in the normal samples were higher (**Figure 2A**). Of the 911 breast cancer samples of TCGA, 33 samples had DG mutations. The genes with the highest mutation frequencies were NCKAP1 and CYFIP1. Still, no base mutation was found in WASF2 (**Figure 2B**). The location and copy number changes of DGs on chromosomes are shown in **Figure 2C**. Among them, SLC3A2, BRK1, ABI2, ACTR2, NCKAP1, RAC1, RPN1, SLC7A11, and ACTR3 are upregulated in breast cancer, while CYFIP1 and WASF2 were downregulated. COX regression analysis and K-M survival analysis were performed on the expression of 11 DGs and the survival time of patients, and the tumor samples were divided into high- and low-risk groups according to the median value of a single gene. It can be concluded that SLC7A11, SLC3A2, RPN1, NCKAP1, BRK1, ACTR2, ACTR3, and RAC1 are single-factor genes that can predict the survival of patients (p < 0.05, **Supplementary Table 2**). The survival analysis was statistically significant, and the gene expression was negatively correlated with the survival time (**Figure 2D**). The 11 DGs positively regulated each other (**Figure 2E**).

### 3.2 Clusters of DGs identified in breast cancer

**Figure 3:**
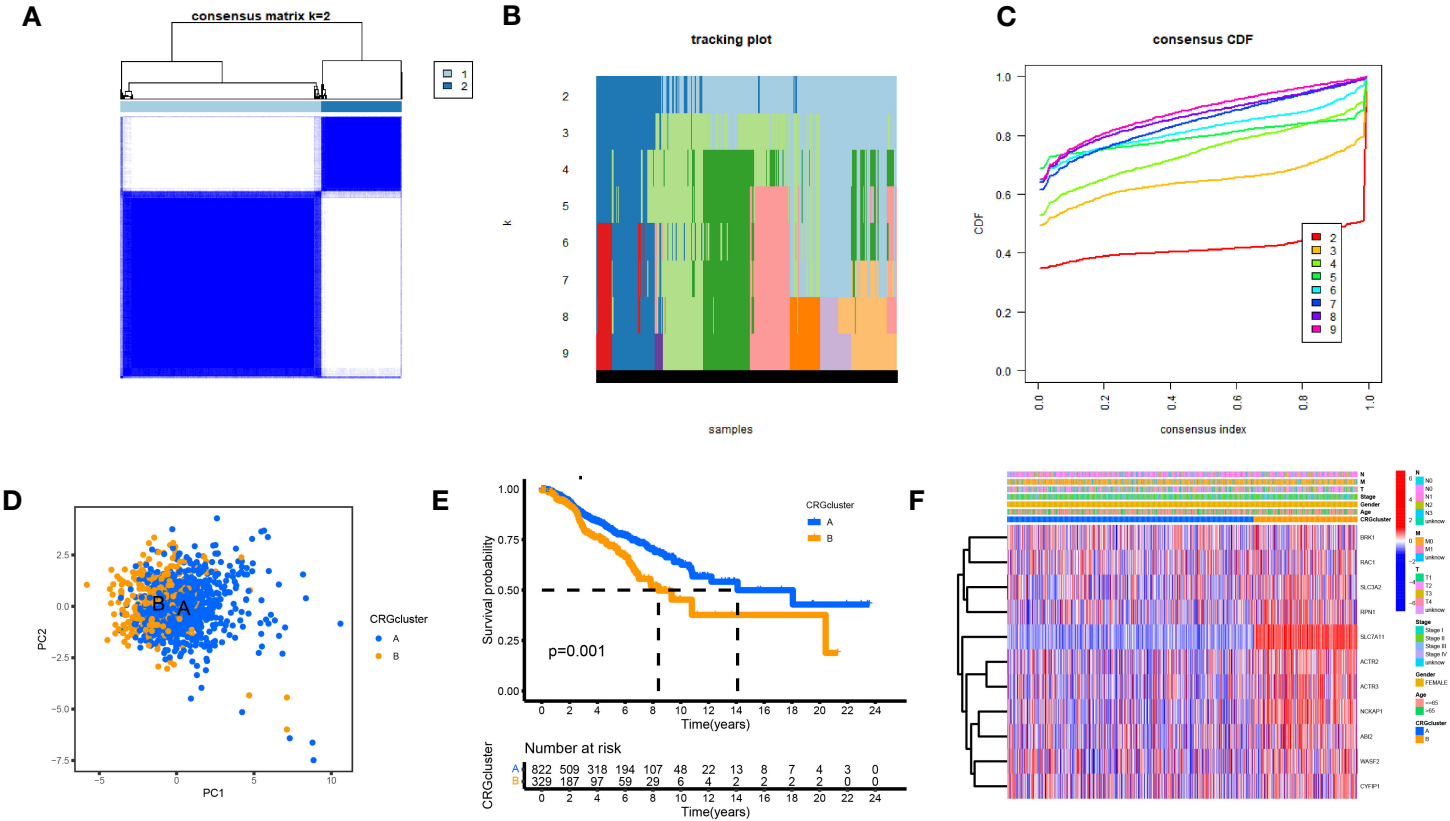
Biological and clinicopathological characteristics of DG subtypes. **(A)** The consensus matrix's heatmap of two clusters (κ = 2). **(B)** tracking plot. **(C)** Consensus CDF. **(D)** A considerable transcriptome divergence between the two subtypes is seen by PCA analysis. **(E)** Subtype-specific Kaplan-Meier OS curves. **(F)** DGs expression levels and clinicopathological traits vary across subtypes.

We performed a consistent cluster analysis of the expression levels of 11 DGs to explore their roles in the occurrence and development of breast cancer. Among the clustering variables, k = 2 had better clustering stability, the highest intra-group correlation, and the lowest inter-group correlation (**Figures 3A–C**). Therefore, we divided all tumor samples into clusters A (n = 339) and B (n = 850). PCA showed a significant interval between the two groups (**Figure 3D**). Survival analysis between groups A and B showed that the prognosis of group A was significantly better than that of group B, and the survival difference between the two groups was statistically significant (p < 0.05, **Figure 3E**). Additionally, the heat map between the two AB groups showed significant differences in the expression of 11 DGs between the two groups, especially between the SLC7A11 groups (**Figure 3F**).

### 3.3 TME infiltration and functional enrichment analysis of different clusters

**Figure 4:**
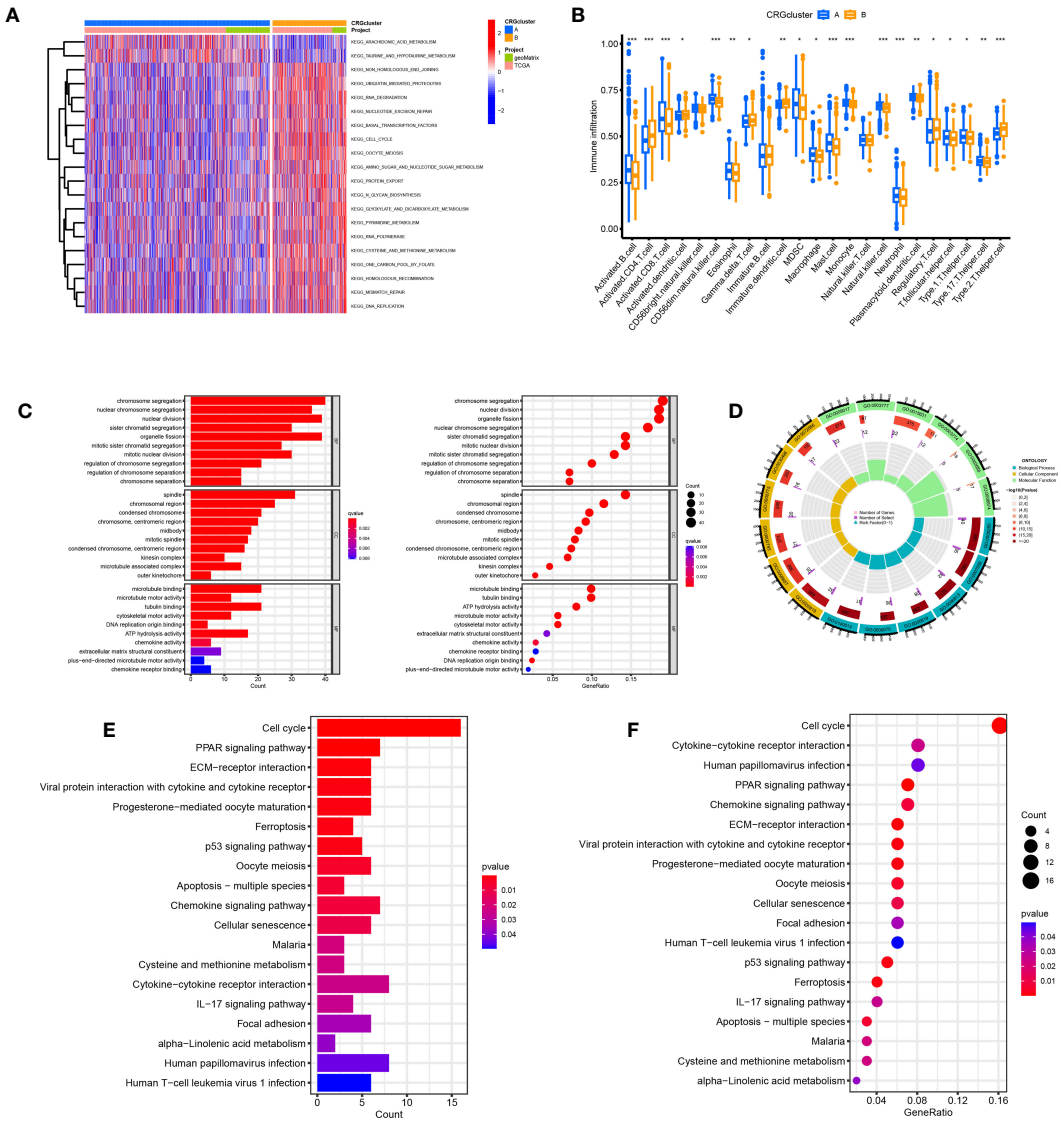
Disulfidptosis subtypes linked to TME invasion. **(A)** GSVA of two disulfidptosis subtype-related cellular pathways (Red means activated and blue means inhibited). **(B)** Correlations between immune cell infiltration levels in the two subtypes associated with disulfidptosis. **(C, D)** The GO function enrichment analyses. **(E, F)** The KEGG function enrichment analyses.

The samples in the A and B groups were analyzed using GSVA. From **Figure 4A**, it can be seen that group B was more active in arachidonic acid metabolism and taurine and hypotaurine metabolism pathways than group A. At the same time, group A was active in nonhomologous end connections, ubiquitin-mediated proteolysis, and other pathways. We performed a single sample gene set enrichment analysis (ssGSEA) on groups A and B. From **Figure 4B**, we can see Activated.B.cell, Activated.CD8.T.cell, CD56dim.natural.killer.cell, Eosinophil, MDSC, Macrophage, Mast.cell, Monocyte. Natural.killer.cell, Neutrophil, Plasmacytoid.dendritic.cell, T.follicular.helper.cell, Type.1.T.helper.cell, and Type.17.T.helper.cell were highly expressed in Cluster A and Activated.CD4.T.cell, Activated.dendritic.cell, Gamma.delta.T.cell, Immature.B.cell, Immature.dendritic.cell, Regulatory.T.cell, and Type.2.T.helper.cell were highly expressed in cluster B. We found 225 differential genes between groups A and B to explore the differences in biological processes between groups A and B according to the DG grouping.

We performed GO and KEGG enrichment analyses on these differential genes. As shown in **Figures 4C, D**, DRGs are mainly enriched in the nuclear division, chromosome segregation, and nuclear chromosome segregation in biological processes (BP). In addition, DRGs were primarily enriched in the spindle, chromosomal region, and condensed chromosome. Regarding molecular function, DRGs were mainly enriched in microtubule binding, tubulin binding, and ATP hydrolysis activity. We also performed a KEGG enrichment analysis to explore the differential genes in the pathway. The most enriched pathways were in the cell cycle. Cytokine−cytokine receptor interaction and human papillomavirus infection were the most significant enrichments in the cell cycle, PPAR signaling pathway, and ECM−receptor interaction (**Figures 4E, F**).

### 3.4 Clusters of DRGs identified in breast cancer

**Figure 5:**
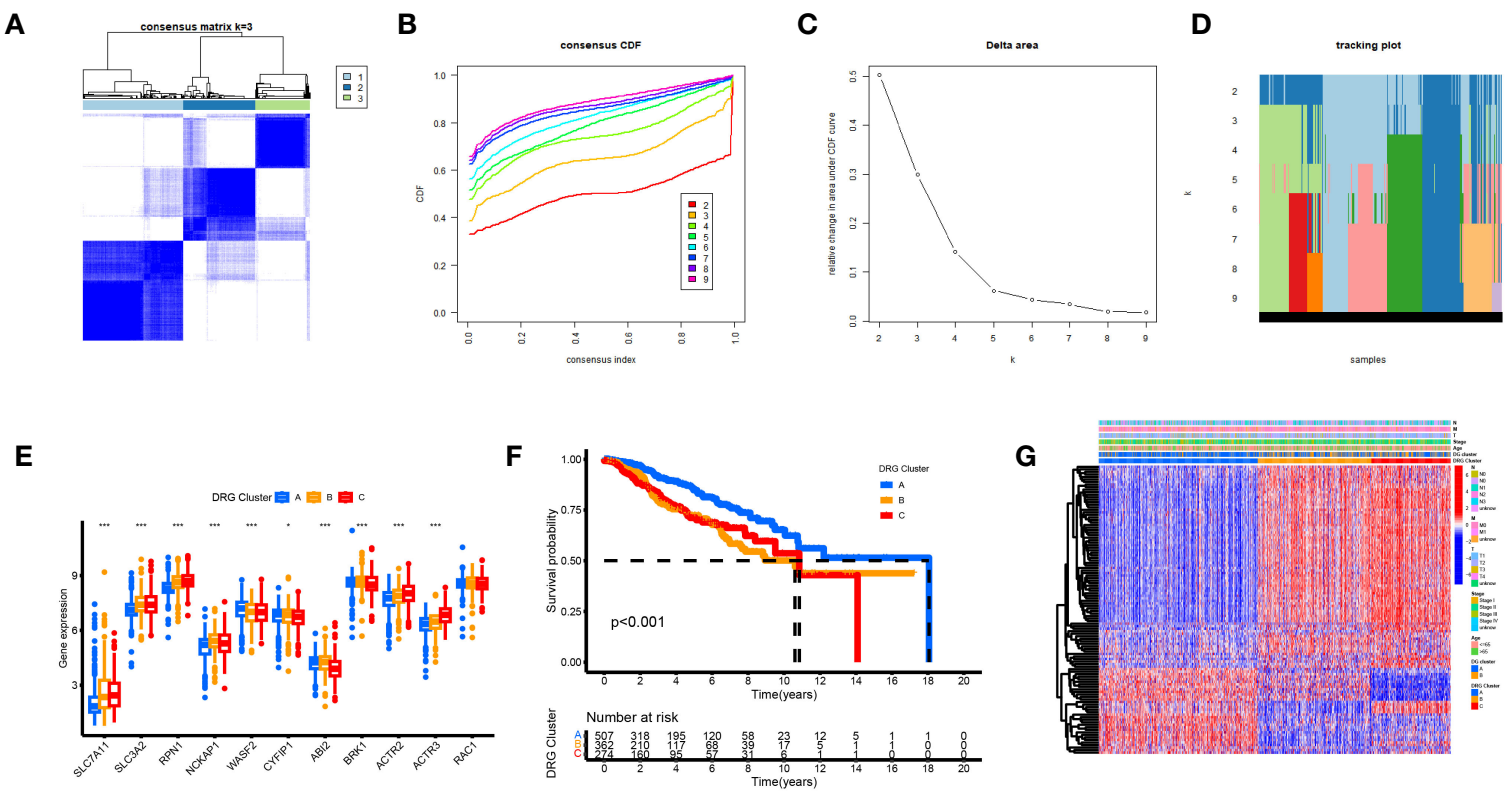
Biological and clinicopathological characteristics of DRG subtypes. **(A)** The consensus matrix's heatmap of two clusters (κ = 3). **(B)** Consensus CDF **(C)** Delta area. **(D)** tracking plot Consensus CDF. **(E)** DRGs expression levels and clinicopathological traits vary across subtypes. **(F)** Subtype-specific Kaplan-Meier OS curves. **(G)** DRGs expression levels and clinicopathological traits vary across subtypes.

Based on a consensus cluster analysis of 225 DRGs, the relationship between disulfidptosis and BRCA subtypes was explored. K = 3 is the appropriate choice for the most stable aggregation (**Figures 5A–D**). Therefore, we divided all tumor samples into three subgroups: cluster A (n = 525), cluster B (n = 379), and cluster C (n = 285). 11DGs exhibit significant differences among clusters A, B, and C (**Figure 5E**). **Figure 5F** shows that the prognosis of group A is the best, followed by group B, and the prognosis of group C is relatively poor. From **Figure 5G**, it can be seen that there were significant differences in the expression levels of DRGs between different clusters. The samples of DRG cluster A were mostly in cluster A of 11 DGs, and the survival prognosis of these two clusters was significantly better than that of other groups.

### 3.5 Creating and confirming the predictive RS

**Figure 6:**
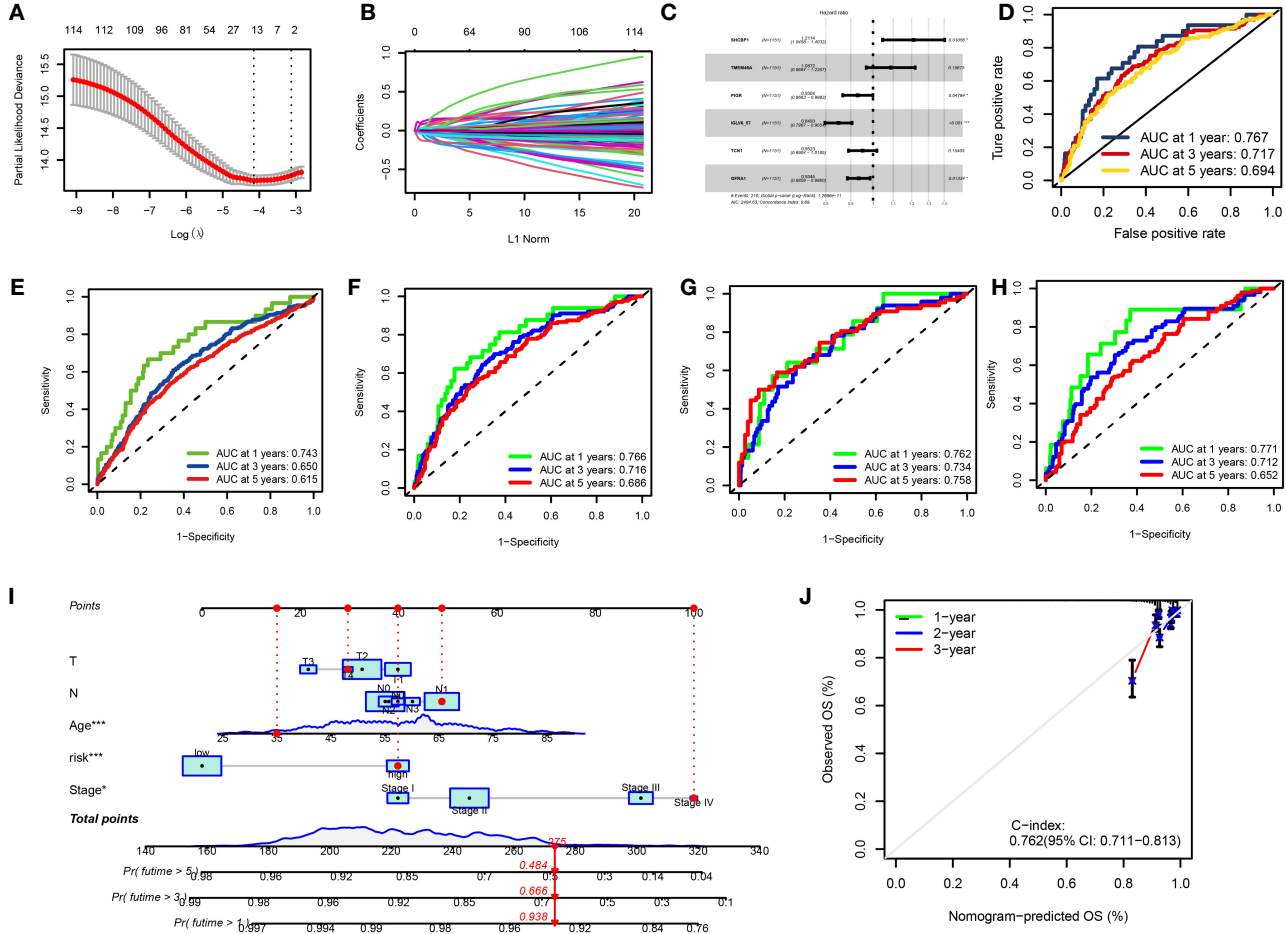
**(A, B)** LASSO variable trajectory plot for 1,000 cross validations **(A)** and LASSO coefficient profile **(B)**. **(C)** Forest Plot for Multifactorial Cox Regression Analysis. **(D)** ROC curves and AUCs for 1-, 3-, and 5-year survival rates. **(E)** ROC curves and AUCs for 1-, 3-, and 5-year survival rates in another independent GEO dataset. **(F)** ROC curves and AUCs for 1-, 3-, and 5-year survival rates in the TCGA and GEO merged datasets. **(G)** ROC curves and AUCs for 1-, 3-, and 5-year survival rates in TCGA datasets. **(H)** ROC curves and AUCs for 1-, 3-, and 5-year survival rates in GEO datasets. **(I)** The nomogram used to calculate the survival rates of 1-, 3-, and 5-years for patients with BC. **(J)** Calibration curve for nomograms.

We used univariate Cox regression analysis to extract 114 DRGs associated with BC prognosis in the preliminary screening (**Supplementary Table 3**). Using the LASSO regression algorithm, 14 BC-related genes were identified based on the minimum partial likelihood of the best λ value and deviation (**Figures 6A, B**, **Supplementary Table 4**). Multivariate Cox regression analysis was performed on these 14 genes, and a risk model consisting of six genes was obtained (**Figure 6C**). Among them, SHCBP and TMEM45A were molecules that improved prognosis. PIGR, IGLV6-57, TCN1, and GFRA1 were risk factors. The molecular formula of the model was as follows: RS = (0.1917 * SHCBP1 + 0.0836 * TMEM45A–0.0721 * PIGR–0.1633 * IGLV6–57–0.0489 * TCN1-0.0676 * GFRA1). In the bootstrap set, AUC at one year was 0.767, AUC at three years was 0.717, and AUC at five years was 0.694 (**Figure 6D**, **Supplementary Table 5**). Besides, we used the GEO database GSE20685 for external data verification. The AUC values of breast cancer patients predicted by our model were 0.743, 0.650, and 0.615 at one, three, and five years, respectively (**Figure 6E**). We validated it separately in the GEO dataset: AUC at one year was 0.762, AUC at three years was 0.734, AUC at five years was 0.758, AUC at one year was 0.771, AUC at three years was 0.712, and AUC at five years was 0.652 in TCGA. In the combined dataset of TCGA and GEO, the AUC at one year was 0.766. The AUC at three years was 0.716, and the AUC at five years was 0.686 (**Figures 6F–H**). BC patients were randomly selected for scoring, total point = point (T) + point (N) + point (RS) + point (stage) + point (age) below by combining RS and clinicopathological features, using the nomogram (a quantitative method), as shown in **Figure 6I**. The total score corresponds to the scale in the figure. It can predict 1-year, 3-year, and 5-year OS of BC patients. The calibration curve showed adequate consistency between the predicted values of the 1-year, 3-year, and 5-year OS nomograms and the actual observed values (**Figure 6J**).

### 3.6 Dividing the high- and low-risk groups and observing their distribution in clusters

**Figure 7:**
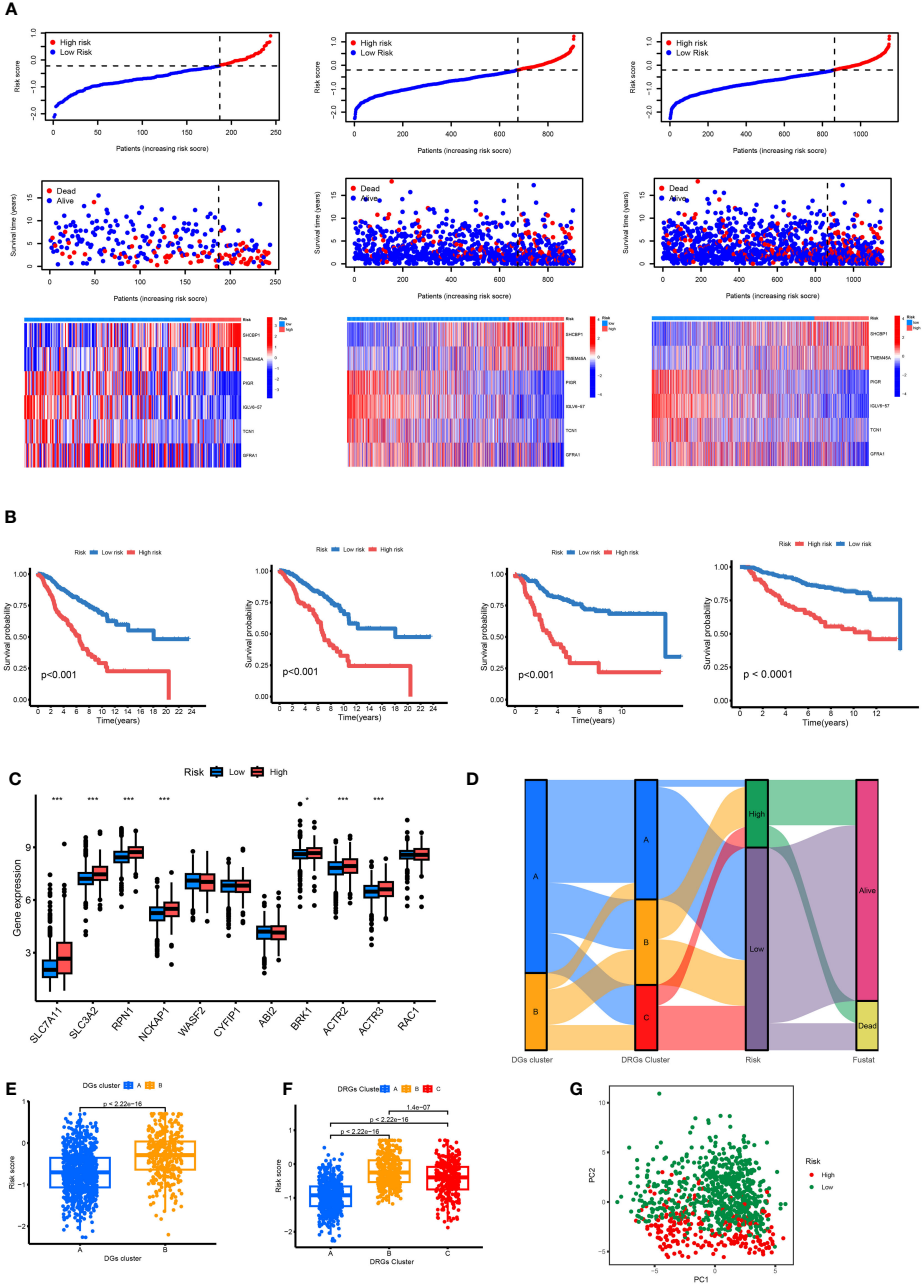
**(A)** Ranked dot, scatter plots and heat map of the model gene expressions in the TCGA and GEO merged datasets, TCGA datasets and GEO datasets. **(B)** Kaplan–Meier analyses of the OS between the TCGA and GEO merged datasets, TCGA datasets, GEO datasets and another independent GEO dataset. **(C)** RS score differences in eleven DGs. *p < 0.05, ***p < 0.001. **(D)** The subtype distributions among groups, risk scores and survival outcomes. **(E)** Variations in risk scores among DGs subtypes. **(F)** Variations in risk scores among CRGs subtypes. **(G)** Through PCA analysis, it can be seen that there are large transcriptome differences between high and low groups.

The patients in the TCGA, GEO, and total datasets were divided into high-risk and low-risk groups according to the median RS value. The differences between the groups were compared. **Figure 7A** shows that the genes (SLC7A11, SLC3A2, BRK1, ACTR2, ACTR3, RPN1, and NCKAP1) have the K-M survival analysis between the high- and low-risk groups showed that the survival differences between the high- and low-risk groups were statistically different (p < 0.001) in the TCGA dataset, GEO dataset, combined dataset and external validation of the GEO database. The prognosis of the significantly high-risk group was poor (**Figure 7B**). Seven of the 11 disulfide death genes showed significant differences in gene expression between high-risk and low-risk individuals (**Figure 7C**). **Figure 7D** illustrates the proportion of surviving and dying patients in two RS high and low groups, two DGs, and three DRG subtypes. **Figures 7E, F** show the RS distribution of two DG subtypes and three DRG subtypes, respectively. The PCA diagram shows that RS has an adequate grouping function (**Figure 7G**).

### 3.7 Different immune landscapes in the two risk groups

**Figure 8:**
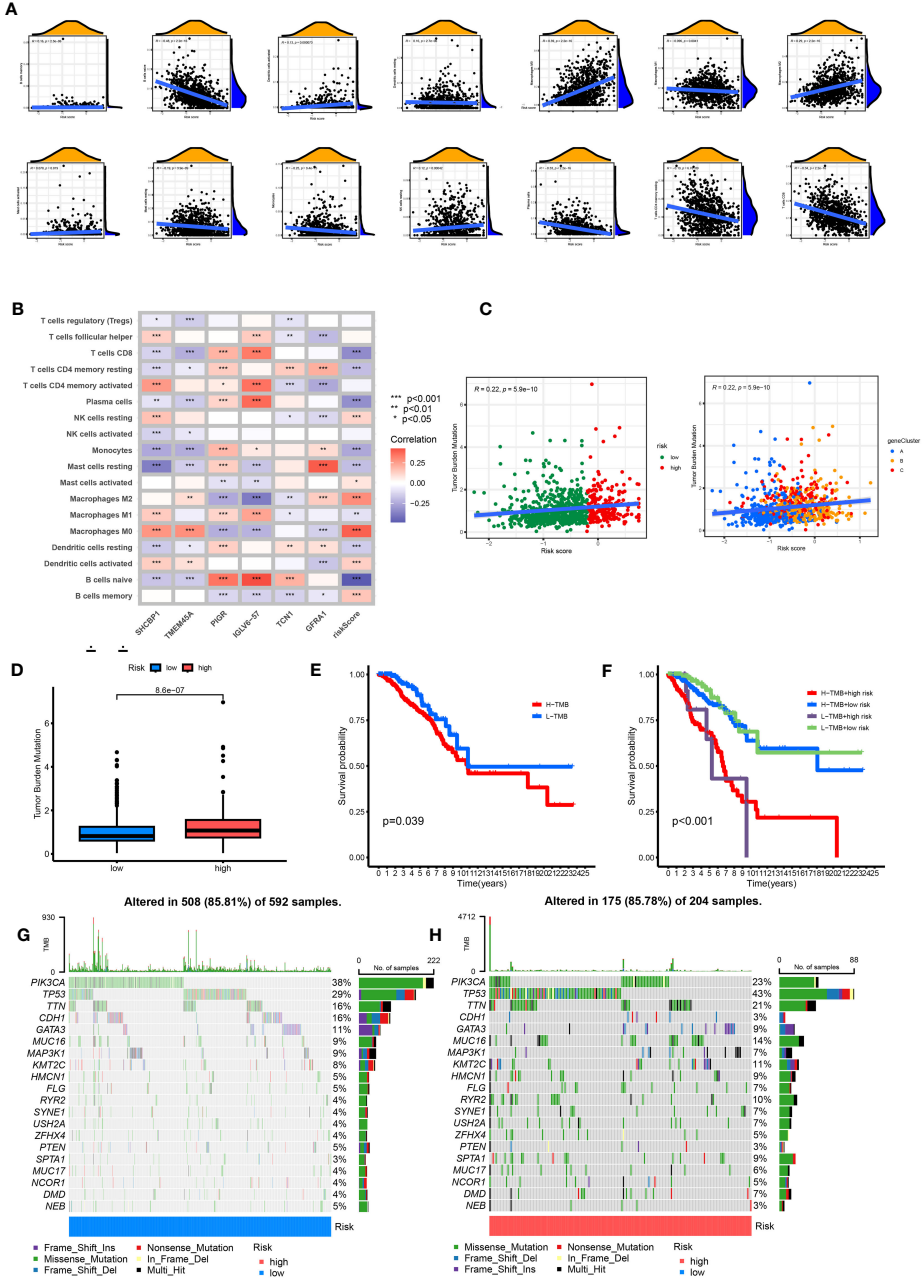
Comprehensive analysis of the risk scores in BC. **(A)** Correlations between immune cell types and risk score. **(B)** The six genes from the proposed model are correlated with the number of immune cells. **(C)** risk score and TMB spearman correlation analysis. **(D)** The differences in TMB between high- and low-risk groups. **(E)** Kaplan–Meier survival curves of BC patients between the H-TMB and L-TMB groups. **(F)** Kaplan–Meier survival curves of BC patients across H-TMB + high risk, H-TMB + low risk, L-TMB + high risk, and L-TMB + low risk. TMB, tumor mutational burden; H, high; L, low. **(G, H)** The somatic mutation features waterfall plot determined by high and low risk scores. One patient was represented by each column. The correct number represented each gene's frequency of mutation, and the upper barplot displayed TMB. The proportion of each variant type was displayed in the right barplot.

The CIBERSORT algorithm evaluated the relationship between RS and the relative number of immune cells. RS was positively correlated with the number of immune cells, such as Macrophages M0 and M2, Dendritic cells resting, Mast cells activated, B cells memory, NK cells resting, T cells CD4 memory resting, B cells naïve, Mast cells resting, and Monocytes. The expression of immune cells, such as dendritic cells activated, Plasma cells, Macrophages M1, and T cells CD8, was negatively correlated (p < 0.05). The stromal score, immune score, and ESTIMATE score of the high-risk group were higher. The difference between the groups was statistically significant (**Figure 8A**). Our study also examined the association between six genes and the number of immune cells (**Figure 8B**). According to our research, these six genes affect most immune cells. By observing the mutation frequency of each tumor sample and the mutation frequency of the gene, the TMB difference between the high- and low-risk groups was compared. From **Figures 8C, D**, it can be seen that the TMB of patients in the high-risk group is higher than that in the low-risk group, RS is positively correlated with TMB, and CRGsClusteA is significantly enriched in the low-risk area.

The TMB survival curve showed that patients with low TMB had a better prognosis (**Figure 8E**). Compared with other groups, BC patients with low risk and low TMB had the best prognoses (**Figure 8F**). The mutation rate of the low-risk group was 85.81% (**Figure 8G**). The mutation rate of the high-risk group was 85.78% (**Figure 8H**). The waterfall diagram shows that the mutation genes in the high- and low-risk groups are mainly PIK3 CA, TP53, TTN, CDH1, GATA3, MUC16, and MAP3K1, and the mutation rates of these genes are different in the high- and low-risk groups. PIK3CA had the highest mutation frequency in the low-risk samples, while TP53 had the highest in the high-risk groups. The mutation probability of TP53 in the high-risk group was 43%, while the mutation frequency of TP53 in the low-risk group was only 29%.

### 3.8 Drug sensitivity and different immunotherapy responses in the two risk groups

**Figure 9:**
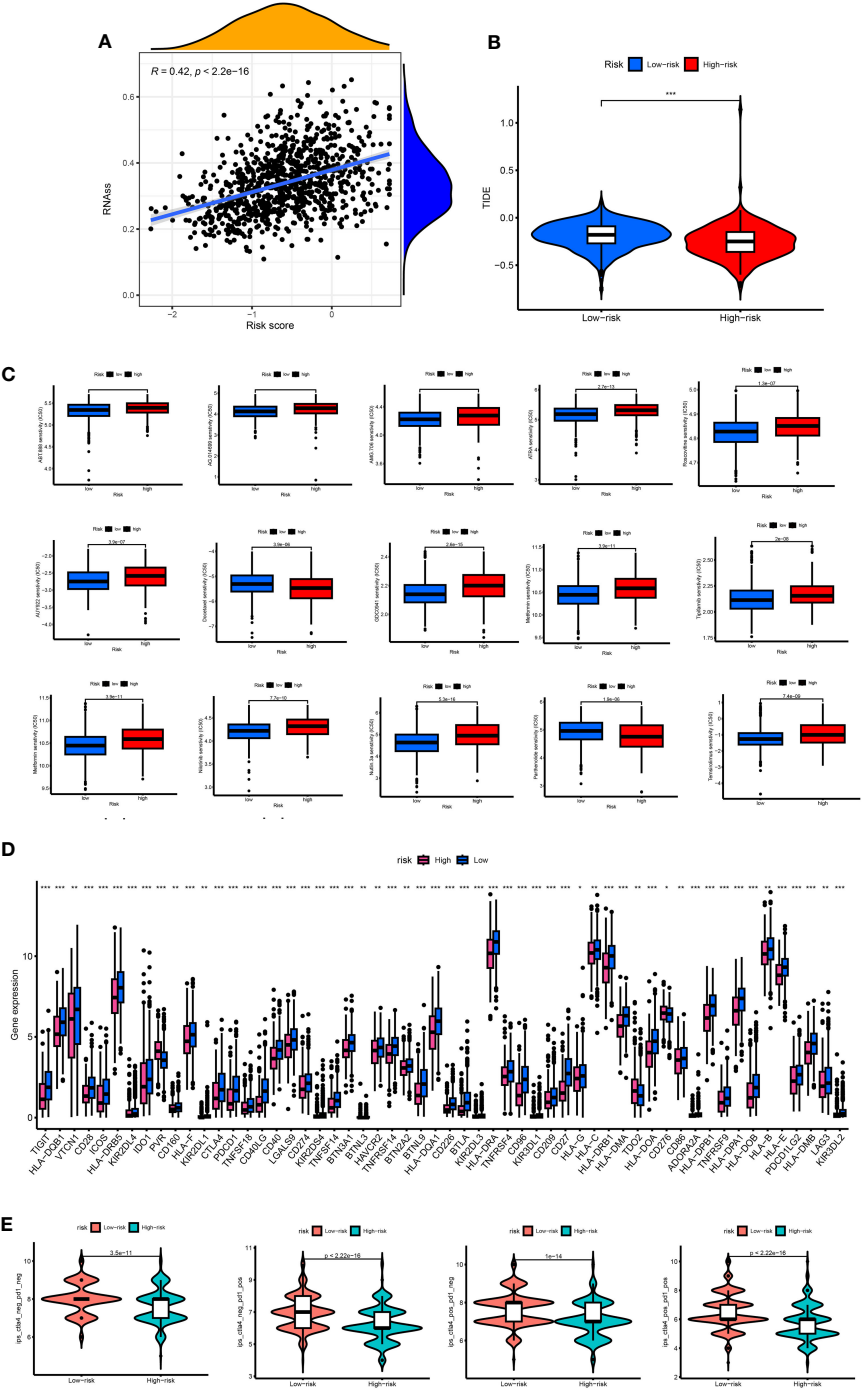
**(A)** Associates between the CSC index and the risk score. **(B)** TIDE score between high- and low-risk group. **(C)** The boxplots for the drug sensitivity analysis. **(D)** Differential expression analysis of the immune checkpoint genes between the high-risk and low-risk groups. IC50, the half-maximal inhibitory concentration; *, p < 0.05; **, p < 0.01; ***, p < 0.001. **(E)** The difference between anti-PD1 treatment and anti-CTLA-4 treatment between high and low risk groups.

There was a positive correlation between RS and stem cells. The higher the RS, the higher the content of stem cells (p < 0.05) (**Figure 9A**). The TIDE score of the high-risk group was lower than that of the low-risk group, and the tumor immune escape ability was weak (**Figure 9B**). The ESTIMATE results showed that the stromal immunity and estimated scores of the high-risk group were low (**Figure 9C**). The drug treatment of breast cancer has broad research prospects and has attracted much attention. Therefore, the IC50 value of chemotherapeutic drugs for BC was calculated, and the relationship between RS and drug resistance was analyzed. We noted that in addition to docetaxel (microtubule depolymerization inhibitors) and parthenolide (NF-κB inhibitors) in high-risk patients with lower IC50. In contrast, other drugs [ABT.888 (Veliparib, PARP inhibitors)], AG.014699 (Rucaparib, PARP inhibitors), AMG.706 (Motesanib, VEGFR inhibitors), ATRA, AUY922 (Luminespib, HSP90 inhibitors), GDC0941 (Pictilisib, PI3K inhibitors), Metformin, Methotrexate, Nilotinib, Nutlin.3a (MDM2 inhibitors), Roscovitine, Temsirolimus, and Tipifarnib) had lower IC50 in low-risk patients (**Figure 9D**). We performed immune checkpoint analysis on high-risk and low-risk groups to explore the precise use of immune checkpoint inhibitors (ICI) in breast cancer patients. Except for the high expression of the PVR gene in the high-risk group, the other immune checkpoint genes were increased in the low-risk group. The IPS of the low-risk group was significantly higher than that of the high-risk group, and the immunotherapy efficacy of the low-risk group was better (**Figure 9E**).

### 3.9 Knockdown of TMEM45A and SHCBP1 inhibited BC cell proliferation

We conducted a search on the DepMap Portal to assess the expression levels of TMEM45A and SHCBP1 in various breast cancer cell lines. Ultimately, we observed that these two genes exhibited significantly high expression in the MDA-MB-468 cell line (**Supplementary Figure 1**). In our study, we constructed a predictive model using six dual sulfur death-related genes. We selected the high-risk genes TMEM45A and SHCBP1 in our model to validate the potential for targeted gene therapy. TCGA and GEO data analyses revealed that TMEM45A and SHCBP1 were highly expressed in breast cancer. Therefore, we knocked down TMEM45A and SHCBP1 and further investigated the role of TMEM45A and SHCBP1 in MDA-MB-468 breast cancer cells *in vitro*.

**Figure 10:**
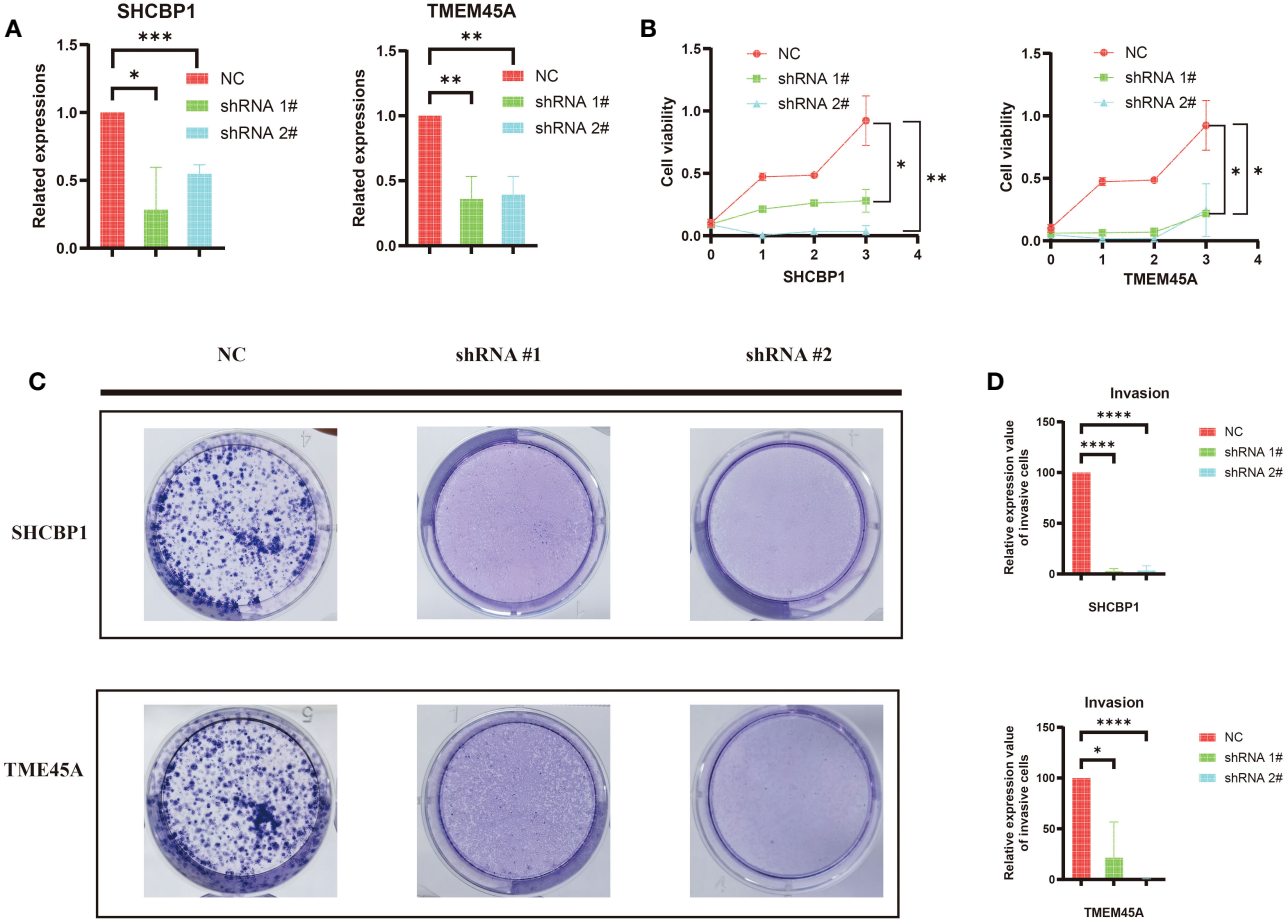
**(A)** The efficiency of silencing TMEM45A and SHCBP1 was indicated by RT-qPCR in MDA-MB-468 cell lines. **(B)** The MTT assay revealed that, in comparison to the control group, the proliferation capability of the MDA-MB-468 cell line with TMEM45A and SHCBP1 knockdown significantly diminished. **(C, D)** The clonogenic assay revealed that the depletion of TMEM45A and SHCBP1 attenuated the proliferative capacity of MDA-MB-468 cells. The data is presented as the mean from at least three independent experiments. (*p<0.05; **p<0.01; ***p< 0.001; ****p< 0.0001).

First, we established MDA-MB-468 shTMEM45A and shSHCBP1 cell lines through lentiviral transduction. As shown in **Figure 10A**, the knockdown of TMEM45A and SHCBP1 in MDA-MB-468 was satisfactory. We further investigated the impact of TMEM45A and SHCBP1 knockdown on the functionality of breast cancer cells through CCK-8 and colony formation assays to determine their effects on BRCA cell proliferation.

As expected, the CCK-8 assay (**Figure 10B**) showed that the knockdown of TMEM45A and SHCBP1 in MDAMB468 similarly inhibited the proliferation of breast cancer cells. The colony formation assays (**Figure 10C, D**) demonstrated that the knockdown of TMEM45A and SHCBP1 significantly and independently inhibited cell proliferation (p < 0.05). These results collectively indicate that TMEM45A and SHCBP1 influence the proliferation of BC cells.

### 3.10 Sequencing and functional enrichment analysis

**Figure 11:**
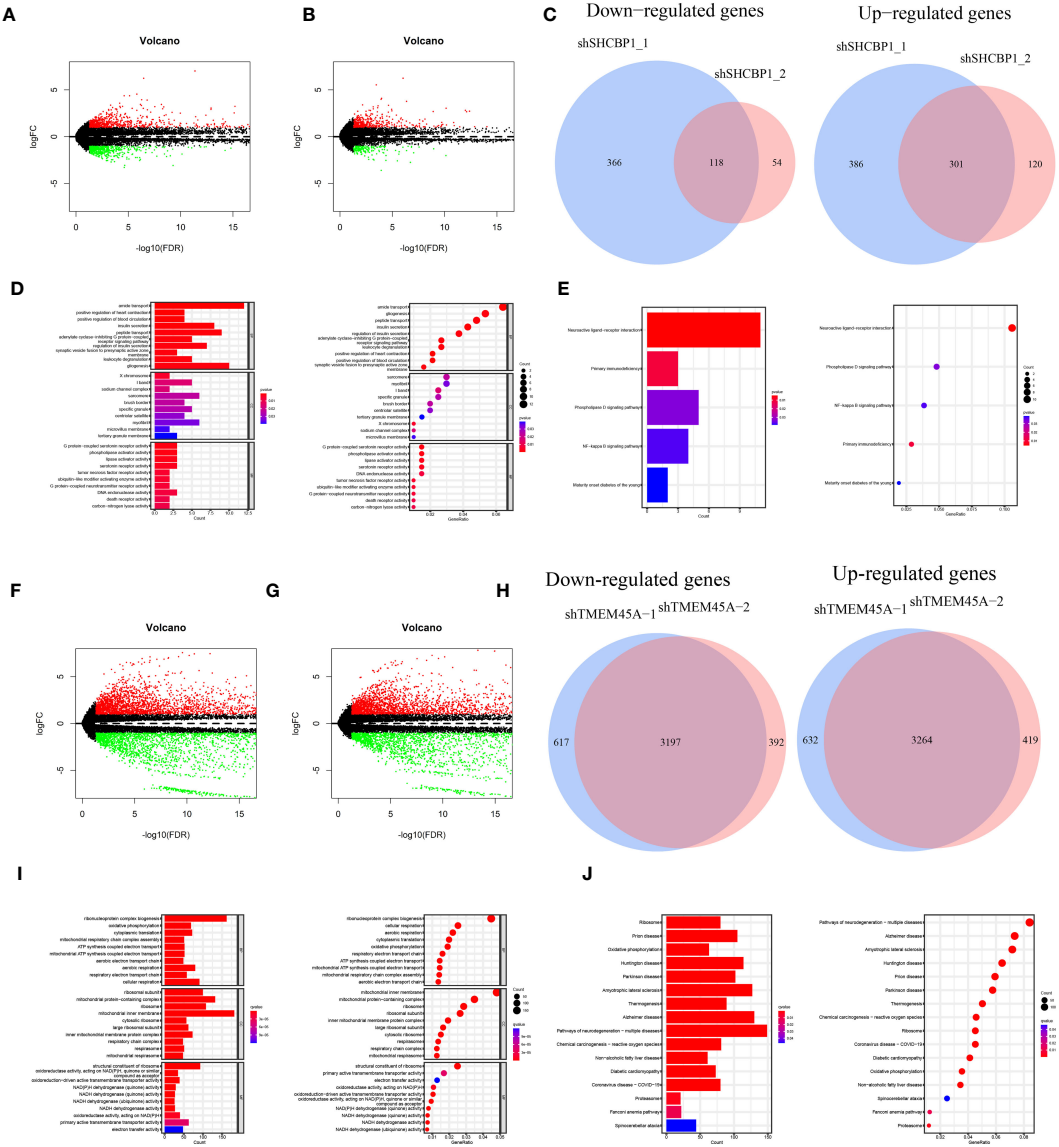
The volcano plot displays the differentially expressed genes between shSHCBP1_1 **(A)** and shSHCBP1_2 **(B)** compared to the NC group. **(C)** The Venn diagram shows the overlap in differentially expressed genes between shSHCBP1_1 and shSHCBP1_2 compared to the NC group. **(D, E)** GO and KEGG Enrichment Analysis of Overlapping Differentially Expressed Genes in shSHCBP1_1 and shSHCBP1_2 vs. NC. The volcano plot displays the differentially expressed genes between shTMEM45A_1 **(F)** and shTMEM45A_2 **(G)** compared to the NC group. **(H)** The Venn diagram shows the overlap in differentially expressed genes between shTMEM45A_1 and shTMEM45A_2 compared to the NC group. **(I, J)** GO and KEGG Enrichment Analysis of Overlapping Differentially Expressed Genes in shTMEM45A_1 and shTMEM45A_2 vs. NC.

The knockdown of SHCBP1 and TMEM45A was achieved by generating shSHCBP1_1, shSHCBP1_2, shTMEM45A_1, and shTMEM45A_2. Differential analysis was conducted using the average counts from three replicate sequencing experiments (**Supplementary Table 6**). shSHCBP1_1 exhibited differential expression in 1171 genes (687 upregulated, 484 downregulated) compared to the NC group, while shSHCBP1_2 showed differential expression in 593 genes (421 upregulated, 172 downregulated) compared to the NC group (**Figures 11A–C**). shTMEM45A_1 exhibited differential expression in 7710 genes (3896 upregulated, 3814 downregulated) compared to the NC group, while shSHCBP1_2 showed differential expression in 7272 genes (3683 upregulated, 3589 downregulated) compared to the NC group (**Figures 11F–H**). We identified the intersecting genes in the upregulated and downregulated gene sets from the two independent replicate experiments and subjected these genes to GO and KEGG enrichment analyses (**Figures 11D, E, I, J**).

## 4 Discussion

Regulated cell death (RCD) is a type of cell death controlled by specific molecular pathways and regulated by genetic or pharmacological manipulation (20). Recently, disulfidptosis has been defined as a new RCD. Previous studies have suggested that SLC7A11-mediated cystine intake is critical in promoting glutathione biosynthesis and inhibiting oxidative stress and ferroptosis. Subsequently, SLC7A11 was found to significantly promote cell death under glucose starvation (21–23). Subsequently, it was found that SLC7A11-mediated reduction of cystine to cysteine was highly dependent on the reduced nicotinamide adenine dinucleotide phosphate (NADPH) produced by the glucose–pentose phosphate pathway (24). Recently, Liu et al. proposed that disulfidptosis is an abnormal accumulation of disulfides, such as cystine, which induces disulfide stress, causes disulfide bond cross-linking and cytoskeleton contraction of the actin cytoskeleton, and ultimately induces cell death6. Not only does RCD play a key role in body development and cell homeostasis, but its dysregulation is also causally linked to many diseases, including cancer. Escape from cell death is considered to be one of the core markers of cancer. The importance of other RCDs in BC has been revealed, but the role of disulfidptosis in BC remains unclear (25–29). Our study explored the importance of disulfidptosis in predicting the prognosis, survival time, immunotherapy response, and chemosensitivity of BC patients. This result may lay the foundation for precisely treating BC breast protrusion-related diseases.

We first performed a differential gene expression analysis, gene copy number mutation between tumor and normal tissues, and tumor mutation load analysis on 11 DGs. We found that, except for NCKAP1, the remaining DGs had significant expression differences between the two groups. Most of them had increased gene expression in breast cancer, meaning these genes may be involved in some BC generation and development processes. In addition to the decrease in CYFIP1 and WASF2 gene copy numbers in tumor samples, the copy number of other DGs increased to varying degrees, consistent with the decline in CYFIP1 and WASF2 gene expression in BC patients. It has been reported that ABI2 can promote the growth and metastasis of HCC. In BC patients, the ABI2 gene copy number increased. Still, gene expression decreased, indicating that there may be other mechanisms in the body, such as methylation, acetylation, ubiquitination, and so on, to regulate ABI2 expression. Among the 911 breast cancer mutation data samples, only 33 had DG mutations. The highest mutation probability was 1% for NCKAP1 and CYFIP1. DGs showed more specific genetic stability than the 53% mutation rate of high-mutation genes, such as TP53 and PIK3CA (30). K-M survival analysis showed that the expression levels of SLC7A11, SLC3A2, RPN1, NCKAP1, BRK1, ACTR2, ACTR3, and RAC1 could independently predict the prognosis of patients (p < 0.05). Among these 11 DGs, SLC7A11 can affect non-small cell lung cancer (31), renal cell carcinoma (32), prostate cancer progression through ferroptosis, and WASF2 is associated with poor ovarian cancer prognosis (33). These results further indicate that DGs play an important role in the development and progression of tumors.

Consensus clustering is a standard unsupervised clustering method for cancer subtype classification research. It can distinguish samples into several subtypes according to different omics datasets to find new disease subtypes or compare different subtypes (34–36). We consistently clustered 11 prognostic DGs in the TCGA and GEO breast cancer data and identified two DG clusters. The OS of BC patients was statistically different between the two clusters, and the OS of group A was longer than that of group B. GSVA analysis showed that some pathways were enriched differently between the two DG groups, such as arachidonic acid metabolism, ubiquitin-mediated proteolysis, and taurine and hypotaurine metabolism. The ssGSEA analysis showed that most of the immune cells in group A were widely enriched and infiltrated, which may inhibit tumor cells through an immune response. In contrast, the lack of immune cells and immunosuppression in group B may be related to the poor prognosis of patients. PCA analysis showed a considerable difference between groups A and B.

A total of 225 DRGs were obtained, and GO and KEGG enrichment analyses were performed on these genes. GO analysis showed that these DRGs were mainly enriched in microtubule binding, tubulin binding, and other biological behaviors closely related to the construction of the cytoskeleton. Therefore, it can be speculated that these DRGs may mediate changes in the cytoskeleton and cause cell death, which is consistent with previous studies 6. Furthermore, through KEGG enrichment analysis, these differential genes were mainly enriched in signal transduction pathways, such as the cell cycle, PPAR signaling pathway, ECM-receptor interaction, and other pathways, indicating that intercellular interaction may be a critical link in disulfidptosis-induced cell death.

One-hundred-and-fourteen of the 225 differential genes were associated with BC prognosis. According to the expression of these 114 DRGs, breast cancer was divided into three subtypes by consensus clustering. The prognosis of group A was the best, followed by group B, and the prognosis of group C was relatively poor. In this study, we further developed six DRGs (SHCBP, TMEM45A, PIGR, IGLV6-57, TCN1, GFRA1) to construct RS to predict the prognosis of BC patients and used TCGA, GEO separated and TCGA and GEO joint databases to evaluate the prognostic value of RS through a survival curve, RS map, survival state map, and heatmap. RS = (0.1917 * SHCBP1 + 0.0836 * TMEM45A-0.0721 * PIGR-0.1633 * IGLV6-57-0.0489 * TCN1-0.0676 * GFRA1). SHCBP1 has been previously reported as an immune-related biomarker for cancer diagnosis and prognosis and a potential therapeutic target for tumor immunotherapy (37). Jing et al. proposed that TMEM45A can be used as an oncogene in ovarian cancer and that inhibition of TMEM45A may be a therapeutic strategy for ovarian cancer. Wichitra et al. proposed that M1 macrophages can cause high expression of PIGR in breast cancer cells, and high expression of polymeric immunoglobulin receptor (PIGR) in breast cancer is associated with an increased 5-year survival rate (38), indicating that PIGR may be a protective factor for breast cancer. IGLV6-57 is also widely used in cancer diagnosis (39). TCN1 may play a carcinogenic role in colorectal cancer by regulating the ITGB4 signaling pathway leading to cytoskeleton damage and promoting cell death (40). This type of cell death may be disulfidptosis. Sunil Bhakta et al. proposed that GFRA1 is associated with targeted therapy for hormone receptor–positive breast cancer (41). The six genes constructing the model are inseparable from regulating tumor life activities.

The area under the ROC curve (AUC) was used to evaluate the predictive ability of RS for patient prognosis (42). A considerable AUC indicates that the model has good classification ability and can compare features (43). The AUC of our model was 0.762, 0.734, and 0.758 at one year, three years, and five years, respectively, which was significantly higher than most prediction models. We integrated all the essential information of a series of prognostic models, including author, year, and genetic characteristics, and constructed AUC to verify the diagnostic performance of the model. After comparison, we found that our model had the best diagnostic performance (**Table 1**). **Table 1** shows a metabolic-related 4-gene prognostic model with AUCs of 0.764, 0.689, and 0.612 for 1-year, 3-year, and 5-year, respectively (44). Another pyroptosis model consisted of 16 genes with AUC of 0.756, 0.752, and 0.723 for 1, 3, and 5 years (45). Its diagnostic value is almost the same as our model, but it is composed of 16 genes, while our model has only six genes that can be better applied in clinical practice. In two cuproptosis-related prognostic models, 1-year, 3-year, and 5-year AUC values were 0.685, 0.678, and 0.678 (46), and for another 1-, 3-, and 5-year model, the AUCs were 0.554, 0.527, and 0.649 (47). The AUC of the ferroptosis model constructed by Zhu et al. was 0.821, 0.678, and 0.651 in 1-, 3-, and 5-years (48). The AUCs of the ferroptosis model produced by Zhu et al. were 0.821, 0.678, and 0.651 in 1-, 3-, and 5-year models. Although it had a good prediction effect in the first year, our model AUC was above 0.734, which was more stable. We also list other models more concerned with short-term survival rates, such as the AUC of the necroptosis-related model at 1-, 2-, and 3-year patients, which were 0.701, 0.716, and 0.708 (49). The AUC of the necroptosis-related prognostic model constructed by Chen et al. in 1-, 3-, and 5-year patients were 0.731, 0.643, and 0.641, respectively (50). Our prognostic model has adequate predictive value. Our model involves only six genes, while other models tend to have more genes. To a certain extent, our RS model is more convenient to use. The C-index of the nomogram was 0.762 (95% CI: 0.711–0.813), indicating that the predicted results of 1-, 3-, and 5-year patients were consistent with the actual results. The expression of DGs in the high-risk group was significantly increased, suggesting that genes have an adverse effect on the prognoses of patients. Therefore, the prognostic model constructed by DRGs is reliable and accurate. On the other hand, it also shows the critical role of DGs in the occurrence, development, and life processes of tumors. In summary, the model we constructed can be considered a suitable prognostic signal, and its mechanism of action in BC deserves further exploration and verification.

**Table 1:** The area under the ROC curve (AUC) showed the sensitivity and specificity of the known gene signatures in predicting the prognosis of BC patients.

Article	Year	RCD	Model Gene	AUC
Our	2023	Disulfidptosis	6	0.762 (1-year), 0.734 (3-year), 0.758 (5-year)
Lu, Liu, and Zhang 2022 (44)	2022	Metabolic	4	0.764 (1-year), 0.689 (3-year), 0.612 (5-year)
Chen, Luo, et al., 2022 (45)	2022	Pyroptosis	16	0.756 (1-year), 0.752 (3-year), 0.723 (5-year)
Li et al., 2022 (46)	2022	Cuproptosis	6	0.685 (1-year), 0.678 (3-year), 0.678 (5-year)
Sha et al., 2022 (47)	2022	Cuproptosis	2	0.554 (1-year), 0.527 (3-year), 0.649 (5-year)
Zhu et al., 2022 (48)	2022	Ferroptosis	6	0.821 (1-year), 0.678 (3-year), 0.651 (5-year)
Yu et al., 2022 (49)	2022	Necroptosis	6	0.701 (1-year), 0.716 (2-year), 0.708 (3-year)
Chen, Yang, et al., 2022 (50)	2022	Necroptosis	7	0.731 (1-year), 0.643 (3-year), 0.641 (5-year)

Subsequently, we found the immune factors that determined the prognoses of the high- and low-risk groups through the immune analysis of the patients. The high-risk group had a large number of macrophage M2 cell infiltrations. Macrophages M2 increase is associated with tumor growth and poor prognosis of cancer 48, and are considered essential biomarkers in cancer diagnosis and a potential target for cancer treatment (51, 52). T cells CD8 is a significant member of the low-risk group. In previous reports, T cell CD8 can prevent tumor growth and promote immune response and immunotherapy (53). This may be one of the reasons for the survival difference between high- and low-risk groups. The TMB study showed that PIK3CA had the highest mutation frequency in the low-risk samples, while TP53 had the highest mutation frequency in the high-risk groups. The mutation rate of TP53 in patients with an increased risk of BC is 43%, while the mutation rate of TP53 in patients with low risk is only 29%. Previous studies have reported that the mutation of the TP53 gene is related to the poor therapeutic effect and prognosis of BC (54, 55), confirming the poor prognosis of patients with a high TP53 mutation rate in our high-risk group.

Tumor treatment is an essential field of concern. Through IC 50 screening analysis of potential chemical drugs, we realized that low-risk patients might be more sensitive to chemotherapy, ABT.888 (Veliparib, PARP inhibitors), AG.014699 (Rucaparib, PARP inhibitors), AMG.706 (Motesanib, VEGFR inhibitors), ATRA, and AUY922 (Luminespib, HSP90 inhibitors). GDC0941 (Pictilisib, PI3K inhibitors), Metformin, Methotrexate, Nilotinib, Nutlin.3a (MDM2 inhibitors), Roscovitine, Temsirolimus, Tipifarnib, and other drugs had a lower IC50 in the low-risk group. High-risk patients resisted PARP and VEGFR inhibitors and other drugs and were sensitive to docetaxel (a microtubule depolymerization inhibitor) and parthenolide (NF-κB inhibitors). Immunotherapy also occupies a crucial position in the treatment of BC patients. The ESTIMATE results showed that the stromal immunity and estimated scores of the high-risk group were low. The TIDE analysis showed that the TIDE immune escape score of the high-risk group was low. The combination of PD-1 and PDL-1 caused T cells to lose the ability to attack cancer cells, resulting in the immune escape of tumor cells. The expression of immune checkpoint genes, such as PD-1 and PDL-1, in high-risk patients, was low, which may be the reason for the low TIDE immune escape score in the high-risk group. However, based on the difference in immune checkpoints between the high- and low-risk groups, distinguished by the RS model constructed by DRGs, we found that the expression of immune checkpoint genes in the high-risk group was significantly lower than that in the low-risk group. It is speculated that patients in the low-risk group are more likely to induce an antitumor immune response through immune genes, thus benefiting from immunotherapy, which is consistent with the high Stromal Score, Immune Score, and ESTIMATE Score results of the low-risk population in the previous article. TMB is a reliable biomarker for predicting treatment outcomes in cancer patients treated with ICI (56, 57). This is consistent with our earlier results that ‘RS is significantly associated with TMB. Patients in the high-risk group had higher TMB, antitumor immune dysfunction, poor ICI treatment, and poor prognosis. In summary, combined with drug sensitivity and immune efficacy analysis, we predict that low-risk patients will benefit more from combining chemotherapy and immunotherapy, providing a basis for individualized treatment of BC patients. More drugs are worthy of selection and development for low-risk patients. The effects of chemotherapy and immunotherapy in the high-risk group were poor. The high expression of disulfidptosis genes (e.g., SLC3A2, RPN1, BRK1, ACTR2, ACTR3, SLC7A11, and NCKAP1) in the KM analysis of breast cancer indicated the poor prognosis of patients. The difference analysis between the high- and low-risk groups differed. These genes were highly expressed in the high-risk group. Through our study, targeted therapy of the disulfidptosis gene will benefit the high-risk group.

In addition, we conducted *in vitro* validation through cell formation assays and CCK-8 on the high-risk genes (TMEM45A and SHCBP1) within the RS model. This initial validation provided preliminary evidence of the feasibility of utilizing these two genes for targeted therapy in breast cancer (BC). Subsequently, we conducted cellular sequencing following the gene knockout of TMEM45A and SHCBP1 to infer their roles in the biological mechanisms.

This study systematically analyzed the role of disulfidptosis-related genes in the prognosis of breast cancer and their correlation with the tumor microenvironment and clinical characteristics and constructed a better prognosis prediction model. The model performs well in predicting the survival outcome, tumor immune microenvironment, and immunotherapy response of BC patients. Furthermore, it expounds on its correlation with TMB, immunity, and clinical treatment (chemotherapy and immunotherapy), providing a reference for individualized and precise breast cancer treatment. Finally, Experimental validation and sequencing were also conducted to substantiate these findings.

## 5 Conclusion

This study has the following contributions. First, this study is the first to identify the subtypes associated with disulfidptosis and to create a predictive model based on breast cancer DRGs. Although disulfidptosis differs from other recognized cell death methods, it may provide new therapeutic possibilities for cancer treatment. Second, a variety of different technologies and databases are used to improve the reliability of the results. We also defined subtypes associated with disulfidptosis and created a predictive model for the screening and testing processes. Finally, we first proposed a disulfidptosis gene-targeted therapy for high-risk BC groups. We intend to gather further patient samples during subsequent clinical investigations to substantiate the dependability of our model.

## Data availability statement

The datasets presented in this study can be found in online repositories. The names of the repository/repositories and accession number(s) can be found within the article/**Supplementary Materials**. The sequencing raw data is stored in the GEO database under GSE246486.

## Ethics statement

Ethical approval was not required for the studies on humans in accordance with the local legislation and institutional requirements because only commercially available established cell lines were used.

## Author contributions

JHL, ZR, and MR designed and conceived this project; XW and JY completed these experiments; JS, JHL, and XW interpreted and analyzed the data; JHL and JCL revised the manuscript. All authors contributed to the article and approved the submitted version.
