# Supplementary figures and images for: Identification of disulfidptosis-related subtypes, characterization of tumor microenvironment infiltration, and development of a prognosis model in breast cancer
Source: Front Immunol. 2023 Nov 15;14:1198826. doi: 10.3389/fimmu.2023.1198826 (PMC10684933; doi:10.3389/fimmu.2023.1198826)

## Slide 1
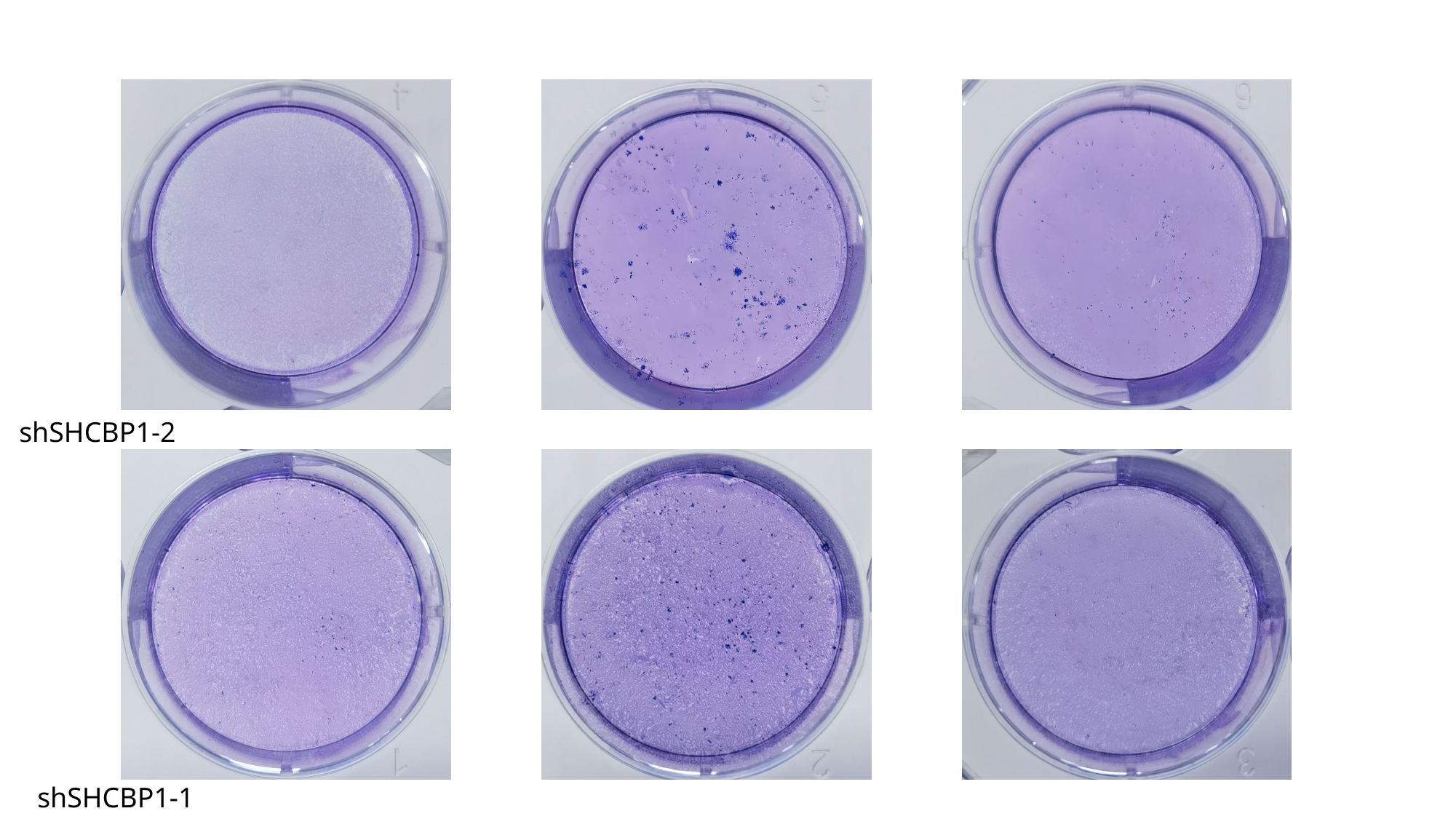

shSHCBP1-2
shSHCBP1-1

## Slide 2
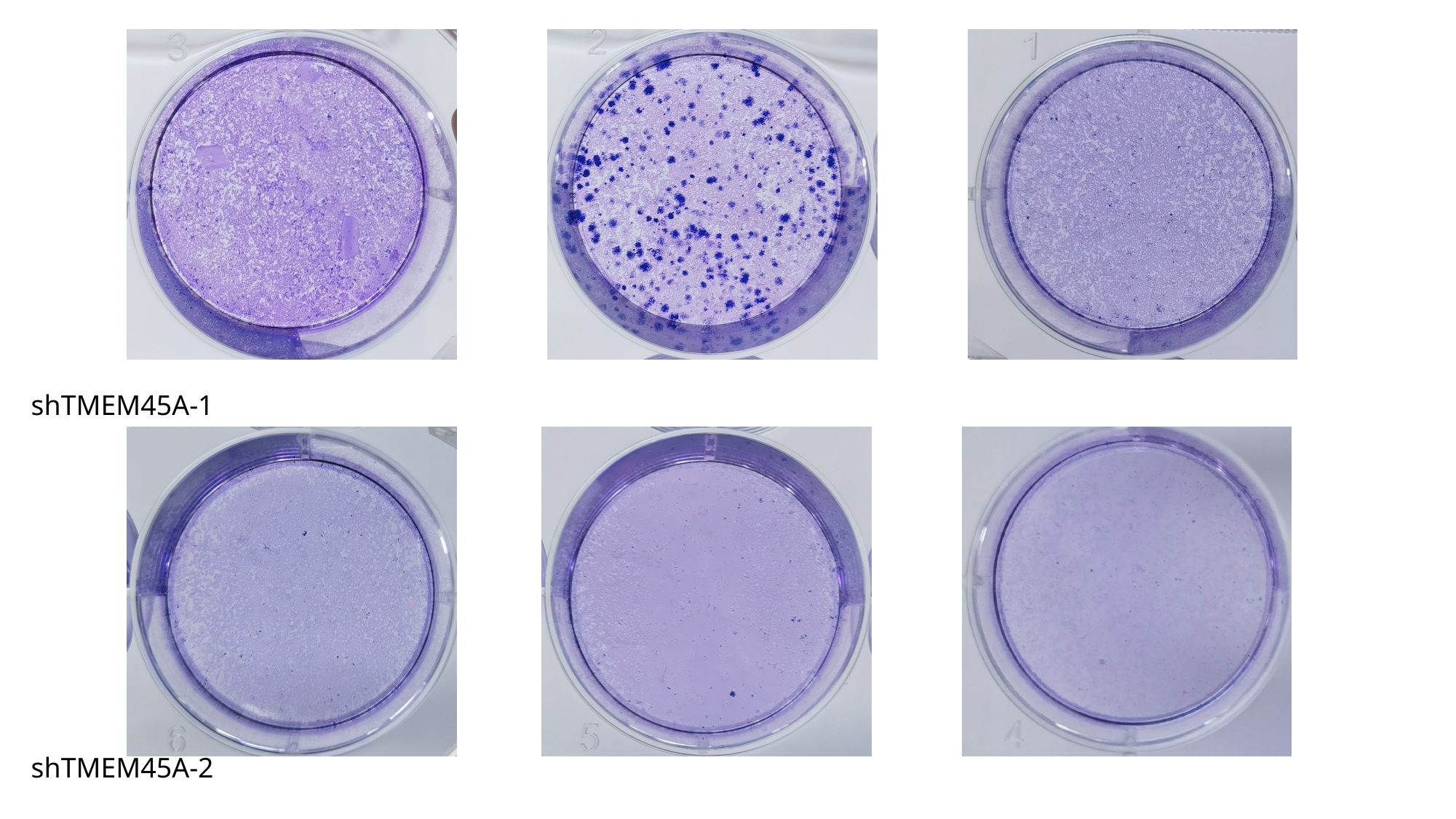

shTMEM45A-1
shTMEM45A-2

## Slide 3
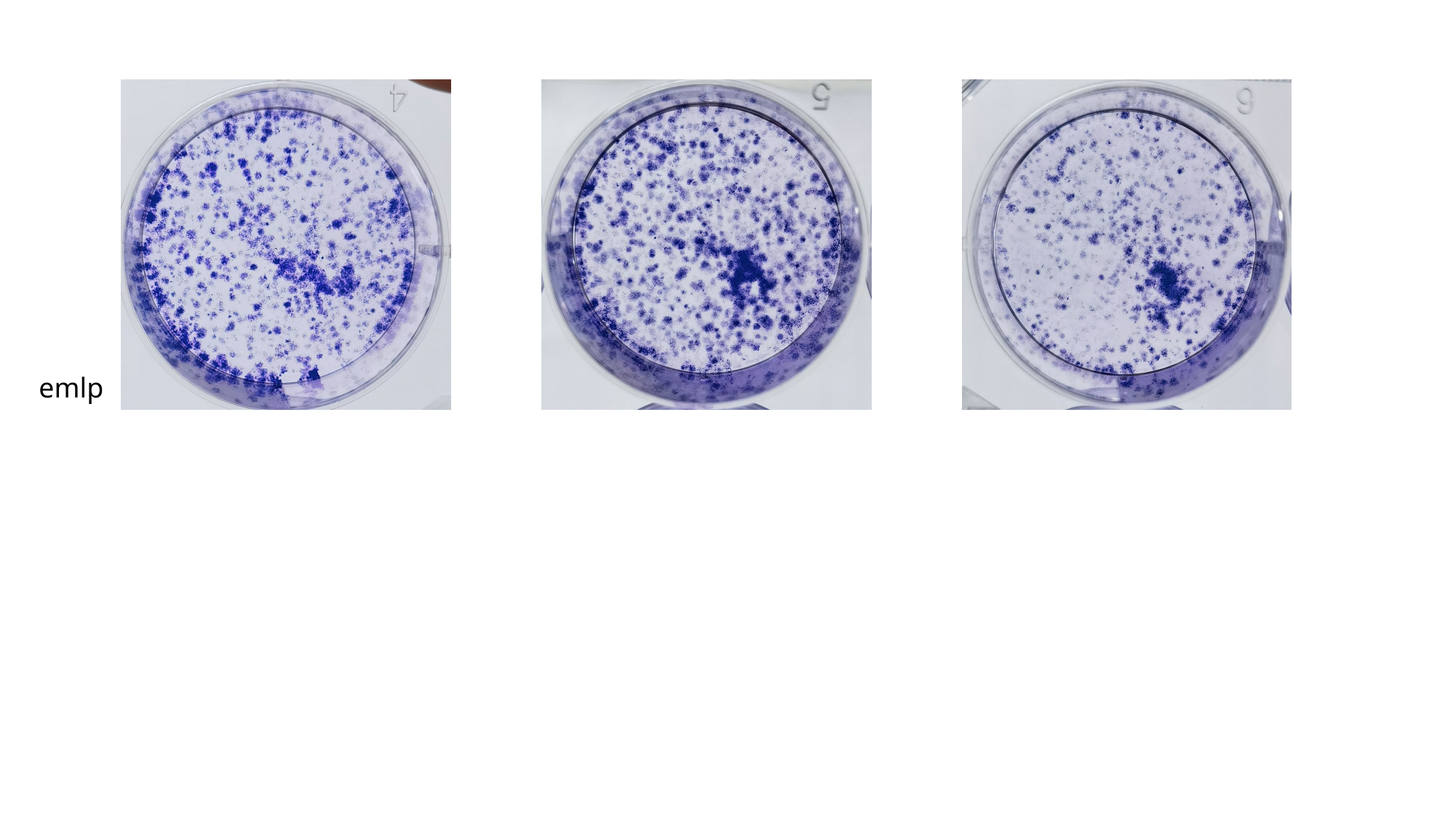

emlp

Supplement: Supplementary file 1 [file DataSheet_1.zip › colony assay.pptx]
