# Supplementary material for: Identification of disulfidptosis-related subtypes, characterization of tumor microenvironment infiltration, and development of a prognosis model in breast cancer
Source: Front Immunol. 2023 Nov 15;14:1198826. doi: 10.3389/fimmu.2023.1198826 (PMC10684933; doi:10.3389/fimmu.2023.1198826)
Supplement: Supplementary file 3 [file Table_1.doc]

| Abbreviation | **English full name** |
| --- | --- |
| BC | Breast cancer |
| DG | disulfidptosis gene |
| TCGA | the Cancer Genome Atlas |
| GEO | Gene Expression Omnibus database |
| GSVA | gene set variation analysis |
| ssGSEA | Single sample gene set enrichment analysis |
| RS | risk score |
| OS | overall survival |
| TMB | Tumor mutation burden (TMB) |
| NADPH | nicotinamide adenine dinucleotide phosphate |
| WRC | the WAVE regulatory complex |
| CNV | Copy Number Variation |
| GSVA | Gene set variation analysis |
| KEGG | The Kyoto Encyclopedia of Genes and Genomes |
| LASSO | The least absolute shrinkage and selection operator |
| ROC | receiver operating characteristic |
| SSE | the sum of the squared errors |
| IPS | Immunophenoscore |
| RS | Risk score |
| IC50 | the half inhibitory concentration |
| IPS | immunophenotype score |
| CTLA-4 | cytotoxic T lymphocyte antigen 4 |
| PD-1 | programmed cell death protein 1 |

**Supplementary Table 1.** Abbreviation comparison table.
