# Supplementary material for: Identification of disulfidptosis-related subtypes, characterization of tumor microenvironment infiltration, and development of a prognosis model in breast cancer
Source: Front Immunol. 2023 Nov 15;14:1198826. doi: 10.3389/fimmu.2023.1198826 (PMC10684933; doi:10.3389/fimmu.2023.1198826)
Supplement: Supplementary file 4 [file Table_2.docx]

| Id | HR | HR.95L | HR.95H | pvalue | km |
| --- | --- | --- | --- | --- | --- |
| SLC7A11 | 1.169672 | 1.040811 | 1.314486 | 0.008498 | 0.00105 |
| SLC3A2 | 1.256835 | 1.000827 | 1.578328 | 0.049174 | 0.009417 |
| RPN1 | 1.029956 | 0.792725 | 1.338181 | 0.825113 | 0.044397 |
| NCKAP1 | 1.407883 | 1.136905 | 1.743448 | 0.001711 | 0.001912 |
| WASF2 | 0.959065 | 0.77494 | 1.186938 | 0.700766 | 0.111566 |
| CYFIP1 | 1.061738 | 0.834287 | 1.3512 | 0.626236 | 0.060581 |
| ABI2 | 0.958089 | 0.747853 | 1.227426 | 0.734811 | 0.063289 |
| BRK1 | 1.189878 | 0.898088 | 1.576471 | 0.225839 | 0.004314 |
| ACTR2 | 1.237316 | 0.981722 | 1.559453 | 0.071276 | 7.80E-06 |
| ACTR3 | 1.16443 | 0.903103 | 1.501376 | 0.240401 | 0.001559 |
| RAC1 | 1.408725 | 0.994198 | 1.996088 | 0.05395 | 0.017743 |

**Supplementary Table 2**. K-M survival analysis of disulfidptosis genes in breast cancer patients.
