# Supplementary material for: Identification of disulfidptosis-related subtypes, characterization of tumor microenvironment infiltration, and development of a prognosis model in breast cancer
Source: Front Immunol. 2023 Nov 15;14:1198826. doi: 10.3389/fimmu.2023.1198826 (PMC10684933; doi:10.3389/fimmu.2023.1198826)
Supplement: Supplementary file 5 [file Table_3.docx]

| gene | HR | lower.95 | upper.95 | pvalue |
| --- | --- | --- | --- | --- |
| SLC7A11 | 1.169676944 | 1.040816339 | 1.314491426 | 0.008495 |
| TXNRD1 | 1.308968819 | 1.102777189 | 1.553713104 | 0.00208 |
| PPAT | 1.233179607 | 1.028420308 | 1.478706644 | 0.023669 |
| GAS2L3 | 1.25054338 | 1.073237653 | 1.457141149 | 0.004157 |
| ATP13A3 | 1.247664479 | 1.042056423 | 1.493841041 | 0.016025 |
| KIF21A | 1.352639869 | 1.157000745 | 1.581359927 | 0.000151 |
| SMC4 | 1.162606182 | 1.014789152 | 1.331954655 | 0.029889 |
| MAD2L1 | 1.204999737 | 1.053994215 | 1.377639788 | 0.006338 |
| CENPE | 1.213538247 | 1.06961864 | 1.376822561 | 0.002657 |
| ARHGAP11A | 1.280037007 | 1.097042971 | 1.493555661 | 0.001709 |
| ECT2 | 1.165693474 | 1.00700827 | 1.349384424 | 0.040024 |
| DDIAS | 1.19652954 | 1.026116696 | 1.395243783 | 0.022088 |
| NCAPG | 1.199958816 | 1.066560487 | 1.350041725 | 0.002432 |
| CKAP2L | 1.183986426 | 1.036891164 | 1.351948889 | 0.012589 |
| CCNA2 | 1.144321128 | 1.021467415 | 1.281950677 | 0.019991 |
| DEPDC1 | 1.131199353 | 1.006673079 | 1.271129628 | 0.03829 |
| DIAPH3 | 1.200535665 | 1.038941387 | 1.387263903 | 0.013216 |
| STIL | 1.196149602 | 1.041166288 | 1.374203032 | 0.011414 |
| AMD1 | 1.198360445 | 1.029131807 | 1.395416743 | 0.019824 |
| BUB1 | 1.186914605 | 1.044560116 | 1.348669415 | 0.00857 |
| ASPM | 1.165545051 | 1.036839896 | 1.310226652 | 0.01029 |
| KIF14 | 1.168021112 | 1.044382366 | 1.306296775 | 0.006515 |
| KIF23 | 1.169934081 | 1.027683693 | 1.331874548 | 0.017653 |
| ANLN | 1.221328147 | 1.103133459 | 1.352186746 | 0.000118 |
| DEPDC1B | 1.19044035 | 1.03595112 | 1.367968236 | 0.013972 |
| MCM10 | 1.155150626 | 1.03583457 | 1.288210499 | 0.009517 |
| PRKDC | 1.174115428 | 1.013260362 | 1.360506232 | 0.032745 |
| ATAD2 | 1.163820626 | 1.029369934 | 1.315832533 | 0.01543 |
| FOXM1 | 1.111566301 | 1.007972112 | 1.225807368 | 0.034086 |
| SHCBP1 | 1.317471065 | 1.155820006 | 1.501730371 | 0.000037 |
| CNOT1 | 1.181322938 | 1.023090676 | 1.364027566 | 0.023141 |
| BUB1B | 1.200482002 | 1.063346671 | 1.355303096 | 0.003153 |
| HMMR | 1.205590466 | 1.064804528 | 1.364990789 | 0.003167 |
| ZNF367 | 1.204084945 | 1.056166364 | 1.372719871 | 0.005485 |
| CEP55 | 1.146065787 | 1.028926329 | 1.27654114 | 0.0132 |
| RACGAP1 | 1.268065197 | 1.106518319 | 1.453197218 | 0.000636 |
| TFRC | 1.163876788 | 1.019939845 | 1.32812654 | 0.024253 |
| KIF11 | 1.163223105 | 1.026617661 | 1.318005762 | 0.017687 |
| ORC1 | 1.14766419 | 1.00136797 | 1.315333756 | 0.047747 |
| HLTF | 1.176766686 | 1.033255932 | 1.340209902 | 0.014168 |
| MKI67 | 1.138709264 | 1.020069361 | 1.271147667 | 0.020671 |
| TPX2 | 1.144424085 | 1.030578509 | 1.270845914 | 0.011623 |
| PIR | 1.193176258 | 1.056680619 | 1.347303581 | 0.00438 |
| CCNE2 | 1.210083842 | 1.074485575 | 1.362794381 | 0.001662 |
| TTK | 1.199854828 | 1.079147845 | 1.334063369 | 0.000757 |
| EXO1 | 1.196419122 | 1.064434395 | 1.344769318 | 0.002638 |
| CDC7 | 1.159943032 | 1.018647207 | 1.3208379 | 0.025173 |
| DLGAP5 | 1.138525062 | 1.019839259 | 1.271023158 | 0.020904 |
| TRIP13 | 1.185716669 | 1.057243818 | 1.329801125 | 0.003599 |
| FAM83D | 1.169679916 | 1.063258497 | 1.286753043 | 0.001281 |
| KIF4A | 1.224031066 | 1.09086858 | 1.373448715 | 0.000582 |
| KLF2 | 0.849504709 | 0.742770595 | 0.971576226 | 0.017271 |
| CCNB2 | 1.16251621 | 1.020885345 | 1.323796002 | 0.023098 |
| TOP2A | 1.113413322 | 1.011091434 | 1.226090127 | 0.028945 |
| NDC80 | 1.134157024 | 1.012762498 | 1.270102476 | 0.029293 |
| BIRC5 | 1.138883648 | 1.023114278 | 1.267752773 | 0.017418 |
| CENPF | 1.153677615 | 1.0273439 | 1.295546739 | 0.015698 |
| PBK | 1.158213352 | 1.040926106 | 1.288716039 | 0.007012 |
| KIF15 | 1.145434554 | 1.01184451 | 1.296661991 | 0.031868 |
| PALM | 0.891327427 | 0.797036541 | 0.996773097 | 0.043735 |
| MCM2 | 1.151060942 | 1.011763874 | 1.309536073 | 0.032543 |
| CDK1 | 1.156875247 | 1.022391362 | 1.309048949 | 0.020823 |
| CDCA8 | 1.156209004 | 1.025893972 | 1.30307741 | 0.017361 |
| CENPA | 1.130110028 | 1.005207253 | 1.27053269 | 0.04067 |
| RRM2 | 1.162231563 | 1.040671647 | 1.297990784 | 0.007648 |
| MAP2 | 1.128791409 | 1.009201208 | 1.262553034 | 0.033984 |
| CDC45 | 1.206673753 | 1.063401181 | 1.36924951 | 0.003578 |
| MFAP4 | 0.914710775 | 0.844798337 | 0.990408913 | 0.027983 |
| NEK2 | 1.149777435 | 1.022535139 | 1.292853517 | 0.019681 |
| KIF18B | 1.154971935 | 1.029795227 | 1.295364491 | 0.013832 |
| CD109 | 1.128831705 | 1.00597452 | 1.266693134 | 0.039276 |
| KIF2C | 1.15519053 | 1.030601894 | 1.294840587 | 0.013225 |
| ACKR1 | 0.894740863 | 0.83627062 | 0.95729922 | 0.001257 |
| CDC6 | 1.106104495 | 1.005163425 | 1.217182325 | 0.038881 |
| PLK1 | 1.247254162 | 1.107617037 | 1.40449532 | 0.000265 |
| NUF2 | 1.180256369 | 1.062253659 | 1.311367662 | 0.002045 |
| MELK | 1.139329784 | 1.02567823 | 1.265574639 | 0.014981 |
| E2F8 | 1.122086797 | 1.003119407 | 1.255163415 | 0.043964 |
| KIF20A | 1.187580091 | 1.059269785 | 1.331432741 | 0.003209 |
| SLC7A5 | 1.152035102 | 1.054575478 | 1.258501554 | 0.0017 |
| APOD | 0.915697632 | 0.86225572 | 0.97245183 | 0.004099 |
| EVL | 0.840056419 | 0.758477524 | 0.930409623 | 0.000826 |
| TMEM45A | 1.165040751 | 1.047009132 | 1.296378331 | 0.005065 |
| IBSP | 1.102604717 | 1.0012717 | 1.214193074 | 0.047055 |
| NGFR | 0.866992436 | 0.782888212 | 0.96013182 | 0.006117 |
| PCP2 | 0.860974659 | 0.772157978 | 0.960007388 | 0.007045 |
| ITM2A | 0.822916799 | 0.743474054 | 0.910848273 | 0.000168 |
| CCL19 | 0.885838969 | 0.835024558 | 0.93974563 | 0.000058 |
| LRG1 | 0.921406488 | 0.865091233 | 0.981387723 | 0.010964 |
| TNN | 0.822603973 | 0.745367171 | 0.907844244 | 0.000104 |
| RAMP3 | 0.871310988 | 0.781586117 | 0.971336134 | 0.012974 |
| F2RL2 | 0.88457104 | 0.814797422 | 0.960319589 | 0.003436 |
| PHYHD1 | 0.90007278 | 0.830750181 | 0.975180057 | 0.010036 |
| PRAME | 1.06097023 | 1.007859081 | 1.116880178 | 0.0239 |
| MMP1 | 1.090627765 | 1.031114815 | 1.153575629 | 0.002444 |
| TFF1 | 0.956509713 | 0.924283373 | 0.989859666 | 0.010996 |
| EGR1 | 0.882898997 | 0.805455661 | 0.96778839 | 0.007837 |
| C7 | 0.900203748 | 0.829265017 | 0.977210869 | 0.012059 |
| IGHD | 0.842335782 | 0.774904312 | 0.915635077 | 0.000056 |
| CD79A | 0.851876352 | 0.785184379 | 0.924233006 | 0.000116 |
| WNK4 | 0.903225061 | 0.837246438 | 0.974403084 | 0.008539 |
| FOSB | 0.917864499 | 0.851640384 | 0.989238244 | 0.024887 |
| CXCL14 | 0.905564372 | 0.851672513 | 0.962866383 | 0.001531 |
| CCL21 | 0.930546699 | 0.871345849 | 0.993769764 | 0.031848 |
| CHAD | 0.931078805 | 0.876028383 | 0.989588646 | 0.021645 |
| IGHM | 0.905099824 | 0.857898179 | 0.954898508 | 0.000263 |
| PIGR | 0.868779323 | 0.810253273 | 0.931532815 | 0.000077 |
| IGKC | 0.894051902 | 0.848109284 | 0.942483261 | 0.000032 |
| CAPN8 | 0.934259316 | 0.8749951 | 0.997537552 | 0.041982 |
| IGLV6-57 | 0.863688424 | 0.812164116 | 0.918481475 | 3.00E-06 |
| TCN1 | 0.907116478 | 0.848863252 | 0.969367332 | 0.003993 |
| SCUBE2 | 0.919219559 | 0.873077625 | 0.967800083 | 0.001348 |
| GFRA1 | 0.93485729 | 0.890118947 | 0.98184423 | 0.007097 |
| LTF | 0.932399637 | 0.892155441 | 0.974459205 | 0.001875 |

**Supplementary Table 3**. Univariate analysis of DRGs in breast cancer patients.
