# Supplementary material for: Identification of disulfidptosis-related subtypes, characterization of tumor microenvironment infiltration, and development of a prognosis model in breast cancer
Source: Front Immunol. 2023 Nov 15;14:1198826. doi: 10.3389/fimmu.2023.1198826 (PMC10684933; doi:10.3389/fimmu.2023.1198826)
Supplement: Supplementary file 6 [file Table_4.docx]

| Gene | Coefficients |
| --- | --- |
| TXNRD1 | 0.000327657 |
| KIF21A | 0.065191643 |
| SHCBP1 | 0.09695489 |
| EVL | -0.008670732 |
| TMEM45A | 0.022204065 |
| PCP2 | -0.012922984 |
| MMP1 | 0.01237786 |
| IGHD | -0.007203875 |
| PIGR | -0.038080743 |
| IGLV6-57 | -0.115320921 |
| TCN1 | -0.009022227 |
| SCUBE2 | -0.025295775 |
| GFRA1 | -0.030306926 |
| LTF | -0.008475325 |

**Supplementary Table 4**. LASSO genes.
