# Supplementary material for: Identification of disulfidptosis-related subtypes, characterization of tumor microenvironment infiltration, and development of a prognosis model in breast cancer
Source: Front Immunol. 2023 Nov 15;14:1198826. doi: 10.3389/fimmu.2023.1198826 (PMC10684933; doi:10.3389/fimmu.2023.1198826)
Supplement: Supplementary file 7 [file Table_5.docx]

|  | oneyearauc | threeyearauc | fiveyearauc | cindex |
| --- | --- | --- | --- | --- |
| 1 | 0.746950507 | 0.684724333 | 0.674010872 | 0.688927667 |
| 2 | 0.744520428 | 0.673627581 | 0.663120509 | 0.671444735 |
| 3 | 0.751131959 | 0.689002543 | 0.66233104 | 0.682284886 |
| 4 | 0.771882808 | 0.701819067 | 0.683351743 | 0.690855756 |
| 5 | 0.738306094 | 0.69195097 | 0.664570283 | 0.681592674 |
| 6 | 0.76217233 | 0.707397655 | 0.67569532 | 0.645469439 |
| 7 | 0.761087453 | 0.690963088 | 0.676086977 | 0.71061707 |
| 8 | 0.74556122 | 0.682699298 | 0.67048987 | 0.696551724 |
| 9 | 0.755058611 | 0.686135939 | 0.66597457 | 0.689062017 |
| 10 | 0.743625065 | 0.688552163 | 0.662017412 | 0.651557436 |
| 11 | 0.759450438 | 0.698991929 | 0.682823856 | 0.693271822 |
| 12 | 0.735761901 | 0.684351435 | 0.662903338 | 0.716205051 |
| 13 | 0.764308538 | 0.688365868 | 0.671201508 | 0.691913005 |
| 14 | 0.742427309 | 0.687941074 | 0.663677567 | 0.667412055 |
| 15 | 0.759926892 | 0.701122134 | 0.670611696 | 0.670547158 |
| 16 | 0.749812596 | 0.683057053 | 0.670317574 | 0.663958236 |
| 17 | 0.751778446 | 0.694411206 | 0.668605252 | 0.65960296 |
| 18 | 0.73779427 | 0.696804059 | 0.672905816 | 0.674316231 |
| 19 | 0.780292251 | 0.693520424 | 0.678583099 | 0.680140466 |
| 20 | 0.744647059 | 0.68963662 | 0.661011526 | 0.663031719 |
| 21 | 0.762488841 | 0.684539497 | 0.659039541 | 0.693504391 |
| 22 | 0.730439898 | 0.689945512 | 0.666843409 | 0.667748326 |
| 23 | 0.747883039 | 0.682585693 | 0.656260758 | 0.661972173 |
| 24 | 0.774781344 | 0.688007506 | 0.669507187 | 0.703363711 |
| 25 | 0.774762985 | 0.696329424 | 0.674630931 | 0.675382202 |
| 26 | 0.757738503 | 0.704276359 | 0.671510228 | 0.678455103 |
| 27 | 0.739228969 | 0.692938981 | 0.671965237 | 0.703981703 |
| 28 | 0.770088628 | 0.69465138 | 0.669569246 | 0.649848724 |
| 29 | 0.737934124 | 0.680084387 | 0.674153143 | 0.693643632 |
| 30 | 0.757804879 | 0.686389745 | 0.659978091 | 0.646482186 |
| 31 | 0.72733789 | 0.691439754 | 0.650510474 | 0.688607496 |
| 32 | 0.751041548 | 0.684487987 | 0.666833046 | 0.678051315 |
| 33 | 0.75800353 | 0.707515229 | 0.677532395 | 0.70292667 |
| 34 | 0.767821115 | 0.693465285 | 0.667299387 | 0.7065959 |
| 35 | 0.755137886 | 0.682919935 | 0.656637603 | 0.670076185 |
| 36 | 0.747185009 | 0.677408597 | 0.658044375 | 0.69698006 |
| 37 | 0.754126877 | 0.687465068 | 0.667823465 | 0.659202906 |
| 38 | 0.773798715 | 0.692376894 | 0.677166206 | 0.650985109 |
| 39 | 0.719145807 | 0.67556286 | 0.656088678 | 0.685139754 |
| 40 | 0.746572165 | 0.697022696 | 0.665747591 | 0.674920654 |
| 41 | 0.768641486 | 0.699911632 | 0.672225026 | 0.693569991 |
| 42 | 0.774001085 | 0.699065699 | 0.677559571 | 0.693220367 |
| 43 | 0.769617248 | 0.687866703 | 0.670857262 | 0.700821377 |
| 44 | 0.753956397 | 0.704359708 | 0.671666013 | 0.672757356 |
| 45 | 0.769418449 | 0.687385397 | 0.671401058 | 0.679656133 |
| 46 | 0.769635412 | 0.69813086 | 0.67101118 | 0.675167432 |
| 47 | 0.775668299 | 0.695757007 | 0.677023867 | 0.669491261 |
| 48 | 0.754747146 | 0.685021397 | 0.660669187 | 0.675361514 |
| 49 | 0.76080176 | 0.68742829 | 0.67960357 | 0.686468196 |
| 50 | 0.770194488 | 0.706445772 | 0.685701905 | 0.676668513 |
| 51 | 0.754619634 | 0.681782368 | 0.651263185 | 0.658954426 |
| 52 | 0.761509627 | 0.70072467 | 0.679014099 | 0.68631902 |
| 53 | 0.749469437 | 0.693625949 | 0.670287714 | 0.712398339 |
| 54 | 0.732689251 | 0.679070846 | 0.644762994 | 0.673919635 |
| 55 | 0.73622757 | 0.692790394 | 0.662004937 | 0.671008773 |
| 56 | 0.738156434 | 0.710551172 | 0.676930925 | 0.680523568 |
| 57 | 0.729965621 | 0.681044248 | 0.652711621 | 0.647261735 |
| 58 | 0.766837484 | 0.7089959 | 0.690197903 | 0.664680237 |
| 59 | 0.765665882 | 0.691866561 | 0.663802268 | 0.663958504 |
| 60 | 0.739355432 | 0.691129285 | 0.675067053 | 0.675632408 |
| 61 | 0.751005911 | 0.69628001 | 0.677716708 | 0.690039573 |
| 62 | 0.739489729 | 0.70152465 | 0.677124425 | 0.690820022 |
| 63 | 0.755053593 | 0.694709218 | 0.668945641 | 0.676122148 |
| 64 | 0.759956098 | 0.696466357 | 0.672490909 | 0.695432713 |
| 65 | 0.757082556 | 0.701646858 | 0.679168405 | 0.718805924 |
| 66 | 0.742551907 | 0.676519015 | 0.664994867 | 0.652741304 |
| 67 | 0.748790071 | 0.691771456 | 0.678625378 | 0.654908263 |
| 68 | 0.77425717 | 0.707631998 | 0.689506886 | 0.739813021 |
| 69 | 0.760112486 | 0.704007138 | 0.683077066 | 0.6684932 |
| 70 | 0.755884342 | 0.690359139 | 0.66096297 | 0.67807974 |
| 71 | 0.753987028 | 0.705278176 | 0.679844058 | 0.665239811 |
| 72 | 0.741592491 | 0.677117361 | 0.649452775 | 0.650709358 |
| 73 | 0.760238384 | 0.689616713 | 0.671571165 | 0.694306522 |
| 74 | 0.752736173 | 0.694013907 | 0.663280244 | 0.686533099 |
| 75 | 0.772214226 | 0.69053539 | 0.673284845 | 0.685442648 |
| 76 | 0.755039402 | 0.699083409 | 0.676566208 | 0.68972215 |
| 77 | 0.768792571 | 0.695691961 | 0.667211188 | 0.704260356 |
| 78 | 0.739869562 | 0.692442177 | 0.6698203 | 0.682678133 |
| 79 | 0.765125992 | 0.683052389 | 0.668496224 | 0.702754829 |
| 80 | 0.735450925 | 0.687351948 | 0.660755999 | 0.643852855 |
| 81 | 0.760397466 | 0.706645145 | 0.67579245 | 0.678703954 |
| 82 | 0.735697434 | 0.68991921 | 0.660412526 | 0.654041724 |
| 83 | 0.75253302 | 0.678814626 | 0.650233451 | 0.672990741 |
| 84 | 0.718592331 | 0.688365817 | 0.670968002 | 0.673358673 |
| 85 | 0.765947512 | 0.70382284 | 0.680132651 | 0.706128939 |
| 86 | 0.758865139 | 0.692054046 | 0.670848998 | 0.677888583 |
| 87 | 0.740426276 | 0.666059903 | 0.659176919 | 0.668407812 |
| 88 | 0.703537359 | 0.68012305 | 0.663404888 | 0.652564251 |
| 89 | 0.758385173 | 0.690205251 | 0.66584148 | 0.677969184 |
| 90 | 0.736095796 | 0.688335835 | 0.661209185 | 0.69842258 |
| 91 | 0.720713912 | 0.69861556 | 0.673052759 | 0.700191624 |
| 92 | 0.746162504 | 0.682877724 | 0.669344946 | 0.669005113 |
| 93 | 0.753514874 | 0.702909397 | 0.680158274 | 0.680534155 |
| 94 | 0.747202795 | 0.684570389 | 0.670436079 | 0.712708152 |
| 95 | 0.763100968 | 0.698767869 | 0.672411111 | 0.71769351 |
| 96 | 0.732917917 | 0.659270594 | 0.656193157 | 0.654952619 |
| 97 | 0.731041371 | 0.683751061 | 0.660560965 | 0.649166121 |
| 98 | 0.74572545 | 0.692445805 | 0.679388272 | 0.645785369 |
| 99 | 0.729072796 | 0.689480276 | 0.661545987 | 0.67519687 |
| 100 | 0.757301155 | 0.706718516 | 0.681358682 | 0.696697979 |
| 101 | 0.772707539 | 0.699279188 | 0.685415803 | 0.683840098 |
| 102 | 0.736571054 | 0.664700095 | 0.647833021 | 0.688552767 |
| 103 | 0.753056755 | 0.684403649 | 0.658708966 | 0.688847651 |
| 104 | 0.76136449 | 0.682520752 | 0.652562175 | 0.684710565 |
| 105 | 0.765169693 | 0.696657166 | 0.674866389 | 0.680805783 |
| 106 | 0.753966362 | 0.68803953 | 0.66215297 | 0.706240869 |
| 107 | 0.76332828 | 0.707721923 | 0.678728048 | 0.687906129 |
| 108 | 0.755843019 | 0.706097959 | 0.683811251 | 0.680867523 |
| 109 | 0.757935649 | 0.692662413 | 0.660601273 | 0.646404412 |
| 110 | 0.755905557 | 0.681863821 | 0.663100441 | 0.714004339 |
| 111 | 0.724762023 | 0.674749515 | 0.660134144 | 0.650262305 |
| 112 | 0.767151663 | 0.695376649 | 0.666102743 | 0.731217923 |
| 113 | 0.758940188 | 0.695472232 | 0.673422075 | 0.705270471 |
| 114 | 0.775887648 | 0.689208322 | 0.670258604 | 0.689069578 |
| 115 | 0.760305042 | 0.693613588 | 0.667047438 | 0.672518926 |
| 116 | 0.754956272 | 0.68479135 | 0.663260861 | 0.647389165 |
| 117 | 0.749833901 | 0.676219126 | 0.646264998 | 0.685943422 |
| 118 | 0.768955042 | 0.7041574 | 0.677440892 | 0.722943865 |
| 119 | 0.752332588 | 0.688330759 | 0.664380439 | 0.660809926 |
| 120 | 0.741389741 | 0.688086132 | 0.673547416 | 0.673457108 |
| 121 | 0.737404136 | 0.675939462 | 0.647482927 | 0.674914137 |
| 122 | 0.742612913 | 0.695198017 | 0.669813221 | 0.666009454 |
| 123 | 0.760532302 | 0.703941762 | 0.686101415 | 0.719340379 |
| 124 | 0.765385761 | 0.703329677 | 0.682678192 | 0.689225314 |
| 125 | 0.750960902 | 0.691451763 | 0.677073478 | 0.685302026 |
| 126 | 0.765977801 | 0.695910004 | 0.666209231 | 0.668196399 |
| 127 | 0.749297544 | 0.683637941 | 0.652629876 | 0.699078589 |
| 128 | 0.752302068 | 0.700147179 | 0.672869043 | 0.676032687 |
| 129 | 0.758513238 | 0.705541357 | 0.674769583 | 0.670840174 |
| 130 | 0.737664766 | 0.693577322 | 0.672331928 | 0.643790116 |
| 131 | 0.766539036 | 0.6882642 | 0.668807146 | 0.682709089 |
| 132 | 0.758425376 | 0.708122165 | 0.679821321 | 0.645832796 |
| 133 | 0.73569327 | 0.687195327 | 0.661930298 | 0.676153211 |
| 134 | 0.764313406 | 0.689172914 | 0.67336174 | 0.697708481 |
| 135 | 0.761038464 | 0.672794506 | 0.656855484 | 0.704358099 |
| 136 | 0.761432399 | 0.69340701 | 0.670288983 | 0.679760048 |
| 137 | 0.74140788 | 0.673954815 | 0.664558318 | 0.693699573 |
| 138 | 0.766890672 | 0.701485239 | 0.672280316 | 0.727500868 |
| 139 | 0.772792235 | 0.70589362 | 0.678520427 | 0.710550746 |
| 140 | 0.763020457 | 0.696507682 | 0.674064119 | 0.688615546 |
| 141 | 0.767966451 | 0.701253169 | 0.682297306 | 0.732148983 |
| 142 | 0.774007733 | 0.688606562 | 0.677520425 | 0.714295638 |
| 143 | 0.768236723 | 0.689256696 | 0.664696757 | 0.637927145 |
| 144 | 0.747438767 | 0.697158117 | 0.667112378 | 0.708195017 |
| 145 | 0.770043656 | 0.683926103 | 0.664743076 | 0.705090864 |
| 146 | 0.768445268 | 0.693474778 | 0.667287607 | 0.70028085 |
| 147 | 0.762618335 | 0.689493386 | 0.660312369 | 0.655790464 |
| 148 | 0.727348714 | 0.673093813 | 0.657697948 | 0.636446696 |
| 149 | 0.769490545 | 0.685045013 | 0.675582252 | 0.693915967 |
| 150 | 0.772610753 | 0.704625482 | 0.680324259 | 0.717918019 |
| 151 | 0.758370605 | 0.695900417 | 0.6701422 | 0.662313625 |
| 152 | 0.774771843 | 0.7053704 | 0.686986057 | 0.732131951 |
| 153 | 0.756296295 | 0.701056547 | 0.669458925 | 0.683906796 |
| 154 | 0.747471287 | 0.69173149 | 0.676585587 | 0.681080837 |
| 155 | 0.745136582 | 0.699076096 | 0.674175843 | 0.700669302 |
| 156 | 0.75702658 | 0.697603152 | 0.673198557 | 0.703121719 |
| 157 | 0.748076627 | 0.687639877 | 0.666279813 | 0.703608764 |
| 158 | 0.724040188 | 0.68531488 | 0.658433517 | 0.686472229 |
| 159 | 0.762161752 | 0.686704434 | 0.675045021 | 0.691907355 |
| 160 | 0.750958855 | 0.695505964 | 0.675241519 | 0.677688558 |
| 161 | 0.749088617 | 0.696506202 | 0.66436485 | 0.721308498 |
| 162 | 0.740226271 | 0.675610318 | 0.642723664 | 0.628596522 |
| 163 | 0.774871038 | 0.689120821 | 0.675796317 | 0.667250963 |
| 164 | 0.737414234 | 0.673663632 | 0.648046662 | 0.660897049 |
| 165 | 0.752468109 | 0.695750123 | 0.672102811 | 0.696733974 |
| 166 | 0.748660456 | 0.704549807 | 0.680415526 | 0.673369323 |
| 167 | 0.760720124 | 0.692708726 | 0.675189393 | 0.694564836 |
| 168 | 0.748246472 | 0.691885448 | 0.672109922 | 0.699779307 |
| 169 | 0.759357013 | 0.693320995 | 0.66315906 | 0.701553202 |
| 170 | 0.755964753 | 0.705803993 | 0.681319992 | 0.691943557 |
| 171 | 0.762764164 | 0.685903609 | 0.673716955 | 0.674947426 |
| 172 | 0.778773613 | 0.700295069 | 0.685255807 | 0.705345604 |
| 173 | 0.750042894 | 0.684208153 | 0.653181646 | 0.686262813 |
| 174 | 0.757865318 | 0.698540477 | 0.679282148 | 0.66998807 |
| 175 | 0.745429502 | 0.694086072 | 0.666821235 | 0.666143909 |
| 176 | 0.760160508 | 0.698443109 | 0.679447446 | 0.71151773 |
| 177 | 0.765659174 | 0.709936298 | 0.686458954 | 0.698395452 |
| 178 | 0.741407398 | 0.680139803 | 0.656157797 | 0.677270663 |
| 179 | 0.731497907 | 0.681444601 | 0.653166882 | 0.643330455 |
| 180 | 0.761648601 | 0.703331154 | 0.674261148 | 0.651530835 |
| 181 | 0.742611998 | 0.697570059 | 0.66710758 | 0.70722643 |
| 182 | 0.751758622 | 0.701429551 | 0.670941209 | 0.707633957 |
| 183 | 0.730069282 | 0.689954976 | 0.65187057 | 0.686993402 |
| 184 | 0.766821916 | 0.698673842 | 0.665833827 | 0.706086198 |
| 185 | 0.737907922 | 0.68455599 | 0.653266901 | 0.679200163 |
| 186 | 0.728302566 | 0.663055313 | 0.631119449 | 0.675640736 |
| 187 | 0.723453079 | 0.690891212 | 0.669370261 | 0.697591181 |
| 188 | 0.728926857 | 0.689448582 | 0.656978449 | 0.681535357 |
| 189 | 0.744482173 | 0.69681468 | 0.672774454 | 0.700740542 |
| 190 | 0.752515093 | 0.691184603 | 0.674407312 | 0.671879448 |
| 191 | 0.767997528 | 0.677164368 | 0.661124326 | 0.69428049 |
| 192 | 0.742615297 | 0.693020728 | 0.671592284 | 0.676344034 |
| 193 | 0.774513417 | 0.705387778 | 0.678358042 | 0.698290474 |
| 194 | 0.763458041 | 0.703013872 | 0.675871115 | 0.682773707 |
| 195 | 0.765841822 | 0.676193805 | 0.657240856 | 0.702708604 |
| 196 | 0.753119403 | 0.684263555 | 0.673122789 | 0.670709639 |
| 197 | 0.734170608 | 0.673463491 | 0.642683846 | 0.632480779 |
| 198 | 0.75364486 | 0.683079966 | 0.654615588 | 0.683849292 |
| 199 | 0.772034819 | 0.698964362 | 0.679936098 | 0.674165779 |
| 200 | 0.744837001 | 0.69660115 | 0.658557985 | 0.68032272 |
| 201 | 0.769410673 | 0.70683872 | 0.679840855 | 0.711232175 |
| 202 | 0.776091668 | 0.702367255 | 0.680238043 | 0.685443563 |
| 203 | 0.740660956 | 0.697636908 | 0.66302497 | 0.701927744 |
| 204 | 0.767448887 | 0.691489634 | 0.66901653 | 0.674627056 |
| 205 | 0.732069882 | 0.68586838 | 0.667388579 | 0.627002115 |
| 206 | 0.760736402 | 0.70229479 | 0.678353842 | 0.698278654 |
| 207 | 0.766375186 | 0.703249059 | 0.679683477 | 0.681292307 |
| 208 | 0.771533173 | 0.693327769 | 0.672017611 | 0.679977806 |
| 209 | 0.776340596 | 0.702072194 | 0.685533499 | 0.695384442 |
| 210 | 0.752718117 | 0.684535912 | 0.651843113 | 0.677624918 |
| 211 | 0.769044437 | 0.688438368 | 0.669291279 | 0.68278756 |
| 212 | 0.750736217 | 0.692317115 | 0.659915303 | 0.661055155 |
| 213 | 0.762759926 | 0.678347797 | 0.670413317 | 0.682875953 |
| 214 | 0.733033217 | 0.689999447 | 0.655847198 | 0.667841666 |
| 215 | 0.766426175 | 0.695061504 | 0.668323749 | 0.672125668 |
| 216 | 0.767535003 | 0.697029011 | 0.671945501 | 0.709152733 |
| 217 | 0.742906903 | 0.688137826 | 0.661998895 | 0.655099201 |
| 218 | 0.755391092 | 0.707690165 | 0.6853995 | 0.68881275 |
| 219 | 0.758415983 | 0.709983134 | 0.679062894 | 0.717949402 |
| 220 | 0.76790954 | 0.706364467 | 0.680267856 | 0.676016965 |
| 221 | 0.770103367 | 0.690095041 | 0.667744963 | 0.702907944 |
| 222 | 0.754713409 | 0.684162318 | 0.660792081 | 0.697337262 |
| 223 | 0.727901966 | 0.687591894 | 0.652631153 | 0.68008988 |
| 224 | 0.775360031 | 0.70797868 | 0.684559899 | 0.705728119 |
| 225 | 0.766369896 | 0.703375561 | 0.676193992 | 0.730585574 |
| 226 | 0.733977354 | 0.694001636 | 0.669891656 | 0.680861033 |
| 227 | 0.759991168 | 0.675985706 | 0.662197715 | 0.683000255 |
| 228 | 0.759306459 | 0.695626799 | 0.666603845 | 0.69105499 |
| 229 | 0.742258569 | 0.681751496 | 0.65451728 | 0.6751496 |
| 230 | 0.741585455 | 0.682660466 | 0.656142097 | 0.680686079 |
| 231 | 0.741451658 | 0.6899701 | 0.664403503 | 0.660264729 |
| 232 | 0.759869844 | 0.688995329 | 0.671689501 | 0.68533661 |
| 233 | 0.760972911 | 0.708718282 | 0.683231266 | 0.694911934 |
| 234 | 0.74076948 | 0.695092697 | 0.668265516 | 0.691977101 |
| 235 | 0.770167594 | 0.705870773 | 0.688666006 | 0.682173308 |
| 236 | 0.755944487 | 0.692106923 | 0.673200276 | 0.708030727 |
| 237 | 0.752554446 | 0.672667299 | 0.651620637 | 0.685343775 |
| 238 | 0.758214146 | 0.678392439 | 0.65940666 | 0.651746056 |
| 239 | 0.755622348 | 0.702730828 | 0.674700088 | 0.718252368 |
| 240 | 0.764479878 | 0.687248358 | 0.666545119 | 0.707980683 |
| 241 | 0.738332347 | 0.673571617 | 0.652451843 | 0.67486626 |
| 242 | 0.772875254 | 0.69128941 | 0.674186487 | 0.714406214 |
| 243 | 0.754414656 | 0.699124297 | 0.672887863 | 0.681089956 |
| 244 | 0.734657367 | 0.654088619 | 0.653960714 | 0.617876209 |
| 245 | 0.740052201 | 0.684250403 | 0.660252272 | 0.698041885 |
| 246 | 0.7664589 | 0.694174185 | 0.675212757 | 0.706070149 |
| 247 | 0.766037922 | 0.693102888 | 0.666006792 | 0.678847087 |
| 248 | 0.763869139 | 0.708983934 | 0.682710109 | 0.666610028 |
| 249 | 0.765279645 | 0.691444965 | 0.66559467 | 0.685022265 |
| 250 | 0.744881118 | 0.696736141 | 0.672542159 | 0.665817144 |
| 251 | 0.765072943 | 0.692250684 | 0.669978073 | 0.699867601 |
| 252 | 0.781899712 | 0.691285778 | 0.674971555 | 0.715278405 |
| 253 | 0.761803029 | 0.695893095 | 0.676985294 | 0.690434148 |
| 254 | 0.770612159 | 0.700045707 | 0.676656956 | 0.668115394 |
| 255 | 0.774358757 | 0.683760304 | 0.665168685 | 0.71533141 |
| 256 | 0.745879896 | 0.695670538 | 0.674616255 | 0.683848902 |
| 257 | 0.731226446 | 0.663342422 | 0.652261759 | 0.674524665 |
| 258 | 0.758158509 | 0.704256346 | 0.686089248 | 0.714248917 |
| 259 | 0.75364781 | 0.699511253 | 0.66365065 | 0.685183892 |
| 260 | 0.77125477 | 0.700008943 | 0.673986778 | 0.720769733 |
| 261 | 0.752464033 | 0.703415499 | 0.669664921 | 0.712302855 |
| 262 | 0.762769658 | 0.699513751 | 0.672788539 | 0.672448508 |
| 263 | 0.752349529 | 0.695670271 | 0.67024416 | 0.678633571 |
| 264 | 0.764485876 | 0.705173048 | 0.684388358 | 0.695493848 |
| 265 | 0.724562298 | 0.678829116 | 0.656889089 | 0.665666781 |
| 266 | 0.77273277 | 0.690918534 | 0.666494494 | 0.694997348 |
| 267 | 0.736616281 | 0.683699243 | 0.662614827 | 0.671386222 |
| 268 | 0.74191051 | 0.689268828 | 0.67341584 | 0.683211321 |
| 269 | 0.744723749 | 0.685954881 | 0.657345778 | 0.68985188 |
| 270 | 0.747027481 | 0.684529323 | 0.662126166 | 0.669730554 |
| 271 | 0.761528667 | 0.676676118 | 0.659740248 | 0.659841844 |
| 272 | 0.761486545 | 0.700494693 | 0.671535126 | 0.693924905 |
| 273 | 0.747112587 | 0.686489357 | 0.661816417 | 0.678753022 |
| 274 | 0.767772298 | 0.712201028 | 0.688037583 | 0.718566852 |
| 275 | 0.776403352 | 0.692875528 | 0.680827114 | 0.702574519 |
| 276 | 0.762503297 | 0.693660104 | 0.662092888 | 0.671528819 |
| 277 | 0.758064927 | 0.694630647 | 0.672676331 | 0.685846708 |
| 278 | 0.764514949 | 0.687196738 | 0.657531419 | 0.701274414 |
| 279 | 0.740799997 | 0.69805813 | 0.669042767 | 0.673352216 |
| 280 | 0.760974412 | 0.700144084 | 0.676368834 | 0.680158073 |
| 281 | 0.770997236 | 0.693136954 | 0.67695406 | 0.710217165 |
| 282 | 0.753558576 | 0.700170549 | 0.674311775 | 0.689796654 |
| 283 | 0.763421756 | 0.698233149 | 0.669212615 | 0.666716648 |
| 284 | 0.778201477 | 0.6878118 | 0.674893001 | 0.708648151 |
| 285 | 0.756000623 | 0.696452957 | 0.664795258 | 0.70027674 |
| 286 | 0.780960542 | 0.703946025 | 0.685235729 | 0.719205377 |
| 287 | 0.729741301 | 0.684190855 | 0.666824882 | 0.666274759 |
| 288 | 0.749096808 | 0.699279794 | 0.68212357 | 0.6571156 |
| 289 | 0.745725947 | 0.678311987 | 0.664433216 | 0.673332969 |
| 290 | 0.728860023 | 0.679416468 | 0.643411113 | 0.699542299 |
| 291 | 0.741943832 | 0.707374303 | 0.687333963 | 0.689781118 |
| 292 | 0.766025631 | 0.697536625 | 0.67375293 | 0.692007813 |
| 293 | 0.777060936 | 0.702806234 | 0.684947082 | 0.710320052 |
| 294 | 0.768550318 | 0.693331538 | 0.666153536 | 0.729386227 |
| 295 | 0.729073275 | 0.676758551 | 0.657226256 | 0.657668471 |
| 296 | 0.755080986 | 0.696018326 | 0.659372526 | 0.657933948 |
| 297 | 0.764871411 | 0.69250155 | 0.675098051 | 0.670698702 |
| 298 | 0.771004508 | 0.69189941 | 0.667469593 | 0.680447613 |
| 299 | 0.741473574 | 0.687741753 | 0.660564024 | 0.681189745 |
| 300 | 0.756200976 | 0.676945445 | 0.663416425 | 0.682399067 |
| 301 | 0.749398257 | 0.679166568 | 0.644831217 | 0.687107942 |
| 302 | 0.764293698 | 0.693105086 | 0.666062191 | 0.658285296 |
| 303 | 0.769577163 | 0.697349572 | 0.679723006 | 0.669371371 |
| 304 | 0.751278697 | 0.696315264 | 0.667455793 | 0.684768863 |
| 305 | 0.718614 | 0.679038695 | 0.657109981 | 0.686172158 |
| 306 | 0.760333109 | 0.693644365 | 0.676238689 | 0.688665504 |
| 307 | 0.755631156 | 0.703605561 | 0.676490115 | 0.703162075 |
| 308 | 0.76897508 | 0.702816228 | 0.686368673 | 0.709103104 |
| 309 | 0.748706735 | 0.694739817 | 0.659774709 | 0.688021522 |
| 310 | 0.709597511 | 0.687298024 | 0.653774037 | 0.68665842 |
| 311 | 0.733789283 | 0.680344539 | 0.656767953 | 0.694572255 |
| 312 | 0.760786149 | 0.674792613 | 0.652680623 | 0.678380518 |
| 313 | 0.723532402 | 0.676180581 | 0.667543848 | 0.685794173 |
| 314 | 0.751457179 | 0.693537126 | 0.659887917 | 0.680114783 |
| 315 | 0.745222187 | 0.677089001 | 0.662104905 | 0.684077388 |
| 316 | 0.760962048 | 0.694449215 | 0.683953657 | 0.699955191 |
| 317 | 0.775860076 | 0.694053904 | 0.674298527 | 0.704234962 |
| 318 | 0.742442623 | 0.688314855 | 0.662289453 | 0.686797157 |
| 319 | 0.75440717 | 0.677704219 | 0.666984817 | 0.700681182 |
| 320 | 0.774109567 | 0.702115845 | 0.682125755 | 0.69935411 |
| 321 | 0.750059067 | 0.675247844 | 0.648193547 | 0.70325492 |
| 322 | 0.714802389 | 0.672944443 | 0.653262931 | 0.673627217 |
| 323 | 0.761994889 | 0.694956655 | 0.673071862 | 0.699800484 |
| 324 | 0.747146176 | 0.703606816 | 0.667706398 | 0.693239859 |
| 325 | 0.777976182 | 0.701760572 | 0.680758888 | 0.687421023 |
| 326 | 0.761369222 | 0.697308725 | 0.665879819 | 0.715811777 |
| 327 | 0.746545591 | 0.683781152 | 0.648097819 | 0.684394951 |
| 328 | 0.750121754 | 0.690619026 | 0.669797462 | 0.677267723 |
| 329 | 0.748474428 | 0.695016011 | 0.663577546 | 0.683479956 |
| 330 | 0.757941104 | 0.703811085 | 0.679082403 | 0.65437466 |
| 331 | 0.742628476 | 0.690705119 | 0.664734325 | 0.708510717 |
| 332 | 0.763576392 | 0.705312714 | 0.683163188 | 0.663732467 |
| 333 | 0.777068758 | 0.698236987 | 0.681957884 | 0.700392423 |
| 334 | 0.760816872 | 0.688944963 | 0.672287732 | 0.669069952 |
| 335 | 0.741633761 | 0.679442588 | 0.667544674 | 0.682325349 |
| 336 | 0.738559529 | 0.689478863 | 0.677372473 | 0.700898664 |
| 337 | 0.775140955 | 0.709393536 | 0.687474139 | 0.665610274 |
| 338 | 0.762599271 | 0.697345873 | 0.678762332 | 0.655928898 |
| 339 | 0.759218971 | 0.69490847 | 0.671286089 | 0.702397295 |
| 340 | 0.74887467 | 0.66698088 | 0.656320394 | 0.659519497 |
| 341 | 0.763441512 | 0.710480376 | 0.681902677 | 0.680480845 |
| 342 | 0.763148029 | 0.705249631 | 0.683055686 | 0.704406534 |
| 343 | 0.763670214 | 0.69494277 | 0.667007274 | 0.659977592 |
| 344 | 0.748255211 | 0.683716778 | 0.672406633 | 0.681688599 |
| 345 | 0.77082071 | 0.693257416 | 0.672684951 | 0.686819037 |
| 346 | 0.760862849 | 0.682121555 | 0.656446634 | 0.672761665 |
| 347 | 0.750083036 | 0.670799498 | 0.642626378 | 0.657951619 |
| 348 | 0.758160724 | 0.683831288 | 0.65517475 | 0.71996613 |
| 349 | 0.735625316 | 0.701508901 | 0.67530379 | 0.700145886 |
| 350 | 0.751885984 | 0.698865847 | 0.674340832 | 0.721117359 |
| 351 | 0.754042291 | 0.692024235 | 0.659392241 | 0.697001857 |
| 352 | 0.752164971 | 0.692202785 | 0.675510305 | 0.674765894 |
| 353 | 0.732486323 | 0.674005302 | 0.643510975 | 0.660761246 |
| 354 | 0.753654262 | 0.690735526 | 0.660384349 | 0.681958759 |
| 355 | 0.74502598 | 0.683109967 | 0.672618847 | 0.690014249 |
| 356 | 0.764298172 | 0.676421025 | 0.658689019 | 0.680527737 |
| 357 | 0.763810705 | 0.707702576 | 0.679226179 | 0.702660151 |
| 358 | 0.760353656 | 0.686321442 | 0.673557697 | 0.702642471 |
| 359 | 0.773423442 | 0.684426555 | 0.670356436 | 0.693509426 |
| 360 | 0.760509492 | 0.700738428 | 0.682860259 | 0.693552286 |
| 361 | 0.757494129 | 0.66864147 | 0.660027179 | 0.679769346 |
| 362 | 0.75870354 | 0.695284215 | 0.673540253 | 0.672970522 |
| 363 | 0.747622833 | 0.693102362 | 0.67691105 | 0.661043172 |
| 364 | 0.760229085 | 0.705279551 | 0.674013407 | 0.704143913 |
| 365 | 0.775179799 | 0.704547028 | 0.680791031 | 0.673151444 |
| 366 | 0.747756161 | 0.67850808 | 0.650788698 | 0.695166728 |
| 367 | 0.731195195 | 0.689174914 | 0.667771136 | 0.661075865 |
| 368 | 0.758855888 | 0.694415832 | 0.665012169 | 0.668741424 |
| 369 | 0.769211859 | 0.694374793 | 0.675978291 | 0.707702712 |
| 370 | 0.762703303 | 0.701035394 | 0.681872647 | 0.70458918 |
| 371 | 0.726386234 | 0.697394497 | 0.666792149 | 0.654630189 |
| 372 | 0.762209989 | 0.691206333 | 0.678271633 | 0.678245529 |
| 373 | 0.747540732 | 0.690961981 | 0.673968824 | 0.674164594 |
| 374 | 0.75225424 | 0.692393119 | 0.667625117 | 0.72252949 |
| 375 | 0.730837902 | 0.69562054 | 0.669448048 | 0.69349267 |
| 376 | 0.766092998 | 0.696650411 | 0.676130171 | 0.659484855 |
| 377 | 0.740779771 | 0.692712861 | 0.65976033 | 0.708156762 |
| 378 | 0.766413543 | 0.7020409 | 0.677854782 | 0.66623584 |
| 379 | 0.751347672 | 0.687084793 | 0.677799914 | 0.679686668 |
| 380 | 0.73955109 | 0.691980602 | 0.659601568 | 0.634153557 |
| 381 | 0.764712176 | 0.685686168 | 0.661948125 | 0.676890316 |
| 382 | 0.760466925 | 0.697064627 | 0.66861104 | 0.667186753 |
| 383 | 0.761432019 | 0.70650716 | 0.682400513 | 0.698265118 |
| 384 | 0.758355498 | 0.695638575 | 0.676263033 | 0.674470671 |
| 385 | 0.777531448 | 0.701344736 | 0.679637654 | 0.713985332 |
| 386 | 0.772143046 | 0.693147664 | 0.679186293 | 0.68738803 |
| 387 | 0.758531248 | 0.694025605 | 0.662929982 | 0.699101982 |
| 388 | 0.71185841 | 0.682471238 | 0.656968937 | 0.670301983 |
| 389 | 0.741131517 | 0.678733825 | 0.663510086 | 0.678440415 |
| 390 | 0.744454867 | 0.68573169 | 0.676159743 | 0.684130081 |
| 391 | 0.77707883 | 0.70060957 | 0.680437765 | 0.722507968 |
| 392 | 0.741423347 | 0.693755998 | 0.65543253 | 0.660663867 |
| 393 | 0.762221185 | 0.70227016 | 0.674024414 | 0.679710326 |
| 394 | 0.766854348 | 0.686608396 | 0.672466946 | 0.688602996 |
| 395 | 0.760405317 | 0.692084577 | 0.67822542 | 0.702333506 |
| 396 | 0.756249222 | 0.690660082 | 0.663796469 | 0.686195372 |
| 397 | 0.771142253 | 0.703736245 | 0.680060317 | 0.718936287 |
| 398 | 0.748057735 | 0.673149547 | 0.645770381 | 0.661674632 |
| 399 | 0.745469518 | 0.691600513 | 0.668905816 | 0.674417443 |
| 400 | 0.734701645 | 0.680062972 | 0.647811238 | 0.685642685 |
| 401 | 0.775156227 | 0.703043111 | 0.680610938 | 0.714681029 |
| 402 | 0.761637575 | 0.704556151 | 0.68310696 | 0.711996786 |
| 403 | 0.758389818 | 0.687839401 | 0.669984298 | 0.687541896 |
| 404 | 0.755381708 | 0.697974728 | 0.680118169 | 0.670128162 |
| 405 | 0.767889457 | 0.697729905 | 0.672710226 | 0.682410843 |
| 406 | 0.7356068 | 0.687370785 | 0.664548968 | 0.645309441 |
| 407 | 0.693604409 | 0.672716015 | 0.632839448 | 0.688174138 |
| 408 | 0.768342403 | 0.679992897 | 0.662987081 | 0.661207923 |
| 409 | 0.761990689 | 0.69498178 | 0.664148695 | 0.691993961 |
| 410 | 0.743001763 | 0.690812097 | 0.65873847 | 0.671750892 |
| 411 | 0.765725372 | 0.701111551 | 0.679922708 | 0.716698884 |
| 412 | 0.767368452 | 0.680986554 | 0.660434512 | 0.683131669 |
| 413 | 0.728517436 | 0.683935377 | 0.650067166 | 0.64274836 |
| 414 | 0.769287002 | 0.695550834 | 0.671096102 | 0.694774894 |
| 415 | 0.774271182 | 0.692299411 | 0.666324782 | 0.670864042 |
| 416 | 0.771058611 | 0.69821771 | 0.679975136 | 0.643229793 |
| 417 | 0.762665949 | 0.694188337 | 0.675176377 | 0.689874668 |
| 418 | 0.74394196 | 0.688212353 | 0.672995321 | 0.681558446 |
| 419 | 0.750646137 | 0.702525356 | 0.670561871 | 0.713545489 |
| 420 | 0.753479876 | 0.67705752 | 0.661047939 | 0.698875433 |
| 421 | 0.766670323 | 0.684580058 | 0.660208223 | 0.672200724 |
| 422 | 0.758777392 | 0.686548134 | 0.668639029 | 0.657513271 |
| 423 | 0.748076747 | 0.682521797 | 0.64911991 | 0.673511242 |
| 424 | 0.753746662 | 0.696274301 | 0.66827165 | 0.687478108 |
| 425 | 0.742122119 | 0.690941914 | 0.661099079 | 0.685668063 |
| 426 | 0.761682355 | 0.704232351 | 0.677214414 | 0.677634197 |
| 427 | 0.732667651 | 0.693684544 | 0.66963936 | 0.65905926 |
| 428 | 0.763326906 | 0.697854932 | 0.679662398 | 0.704298584 |
| 429 | 0.752689048 | 0.681156077 | 0.663133321 | 0.711177775 |
| 430 | 0.751444417 | 0.690599978 | 0.680380493 | 0.661646254 |
| 431 | 0.76825676 | 0.696779211 | 0.677801194 | 0.712126839 |
| 432 | 0.765818443 | 0.700938092 | 0.678014592 | 0.691058215 |
| 433 | 0.763945789 | 0.696279032 | 0.680721832 | 0.68803823 |
| 434 | 0.736849844 | 0.686971418 | 0.666787401 | 0.672290832 |
| 435 | 0.771377818 | 0.702407362 | 0.684853658 | 0.664365194 |
| 436 | 0.760931484 | 0.687464619 | 0.672208603 | 0.690675372 |
| 437 | 0.76901451 | 0.703461148 | 0.67598506 | 0.714204898 |
| 438 | 0.744233548 | 0.675398474 | 0.641025932 | 0.656172452 |
| 439 | 0.780878402 | 0.697815385 | 0.68154772 | 0.707329373 |
| 440 | 0.775929212 | 0.707231573 | 0.68282187 | 0.67304419 |
| 441 | 0.752826167 | 0.687415277 | 0.669981911 | 0.678150029 |
| 442 | 0.76920551 | 0.688360897 | 0.667152482 | 0.684730125 |
| 443 | 0.774790392 | 0.70133539 | 0.674985736 | 0.695084865 |
| 444 | 0.766871191 | 0.692286692 | 0.669093072 | 0.681113075 |
| 445 | 0.778176357 | 0.690372392 | 0.671373558 | 0.714147105 |
| 446 | 0.758152061 | 0.686975678 | 0.663690843 | 0.689626919 |
| 447 | 0.772728818 | 0.696808855 | 0.677825699 | 0.672573968 |
| 448 | 0.742455572 | 0.685025415 | 0.665171664 | 0.659925053 |
| 449 | 0.744256397 | 0.696799915 | 0.66554015 | 0.678513719 |
| 450 | 0.778044141 | 0.700935496 | 0.681696842 | 0.703164178 |
| 451 | 0.766431877 | 0.701996974 | 0.680534241 | 0.707088984 |
| 452 | 0.779805144 | 0.704547201 | 0.688353039 | 0.687289059 |
| 453 | 0.737179061 | 0.671483686 | 0.655046333 | 0.669842499 |
| 454 | 0.738129729 | 0.694747886 | 0.661762299 | 0.697273345 |
| 455 | 0.767904884 | 0.704133743 | 0.684899482 | 0.686620651 |
| 456 | 0.747357987 | 0.685663206 | 0.671092611 | 0.671717436 |
| 457 | 0.749505921 | 0.705049017 | 0.680457608 | 0.703785514 |
| 458 | 0.761911101 | 0.674978131 | 0.660063845 | 0.68280601 |
| 459 | 0.738705944 | 0.68538925 | 0.658159297 | 0.67735464 |
| 460 | 0.756831623 | 0.691356949 | 0.671817067 | 0.679172613 |
| 461 | 0.758362488 | 0.701696775 | 0.676143308 | 0.715747254 |
| 462 | 0.775678441 | 0.69939627 | 0.679164435 | 0.719387549 |
| 463 | 0.758590231 | 0.693043435 | 0.674311332 | 0.679344414 |
| 464 | 0.749587541 | 0.689212864 | 0.669333256 | 0.704712521 |
| 465 | 0.760200058 | 0.693113175 | 0.667380246 | 0.661100239 |
| 466 | 0.766673611 | 0.693179993 | 0.675917897 | 0.677138194 |
| 467 | 0.738822306 | 0.7042408 | 0.674610951 | 0.65457037 |
| 468 | 0.772923352 | 0.697011122 | 0.679849819 | 0.716503424 |
| 469 | 0.745495768 | 0.687070497 | 0.665622814 | 0.665409231 |
| 470 | 0.775809768 | 0.695475854 | 0.674906213 | 0.724123085 |
| 471 | 0.754755416 | 0.687683021 | 0.676338441 | 0.682291819 |
| 472 | 0.743011333 | 0.694880765 | 0.67954678 | 0.683517385 |
| 473 | 0.752790678 | 0.692076836 | 0.675617783 | 0.669551143 |
| 474 | 0.715964933 | 0.68711705 | 0.65290575 | 0.665267942 |
| 475 | 0.765385316 | 0.701558415 | 0.674813444 | 0.667045236 |
| 476 | 0.772411712 | 0.695040716 | 0.675162778 | 0.706800579 |
| 477 | 0.762416888 | 0.70424832 | 0.681973826 | 0.685518335 |
| 478 | 0.758686692 | 0.696625904 | 0.670412117 | 0.702489435 |
| 479 | 0.735564466 | 0.681814284 | 0.665364088 | 0.657498666 |
| 480 | 0.710441754 | 0.673249882 | 0.657228225 | 0.660648185 |
| 481 | 0.759547253 | 0.696906936 | 0.681084925 | 0.688289483 |
| 482 | 0.73362741 | 0.691220641 | 0.667790778 | 0.661124505 |
| 483 | 0.764235862 | 0.70361209 | 0.674480391 | 0.690634386 |
| 484 | 0.727243192 | 0.699881338 | 0.673386417 | 0.691743943 |
| 485 | 0.763031219 | 0.704575432 | 0.68113712 | 0.712717481 |
| 486 | 0.742602994 | 0.692665295 | 0.677789094 | 0.687502388 |
| 487 | 0.776427565 | 0.69248821 | 0.680367271 | 0.719512492 |
| 488 | 0.775764663 | 0.688803538 | 0.677463356 | 0.64069882 |
| 489 | 0.775150849 | 0.70174751 | 0.681962995 | 0.682027064 |
| 490 | 0.737011741 | 0.663859329 | 0.65240966 | 0.654981096 |
| 491 | 0.726346263 | 0.674431782 | 0.660618359 | 0.631025702 |
| 492 | 0.757862954 | 0.701642278 | 0.68606424 | 0.645968855 |
| 493 | 0.746312916 | 0.690143309 | 0.673532236 | 0.690912766 |
| 494 | 0.752006643 | 0.683963122 | 0.666385771 | 0.658324778 |
| 495 | 0.744894136 | 0.680534524 | 0.670969429 | 0.642751617 |
| 496 | 0.752991545 | 0.699103907 | 0.684640491 | 0.681163844 |
| 497 | 0.764807946 | 0.67960564 | 0.65384581 | 0.679707317 |
| 498 | 0.756395367 | 0.700082311 | 0.669814412 | 0.685212219 |
| 499 | 0.750927353 | 0.669061749 | 0.638458078 | 0.696533676 |
| 500 | 0.764796924 | 0.696585836 | 0.677351697 | 0.679361283 |
| 501 | 0.752415983 | 0.699210616 | 0.6756211 | 0.689868184 |
| 502 | 0.772522978 | 0.69374711 | 0.670032796 | 0.672012918 |
| 503 | 0.751575476 | 0.698697165 | 0.676704756 | 0.729275362 |
| 504 | 0.727744317 | 0.692987571 | 0.665371141 | 0.667956199 |
| 505 | 0.76291931 | 0.700397019 | 0.673069079 | 0.68556701 |
| 506 | 0.749348 | 0.700006026 | 0.663546036 | 0.66066022 |
| 507 | 0.766517475 | 0.704223957 | 0.67667061 | 0.689982499 |
| 508 | 0.740810648 | 0.690341818 | 0.664539256 | 0.65383767 |
| 509 | 0.781659368 | 0.70156539 | 0.686444665 | 0.703532118 |
| 510 | 0.756844522 | 0.665530078 | 0.663950864 | 0.7173826 |
| 511 | 0.767970589 | 0.697552903 | 0.681175404 | 0.696778871 |
| 512 | 0.738580963 | 0.685881505 | 0.6745856 | 0.689424553 |
| 513 | 0.774451907 | 0.695392542 | 0.677776121 | 0.689286376 |
| 514 | 0.765330534 | 0.698663183 | 0.676231218 | 0.699167775 |
| 515 | 0.766991304 | 0.707787838 | 0.684877173 | 0.708527434 |
| 516 | 0.760134233 | 0.704700079 | 0.672806527 | 0.687938221 |
| 517 | 0.733560287 | 0.684195613 | 0.665518394 | 0.711355516 |
| 518 | 0.767262151 | 0.702979497 | 0.676927789 | 0.68580337 |
| 519 | 0.75071805 | 0.675611921 | 0.645052166 | 0.675705612 |
| 520 | 0.750467597 | 0.699880461 | 0.669987108 | 0.672699447 |
| 521 | 0.729615646 | 0.691444452 | 0.655282915 | 0.676130123 |
| 522 | 0.751868687 | 0.693876188 | 0.667939317 | 0.707285739 |
| 523 | 0.764957293 | 0.708743058 | 0.681345286 | 0.690262121 |
| 524 | 0.765809276 | 0.690818466 | 0.667322913 | 0.661928723 |
| 525 | 0.737500617 | 0.676863042 | 0.665958749 | 0.625724557 |
| 526 | 0.744056322 | 0.685021375 | 0.665805014 | 0.710517508 |
| 527 | 0.760036681 | 0.705163899 | 0.674401478 | 0.677173386 |
| 528 | 0.748925591 | 0.669811012 | 0.654603575 | 0.662588177 |
| 529 | 0.752636601 | 0.699534952 | 0.671997038 | 0.682260182 |
| 530 | 0.75956223 | 0.697138223 | 0.677155071 | 0.684893702 |
| 531 | 0.741126293 | 0.684789972 | 0.65523974 | 0.682984136 |
| 532 | 0.72699555 | 0.680230356 | 0.648320945 | 0.669482725 |
| 533 | 0.751453221 | 0.675393941 | 0.652410874 | 0.715076262 |
| 534 | 0.728777029 | 0.690073748 | 0.657160885 | 0.672385221 |
| 535 | 0.728296534 | 0.685431514 | 0.657602635 | 0.720725053 |
| 536 | 0.753002891 | 0.693039162 | 0.660641113 | 0.715319957 |
| 537 | 0.769199911 | 0.683931012 | 0.661211051 | 0.684235051 |
| 538 | 0.766995904 | 0.69566967 | 0.673635876 | 0.736815872 |
| 539 | 0.764094394 | 0.70040634 | 0.672051941 | 0.726005552 |
| 540 | 0.740091289 | 0.684365388 | 0.666227245 | 0.679331969 |
| 541 | 0.760856066 | 0.696266376 | 0.670478791 | 0.681487477 |
| 542 | 0.761272687 | 0.693263981 | 0.668491619 | 0.70394763 |
| 543 | 0.738616733 | 0.706650853 | 0.675301187 | 0.702425042 |
| 544 | 0.701529798 | 0.660574896 | 0.656412966 | 0.630370281 |
| 545 | 0.77144051 | 0.681720835 | 0.665840101 | 0.680912161 |
| 546 | 0.764601974 | 0.693559865 | 0.672174785 | 0.694598098 |
| 547 | 0.752279323 | 0.693512922 | 0.672171869 | 0.654122455 |
| 548 | 0.74945792 | 0.699538722 | 0.672114835 | 0.683892932 |
| 549 | 0.76458387 | 0.69158199 | 0.663118801 | 0.646031191 |
| 550 | 0.775648221 | 0.691378619 | 0.672743904 | 0.709350492 |
| 551 | 0.763607931 | 0.69048923 | 0.679118784 | 0.68893391 |
| 552 | 0.770461322 | 0.707338008 | 0.681771649 | 0.689474636 |
| 553 | 0.769845504 | 0.701128848 | 0.671095436 | 0.723839599 |
| 554 | 0.767517206 | 0.696052238 | 0.682076941 | 0.707399949 |
| 555 | 0.780230049 | 0.696206745 | 0.681956502 | 0.683278181 |
| 556 | 0.743461859 | 0.679942965 | 0.660321267 | 0.711948929 |
| 557 | 0.77003171 | 0.707337715 | 0.686412978 | 0.701793805 |
| 558 | 0.768427098 | 0.712331085 | 0.689106922 | 0.705255409 |
| 559 | 0.779226658 | 0.707042511 | 0.685691861 | 0.718594211 |
| 560 | 0.777994183 | 0.696070856 | 0.677800808 | 0.663594732 |
| 561 | 0.733505642 | 0.683800704 | 0.668392913 | 0.689539948 |
| 562 | 0.767162055 | 0.687174722 | 0.670528932 | 0.662348547 |
| 563 | 0.751863181 | 0.691224997 | 0.660074429 | 0.6805197 |
| 564 | 0.745028263 | 0.691320385 | 0.671022738 | 0.695624676 |
| 565 | 0.717910862 | 0.672937752 | 0.65236144 | 0.676623147 |
| 566 | 0.77832464 | 0.692693817 | 0.671198252 | 0.68917383 |
| 567 | 0.772819193 | 0.698333969 | 0.678950686 | 0.66861891 |
| 568 | 0.772754257 | 0.691897465 | 0.675487385 | 0.690911511 |
| 569 | 0.748698249 | 0.699156664 | 0.677469123 | 0.671038858 |
| 570 | 0.74100371 | 0.703354045 | 0.668820727 | 0.663789517 |
| 571 | 0.712940479 | 0.672680767 | 0.657631006 | 0.676493745 |
| 572 | 0.7577489 | 0.705121316 | 0.680044195 | 0.698404578 |
| 573 | 0.776013896 | 0.701625651 | 0.680742091 | 0.71263307 |
| 574 | 0.702097246 | 0.677116251 | 0.661828275 | 0.642259178 |
| 575 | 0.739826522 | 0.690354415 | 0.667053775 | 0.690471551 |
| 576 | 0.765840895 | 0.706861746 | 0.679508367 | 0.685336602 |
| 577 | 0.712049937 | 0.666100897 | 0.657407724 | 0.678969942 |
| 578 | 0.736672651 | 0.692422373 | 0.665418883 | 0.657895147 |
| 579 | 0.750275845 | 0.686943839 | 0.665335745 | 0.698425963 |
| 580 | 0.769719497 | 0.681587326 | 0.669048997 | 0.661670557 |
| 581 | 0.754413314 | 0.686472006 | 0.673604438 | 0.71855078 |
| 582 | 0.778347114 | 0.701845465 | 0.677754597 | 0.674272145 |
| 583 | 0.768170339 | 0.688068676 | 0.662891168 | 0.686208802 |
| 584 | 0.762869537 | 0.695291702 | 0.674023 | 0.663363631 |
| 585 | 0.770658157 | 0.692065921 | 0.666058592 | 0.691427508 |
| 586 | 0.751076938 | 0.672779278 | 0.651342122 | 0.689158815 |
| 587 | 0.763447602 | 0.692038466 | 0.676583735 | 0.672464865 |
| 588 | 0.77006128 | 0.704210715 | 0.681657995 | 0.704867052 |
| 589 | 0.76208957 | 0.691807357 | 0.666585228 | 0.6849639 |
| 590 | 0.754376005 | 0.694380177 | 0.672034077 | 0.686256116 |
| 591 | 0.767243982 | 0.70098104 | 0.67138367 | 0.704188702 |
| 592 | 0.727756572 | 0.686449707 | 0.667431929 | 0.67242281 |
| 593 | 0.761497734 | 0.704721048 | 0.675001011 | 0.67337929 |
| 594 | 0.761925482 | 0.69297177 | 0.677059535 | 0.680178278 |
| 595 | 0.741747114 | 0.699650471 | 0.665103309 | 0.692805992 |
| 596 | 0.7505396 | 0.706956202 | 0.673773068 | 0.677038211 |
| 597 | 0.762038929 | 0.707058624 | 0.685623651 | 0.722246031 |
| 598 | 0.762410672 | 0.690060211 | 0.671988786 | 0.700009475 |
| 599 | 0.760132186 | 0.692507619 | 0.6679353 | 0.672238036 |
| 600 | 0.772415845 | 0.693164316 | 0.669551913 | 0.684528719 |
| 601 | 0.770425976 | 0.685993035 | 0.665529269 | 0.700080143 |
| 602 | 0.753809582 | 0.686233953 | 0.675742186 | 0.691803584 |
| 603 | 0.763310743 | 0.705360821 | 0.681169596 | 0.68573436 |
| 604 | 0.768709224 | 0.699386313 | 0.678011945 | 0.664821014 |
| 605 | 0.764775264 | 0.697237481 | 0.66828047 | 0.699182126 |
| 606 | 0.765828009 | 0.703111837 | 0.678107592 | 0.667668497 |
| 607 | 0.745970223 | 0.664715896 | 0.6537651 | 0.67434418 |
| 608 | 0.756717518 | 0.692571773 | 0.673280296 | 0.710599756 |
| 609 | 0.760136827 | 0.692370861 | 0.679443514 | 0.697223256 |
| 610 | 0.751494014 | 0.693509424 | 0.669754138 | 0.690364262 |
| 611 | 0.758403392 | 0.690259686 | 0.668207717 | 0.702104923 |
| 612 | 0.734271674 | 0.698026939 | 0.663207411 | 0.649681474 |
| 613 | 0.767194204 | 0.695490567 | 0.671761626 | 0.704246701 |
| 614 | 0.761998288 | 0.697782174 | 0.664534159 | 0.68025132 |
| 615 | 0.737977928 | 0.694998597 | 0.669736828 | 0.670557316 |
| 616 | 0.757269301 | 0.694872525 | 0.660739947 | 0.718830431 |
| 617 | 0.723341995 | 0.679274005 | 0.65756829 | 0.676125037 |
| 618 | 0.775252979 | 0.694925787 | 0.674289888 | 0.688939728 |
| 619 | 0.731122073 | 0.671204174 | 0.644623469 | 0.680356016 |
| 620 | 0.716937923 | 0.677584943 | 0.642984547 | 0.677392356 |
| 621 | 0.726272153 | 0.690294858 | 0.667531218 | 0.6526634 |
| 622 | 0.742991021 | 0.687903376 | 0.66042578 | 0.672981837 |
| 623 | 0.78159638 | 0.705857921 | 0.68619116 | 0.726076581 |
| 624 | 0.77022543 | 0.701079125 | 0.68344376 | 0.68173546 |
| 625 | 0.740346021 | 0.689026934 | 0.654629255 | 0.661615841 |
| 626 | 0.722396653 | 0.684275699 | 0.650097909 | 0.672876475 |
| 627 | 0.762426824 | 0.682344702 | 0.666822708 | 0.679772496 |
| 628 | 0.7660635 | 0.704460694 | 0.682757871 | 0.664540213 |
| 629 | 0.766269101 | 0.702255399 | 0.681155843 | 0.702143624 |
| 630 | 0.760775457 | 0.701859101 | 0.678065963 | 0.702586357 |
| 631 | 0.718710472 | 0.671551472 | 0.642058686 | 0.67729843 |
| 632 | 0.75778161 | 0.682623983 | 0.668336539 | 0.684117822 |
| 633 | 0.765253953 | 0.707980148 | 0.677638545 | 0.674336459 |
| 634 | 0.76339628 | 0.6999364 | 0.667084958 | 0.701589396 |
| 635 | 0.744772089 | 0.688953669 | 0.668477093 | 0.66113485 |
| 636 | 0.765773226 | 0.693270545 | 0.665133887 | 0.679529899 |
| 637 | 0.715016016 | 0.673229849 | 0.654190812 | 0.661328577 |
| 638 | 0.736859507 | 0.670665427 | 0.665201008 | 0.696869007 |
| 639 | 0.760493883 | 0.699449109 | 0.681514207 | 0.697121425 |
| 640 | 0.756764382 | 0.699472099 | 0.668737669 | 0.694051369 |
| 641 | 0.769464933 | 0.69885903 | 0.669317628 | 0.691032234 |
| 642 | 0.718699857 | 0.685092892 | 0.656650007 | 0.690443117 |
| 643 | 0.776137188 | 0.694548929 | 0.678978947 | 0.685150171 |
| 644 | 0.745808546 | 0.664270826 | 0.657219545 | 0.707540068 |
| 645 | 0.759195725 | 0.702995833 | 0.67320309 | 0.69814633 |
| 646 | 0.759585969 | 0.690159553 | 0.6674732 | 0.679371995 |
| 647 | 0.772461383 | 0.695366009 | 0.68018576 | 0.684489184 |
| 648 | 0.758509767 | 0.707203504 | 0.684626026 | 0.69590464 |
| 649 | 0.763857003 | 0.686732785 | 0.664805309 | 0.676211174 |
| 650 | 0.740215939 | 0.693176954 | 0.663260004 | 0.664065396 |
| 651 | 0.745483348 | 0.67520602 | 0.653586295 | 0.689508597 |
| 652 | 0.780868722 | 0.69992222 | 0.681309965 | 0.685638069 |
| 653 | 0.749085612 | 0.685330574 | 0.655094886 | 0.679505081 |
| 654 | 0.749497616 | 0.69117554 | 0.657935449 | 0.697135189 |
| 655 | 0.759833657 | 0.695842013 | 0.678777077 | 0.680275629 |
| 656 | 0.731685104 | 0.697370743 | 0.664387612 | 0.708086513 |
| 657 | 0.752653737 | 0.685695681 | 0.653178516 | 0.671286472 |
| 658 | 0.743261214 | 0.681745629 | 0.649307252 | 0.706880918 |
| 659 | 0.779772815 | 0.700965044 | 0.682864371 | 0.699161075 |
| 660 | 0.767466812 | 0.696440516 | 0.674194888 | 0.681873075 |
| 661 | 0.732891325 | 0.687045043 | 0.653424267 | 0.698918259 |
| 662 | 0.752675699 | 0.703533302 | 0.672912556 | 0.687806775 |
| 663 | 0.763030091 | 0.704200708 | 0.673032457 | 0.6882391 |
| 664 | 0.745495399 | 0.678932417 | 0.647769878 | 0.676663831 |
| 665 | 0.728887972 | 0.677688685 | 0.652479174 | 0.691170872 |
| 666 | 0.763572106 | 0.682555814 | 0.655198535 | 0.713729117 |
| 667 | 0.75932145 | 0.692560707 | 0.660518731 | 0.680169827 |
| 668 | 0.765598653 | 0.701025122 | 0.684742415 | 0.689277955 |
| 669 | 0.7755159 | 0.707746175 | 0.681834062 | 0.67678397 |
| 670 | 0.73077766 | 0.681810797 | 0.668541686 | 0.685826671 |
| 671 | 0.755363405 | 0.700756655 | 0.675930677 | 0.681098018 |
| 672 | 0.774383681 | 0.696343206 | 0.673579747 | 0.699265513 |
| 673 | 0.765007459 | 0.699034345 | 0.683013125 | 0.68386774 |
| 674 | 0.759952673 | 0.70452396 | 0.676684283 | 0.658744069 |
| 675 | 0.75448577 | 0.695599737 | 0.677608374 | 0.701778113 |
| 676 | 0.771088219 | 0.698257081 | 0.678026153 | 0.726816959 |
| 677 | 0.763688194 | 0.710729901 | 0.691701622 | 0.684316286 |
| 678 | 0.759349107 | 0.696120228 | 0.66565295 | 0.675624009 |
| 679 | 0.773435418 | 0.69479776 | 0.681602061 | 0.665035493 |
| 680 | 0.77020551 | 0.692404147 | 0.671109106 | 0.695228076 |
| 681 | 0.766476963 | 0.702496457 | 0.678491689 | 0.697741204 |
| 682 | 0.725989286 | 0.680782303 | 0.658394729 | 0.67872414 |
| 683 | 0.766770011 | 0.697902063 | 0.668558585 | 0.699420607 |
| 684 | 0.748920777 | 0.685297019 | 0.671875839 | 0.674038229 |
| 685 | 0.732759654 | 0.687849298 | 0.65414496 | 0.689104808 |
| 686 | 0.767636362 | 0.705198497 | 0.68275245 | 0.682806799 |
| 687 | 0.771760347 | 0.703552717 | 0.67825915 | 0.682034246 |
| 688 | 0.726457344 | 0.690104518 | 0.669554938 | 0.679872997 |
| 689 | 0.75000161 | 0.703530758 | 0.685112705 | 0.676772963 |
| 690 | 0.746725994 | 0.699601409 | 0.675141325 | 0.681085482 |
| 691 | 0.774399895 | 0.703889516 | 0.680978495 | 0.674198283 |
| 692 | 0.762599713 | 0.681811422 | 0.657767075 | 0.676132264 |
| 693 | 0.754638013 | 0.694422978 | 0.677807412 | 0.672558054 |
| 694 | 0.729103018 | 0.677480272 | 0.657942795 | 0.6543363 |
| 695 | 0.777531629 | 0.703520077 | 0.680422843 | 0.689306192 |
| 696 | 0.771638227 | 0.702612007 | 0.680113826 | 0.728009552 |
| 697 | 0.773990359 | 0.706446563 | 0.684597528 | 0.683349252 |
| 698 | 0.733772316 | 0.691661638 | 0.670729354 | 0.670707817 |
| 699 | 0.758184684 | 0.695994243 | 0.674503513 | 0.683068521 |
| 700 | 0.765423239 | 0.706378738 | 0.675128676 | 0.661080221 |
| 701 | 0.764828797 | 0.692440559 | 0.670047893 | 0.701166635 |
| 702 | 0.751340148 | 0.695467969 | 0.678885114 | 0.650408104 |
| 703 | 0.770061944 | 0.707616542 | 0.678441645 | 0.693878513 |
| 704 | 0.757814853 | 0.700649031 | 0.67295736 | 0.652241591 |
| 705 | 0.723732555 | 0.68278114 | 0.665042511 | 0.677797139 |
| 706 | 0.728273747 | 0.669928053 | 0.668583975 | 0.68988998 |
| 707 | 0.774038942 | 0.702960609 | 0.687867698 | 0.675294589 |
| 708 | 0.765088574 | 0.707219829 | 0.683317497 | 0.689125601 |
| 709 | 0.735924117 | 0.686962462 | 0.655742091 | 0.660825447 |
| 710 | 0.756116497 | 0.694204397 | 0.679332356 | 0.688860048 |
| 711 | 0.728657248 | 0.678764835 | 0.666885897 | 0.671616835 |
| 712 | 0.775078765 | 0.706675336 | 0.687262043 | 0.661865932 |
| 713 | 0.743364632 | 0.692543677 | 0.672221214 | 0.640579435 |
| 714 | 0.757489462 | 0.707296086 | 0.680899206 | 0.724601371 |
| 715 | 0.78097459 | 0.70369807 | 0.686797567 | 0.735251394 |
| 716 | 0.763275058 | 0.68998044 | 0.663582112 | 0.68138535 |
| 717 | 0.777355365 | 0.706524422 | 0.687342289 | 0.713521563 |
| 718 | 0.764666777 | 0.703625144 | 0.680701494 | 0.686910511 |
| 719 | 0.746630671 | 0.682341146 | 0.668601111 | 0.668375921 |
| 720 | 0.763679104 | 0.708070544 | 0.680540761 | 0.702375256 |
| 721 | 0.764826514 | 0.705185449 | 0.681519081 | 0.697915934 |
| 722 | 0.774790029 | 0.689946116 | 0.671288291 | 0.698580317 |
| 723 | 0.738721705 | 0.673377743 | 0.652290056 | 0.676745424 |
| 724 | 0.775912781 | 0.693934732 | 0.67241225 | 0.670318113 |
| 725 | 0.761158143 | 0.689893384 | 0.66934579 | 0.676848254 |
| 726 | 0.751802092 | 0.694186694 | 0.673905269 | 0.677813047 |
| 727 | 0.739299034 | 0.676623658 | 0.668169872 | 0.682458464 |
| 728 | 0.76633427 | 0.689758999 | 0.661827386 | 0.673118628 |
| 729 | 0.723240888 | 0.665651511 | 0.650668599 | 0.656328832 |
| 730 | 0.756666503 | 0.696063667 | 0.675481315 | 0.69896241 |
| 731 | 0.766627728 | 0.705907339 | 0.67574979 | 0.68187726 |
| 732 | 0.752152617 | 0.692300059 | 0.673546282 | 0.68586084 |
| 733 | 0.736630561 | 0.685471929 | 0.661160606 | 0.653950337 |
| 734 | 0.728559493 | 0.704225638 | 0.677593354 | 0.677990524 |
| 735 | 0.733088551 | 0.691361126 | 0.669251935 | 0.683148428 |
| 736 | 0.757357417 | 0.688038382 | 0.673158229 | 0.692761678 |
| 737 | 0.740932274 | 0.700157332 | 0.67289995 | 0.677700114 |
| 738 | 0.774657724 | 0.684119506 | 0.670394615 | 0.727340061 |
| 739 | 0.76247527 | 0.703370842 | 0.67337476 | 0.691946014 |
| 740 | 0.766525791 | 0.689985903 | 0.666357829 | 0.722812559 |
| 741 | 0.756846952 | 0.693539706 | 0.664901561 | 0.679809659 |
| 742 | 0.733067218 | 0.688912451 | 0.671520205 | 0.685860875 |
| 743 | 0.766844778 | 0.698053916 | 0.681456179 | 0.697083454 |
| 744 | 0.774798879 | 0.689540398 | 0.666445262 | 0.675447013 |
| 745 | 0.770784935 | 0.696292234 | 0.675425884 | 0.725299312 |
| 746 | 0.773800285 | 0.694065266 | 0.679937257 | 0.703382072 |
| 747 | 0.73963727 | 0.691678899 | 0.661643865 | 0.701147103 |
| 748 | 0.770628627 | 0.679721686 | 0.659806987 | 0.678044533 |
| 749 | 0.754769849 | 0.699596521 | 0.683542542 | 0.68370879 |
| 750 | 0.759325077 | 0.696048617 | 0.676063897 | 0.696280045 |
| 751 | 0.759316428 | 0.686524879 | 0.660387218 | 0.703865846 |
| 752 | 0.764402194 | 0.703543111 | 0.67977824 | 0.69885918 |
| 753 | 0.76883827 | 0.694984264 | 0.672013235 | 0.681207635 |
| 754 | 0.754850984 | 0.693119043 | 0.668836475 | 0.710117656 |
| 755 | 0.748455087 | 0.676537119 | 0.662740961 | 0.694508803 |
| 756 | 0.755880658 | 0.682002279 | 0.664091151 | 0.696439011 |
| 757 | 0.755217749 | 0.692439421 | 0.674467792 | 0.689579963 |
| 758 | 0.767462788 | 0.703496728 | 0.678516096 | 0.69933266 |
| 759 | 0.768781114 | 0.704841002 | 0.686791459 | 0.702367565 |
| 760 | 0.734417258 | 0.690079641 | 0.656094677 | 0.676262591 |
| 761 | 0.716857597 | 0.671719133 | 0.652168217 | 0.688616722 |
| 762 | 0.752033542 | 0.691119653 | 0.670410503 | 0.670370187 |
| 763 | 0.732714591 | 0.694278864 | 0.671870611 | 0.656069062 |
| 764 | 0.734977775 | 0.677825473 | 0.656241296 | 0.640765588 |
| 765 | 0.767177235 | 0.70442261 | 0.682277808 | 0.712322042 |
| 766 | 0.757742919 | 0.703003724 | 0.670707849 | 0.70189306 |
| 767 | 0.764096862 | 0.695341229 | 0.680121814 | 0.67366586 |
| 768 | 0.725446613 | 0.691570223 | 0.655870538 | 0.696845325 |
| 769 | 0.752452923 | 0.688524548 | 0.664853935 | 0.699638848 |
| 770 | 0.778106068 | 0.693103114 | 0.672748844 | 0.717246373 |
| 771 | 0.76112807 | 0.695361737 | 0.668544262 | 0.695241163 |
| 772 | 0.759032363 | 0.691103628 | 0.66029008 | 0.632186544 |
| 773 | 0.779151887 | 0.689744282 | 0.67223404 | 0.720430293 |
| 774 | 0.758732813 | 0.706309412 | 0.679568448 | 0.712587755 |
| 775 | 0.757339108 | 0.693668758 | 0.662803949 | 0.695735039 |
| 776 | 0.754827136 | 0.689818008 | 0.673148583 | 0.679720171 |
| 777 | 0.734047878 | 0.682938173 | 0.649983564 | 0.658484343 |
| 778 | 0.767564717 | 0.704642835 | 0.674877595 | 0.724684321 |
| 779 | 0.773495021 | 0.703587578 | 0.687780956 | 0.712088944 |
| 780 | 0.722390655 | 0.686139986 | 0.665409701 | 0.695855467 |
| 781 | 0.776991209 | 0.697056824 | 0.677327478 | 0.724924777 |
| 782 | 0.761330648 | 0.69386694 | 0.666760631 | 0.696439715 |
| 783 | 0.731876344 | 0.697698796 | 0.670590942 | 0.661177817 |
| 784 | 0.763877664 | 0.694705122 | 0.676547467 | 0.704503881 |
| 785 | 0.769586784 | 0.705183596 | 0.678950129 | 0.6712897 |
| 786 | 0.720987542 | 0.665236667 | 0.659582163 | 0.669510504 |
| 787 | 0.713673742 | 0.681940049 | 0.666965096 | 0.706985397 |
| 788 | 0.737867962 | 0.695235787 | 0.661604558 | 0.6975203 |
| 789 | 0.765600895 | 0.698832019 | 0.678613314 | 0.710278396 |
| 790 | 0.758513888 | 0.684598821 | 0.657498724 | 0.703713299 |
| 791 | 0.727351973 | 0.684137358 | 0.654525552 | 0.653580492 |
| 792 | 0.753879844 | 0.678153343 | 0.666346559 | 0.637537966 |
| 793 | 0.747914052 | 0.698497922 | 0.675027973 | 0.630182438 |
| 794 | 0.764712071 | 0.708102042 | 0.677845149 | 0.717505335 |
| 795 | 0.760377789 | 0.690780472 | 0.661567515 | 0.663414137 |
| 796 | 0.75402342 | 0.702981347 | 0.671338714 | 0.677076657 |
| 797 | 0.753467289 | 0.683300491 | 0.655469943 | 0.675044302 |
| 798 | 0.765134834 | 0.679940982 | 0.657303404 | 0.676384279 |
| 799 | 0.772056765 | 0.700193136 | 0.679636219 | 0.707161259 |
| 800 | 0.743884216 | 0.689659378 | 0.662059714 | 0.712902901 |
| 801 | 0.770275228 | 0.697129066 | 0.670965879 | 0.709479001 |
| 802 | 0.774554243 | 0.6963535 | 0.672928247 | 0.713425796 |
| 803 | 0.763440276 | 0.706046887 | 0.677959363 | 0.683417892 |
| 804 | 0.729690947 | 0.686571086 | 0.652402186 | 0.661320845 |
| 805 | 0.772304691 | 0.699033518 | 0.672713632 | 0.694787898 |
| 806 | 0.756648102 | 0.685667965 | 0.670119012 | 0.713810461 |
| 807 | 0.753903869 | 0.702586434 | 0.678063744 | 0.739588458 |
| 808 | 0.754202548 | 0.685018829 | 0.677880193 | 0.720862346 |
| 809 | 0.750231788 | 0.698565818 | 0.674909645 | 0.714454148 |
| 810 | 0.766979625 | 0.68624925 | 0.654974149 | 0.67406944 |
| 811 | 0.766599936 | 0.709434703 | 0.68663778 | 0.684997392 |
| 812 | 0.735430354 | 0.667572594 | 0.664925359 | 0.672935552 |
| 813 | 0.750273315 | 0.692098507 | 0.680889391 | 0.680220609 |
| 814 | 0.760875901 | 0.694479033 | 0.680767296 | 0.683491845 |
| 815 | 0.764523065 | 0.706268199 | 0.686057181 | 0.674354691 |
| 816 | 0.759864539 | 0.709832278 | 0.67638544 | 0.691318172 |
| 817 | 0.764313049 | 0.694840811 | 0.67091052 | 0.690894526 |
| 818 | 0.770231663 | 0.691376315 | 0.682065324 | 0.703144293 |
| 819 | 0.74721049 | 0.688727538 | 0.667232543 | 0.673743557 |
| 820 | 0.765631977 | 0.698350056 | 0.679730388 | 0.670479061 |
| 821 | 0.741800045 | 0.691011167 | 0.676083004 | 0.66355076 |
| 822 | 0.718610423 | 0.695876195 | 0.660732851 | 0.680961777 |
| 823 | 0.739172802 | 0.693670015 | 0.666631317 | 0.692952027 |
| 824 | 0.751331523 | 0.68954989 | 0.661322993 | 0.708658744 |
| 825 | 0.764283398 | 0.697583055 | 0.675829016 | 0.690508318 |
| 826 | 0.73326843 | 0.688289601 | 0.664795158 | 0.691179153 |
| 827 | 0.773217345 | 0.697498236 | 0.674881897 | 0.686428289 |
| 828 | 0.735299433 | 0.706266076 | 0.675933896 | 0.67005706 |
| 829 | 0.747968166 | 0.692177538 | 0.674278286 | 0.689989474 |
| 830 | 0.749460752 | 0.703523404 | 0.677886797 | 0.690501654 |
| 831 | 0.776272744 | 0.707868841 | 0.685901639 | 0.694901111 |
| 832 | 0.735981466 | 0.678902989 | 0.666399077 | 0.699113465 |
| 833 | 0.755841315 | 0.695558494 | 0.677031725 | 0.687052458 |
| 834 | 0.751984766 | 0.694786868 | 0.671653248 | 0.671109277 |
| 835 | 0.755670555 | 0.692471174 | 0.666313929 | 0.676976739 |
| 836 | 0.747999972 | 0.669100374 | 0.6504915 | 0.687122498 |
| 837 | 0.726541528 | 0.682134664 | 0.655249195 | 0.70250649 |
| 838 | 0.756848704 | 0.690849706 | 0.669515559 | 0.69925792 |
| 839 | 0.769414667 | 0.709673925 | 0.680083427 | 0.671743041 |
| 840 | 0.765872457 | 0.706929049 | 0.686909568 | 0.683175201 |
| 841 | 0.757328049 | 0.711043683 | 0.688735767 | 0.696408354 |
| 842 | 0.76428761 | 0.705688822 | 0.678962754 | 0.686361956 |
| 843 | 0.752422484 | 0.701046961 | 0.66997297 | 0.661424529 |
| 844 | 0.731862353 | 0.68428867 | 0.655732502 | 0.666699502 |
| 845 | 0.754837905 | 0.678099094 | 0.658514336 | 0.721587498 |
| 846 | 0.744374088 | 0.697046909 | 0.66015823 | 0.688928516 |
| 847 | 0.748737144 | 0.690478679 | 0.658646062 | 0.660155339 |
| 848 | 0.759009288 | 0.700044107 | 0.673281157 | 0.718447729 |
| 849 | 0.758683914 | 0.684782391 | 0.67113974 | 0.687710445 |
| 850 | 0.762345194 | 0.689474553 | 0.680856296 | 0.687896937 |
| 851 | 0.766623002 | 0.703301394 | 0.678256203 | 0.704393794 |
| 852 | 0.773134385 | 0.689952843 | 0.66930334 | 0.685306858 |
| 853 | 0.73724175 | 0.697869264 | 0.666466096 | 0.700319645 |
| 854 | 0.769308002 | 0.692361569 | 0.667636315 | 0.70230918 |
| 855 | 0.710576556 | 0.671356922 | 0.651511114 | 0.684984342 |
| 856 | 0.751306629 | 0.680229036 | 0.662464018 | 0.682597553 |
| 857 | 0.770103893 | 0.696145506 | 0.675311605 | 0.683500236 |
| 858 | 0.746030799 | 0.679326361 | 0.65924603 | 0.657638578 |
| 859 | 0.747668376 | 0.693074471 | 0.663833764 | 0.694407423 |
| 860 | 0.744836142 | 0.700745856 | 0.666438517 | 0.675799865 |
| 861 | 0.754320543 | 0.699958036 | 0.66754249 | 0.689898776 |
| 862 | 0.760011995 | 0.690450904 | 0.667340851 | 0.689440778 |
| 863 | 0.740627151 | 0.691905649 | 0.671760667 | 0.676167652 |
| 864 | 0.736482979 | 0.680526754 | 0.660605963 | 0.689738511 |
| 865 | 0.778677753 | 0.696815059 | 0.681712557 | 0.697312345 |
| 866 | 0.748308749 | 0.685724306 | 0.678242546 | 0.672472919 |
| 867 | 0.770024743 | 0.703987102 | 0.676818933 | 0.683290261 |
| 868 | 0.742229355 | 0.686921118 | 0.664191294 | 0.651185999 |
| 869 | 0.745085389 | 0.688631629 | 0.671414316 | 0.674703847 |
| 870 | 0.766802036 | 0.697428718 | 0.670686265 | 0.681663556 |
| 871 | 0.75765944 | 0.693895408 | 0.681353751 | 0.680462721 |
| 872 | 0.734886773 | 0.691487408 | 0.677952402 | 0.663736065 |
| 873 | 0.753163808 | 0.685965528 | 0.656473826 | 0.667592519 |
| 874 | 0.761591973 | 0.701621186 | 0.670602062 | 0.673635516 |
| 875 | 0.765232614 | 0.682640155 | 0.65997635 | 0.680071872 |
| 876 | 0.767321333 | 0.705844803 | 0.68730476 | 0.676386311 |
| 877 | 0.763911035 | 0.693215699 | 0.662571736 | 0.658144873 |
| 878 | 0.750992179 | 0.69755424 | 0.670860036 | 0.673262148 |
| 879 | 0.765343517 | 0.706621193 | 0.676777148 | 0.691495718 |
| 880 | 0.762597646 | 0.695960384 | 0.673602814 | 0.699737182 |
| 881 | 0.751531635 | 0.696897406 | 0.668641061 | 0.666725153 |
| 882 | 0.745171958 | 0.687162474 | 0.659403319 | 0.64664026 |
| 883 | 0.738339778 | 0.696210298 | 0.661418603 | 0.658886162 |
| 884 | 0.773090212 | 0.693041296 | 0.675872566 | 0.68082377 |
| 885 | 0.760129851 | 0.69050807 | 0.673655726 | 0.713550006 |
| 886 | 0.765245067 | 0.691433573 | 0.662459821 | 0.68887273 |
| 887 | 0.771404028 | 0.707572446 | 0.685952818 | 0.697556162 |
| 888 | 0.764894947 | 0.701324008 | 0.680409955 | 0.662198656 |
| 889 | 0.716393109 | 0.671495114 | 0.667784717 | 0.664134591 |
| 890 | 0.761858617 | 0.692512623 | 0.681687467 | 0.649278601 |
| 891 | 0.735687599 | 0.689001428 | 0.663315802 | 0.678906707 |
| 892 | 0.736128648 | 0.692314903 | 0.679009297 | 0.666987411 |
| 893 | 0.772448801 | 0.707626451 | 0.683917 | 0.700993641 |
| 894 | 0.746765979 | 0.7045036 | 0.666436259 | 0.681728323 |
| 895 | 0.759184955 | 0.687901547 | 0.672109488 | 0.663920413 |
| 896 | 0.741453423 | 0.688990792 | 0.674922802 | 0.696978774 |
| 897 | 0.758710283 | 0.689964 | 0.672542304 | 0.685908395 |
| 898 | 0.74997426 | 0.691307716 | 0.667504333 | 0.680278266 |
| 899 | 0.755038144 | 0.689960268 | 0.671682015 | 0.665901705 |
| 900 | 0.737418107 | 0.694701022 | 0.6599594 | 0.675482539 |
| 901 | 0.738758842 | 0.702163218 | 0.67479249 | 0.681545041 |
| 902 | 0.773456657 | 0.699994212 | 0.675459085 | 0.709863398 |
| 903 | 0.760310846 | 0.699400895 | 0.676824881 | 0.678255132 |
| 904 | 0.76070812 | 0.702018443 | 0.686411988 | 0.683565312 |
| 905 | 0.753352427 | 0.705357356 | 0.676193278 | 0.70117885 |
| 906 | 0.75788519 | 0.687874126 | 0.660200044 | 0.648743113 |
| 907 | 0.754574589 | 0.693132238 | 0.674981025 | 0.665471444 |
| 908 | 0.766580503 | 0.681021144 | 0.660567806 | 0.666752262 |
| 909 | 0.768110073 | 0.682447479 | 0.675128612 | 0.670981522 |
| 910 | 0.769868099 | 0.694407824 | 0.67035217 | 0.704787076 |
| 911 | 0.764667626 | 0.702395009 | 0.68159987 | 0.703770154 |
| 912 | 0.767948876 | 0.699667672 | 0.681014476 | 0.708979341 |
| 913 | 0.752205793 | 0.696875036 | 0.667165326 | 0.687150646 |
| 914 | 0.74238972 | 0.691341869 | 0.660587376 | 0.69503416 |
| 915 | 0.730264605 | 0.685257588 | 0.655474406 | 0.692457504 |
| 916 | 0.747144815 | 0.681483967 | 0.653675592 | 0.657625945 |
| 917 | 0.76590178 | 0.696281982 | 0.674325607 | 0.700579524 |
| 918 | 0.764777224 | 0.698327911 | 0.676686742 | 0.699315272 |
| 919 | 0.754897345 | 0.689680181 | 0.664588345 | 0.655807732 |
| 920 | 0.751634307 | 0.702681007 | 0.681109202 | 0.71418859 |
| 921 | 0.749104276 | 0.679135841 | 0.670260373 | 0.695030607 |
| 922 | 0.750201628 | 0.688644326 | 0.668868762 | 0.676330121 |
| 923 | 0.759199968 | 0.696413851 | 0.680928463 | 0.656559604 |
| 924 | 0.752444051 | 0.697451306 | 0.677457147 | 0.65474643 |
| 925 | 0.755462278 | 0.697966473 | 0.680173125 | 0.690113565 |
| 926 | 0.774103593 | 0.693177636 | 0.67481366 | 0.670847832 |
| 927 | 0.763120625 | 0.6879953 | 0.669108956 | 0.703668204 |
| 928 | 0.727520111 | 0.680240684 | 0.661795195 | 0.694735002 |
| 929 | 0.748394739 | 0.677188059 | 0.665642946 | 0.666702951 |
| 930 | 0.756941327 | 0.69667891 | 0.676321492 | 0.66921009 |
| 931 | 0.738965104 | 0.696770028 | 0.663504109 | 0.700193845 |
| 932 | 0.762249005 | 0.688739075 | 0.664284239 | 0.696132728 |
| 933 | 0.753996719 | 0.696704018 | 0.674211956 | 0.69534183 |
| 934 | 0.763288411 | 0.69336317 | 0.663750257 | 0.662696449 |
| 935 | 0.709065026 | 0.66247808 | 0.64632133 | 0.653801791 |
| 936 | 0.750281615 | 0.695136931 | 0.664428791 | 0.685948587 |
| 937 | 0.750127821 | 0.685967605 | 0.654472228 | 0.655190888 |
| 938 | 0.752341497 | 0.693902663 | 0.675559268 | 0.66525063 |
| 939 | 0.755421643 | 0.677462517 | 0.670175485 | 0.703948334 |
| 940 | 0.756704442 | 0.693603458 | 0.664310496 | 0.688719849 |
| 941 | 0.745490647 | 0.679843635 | 0.665737479 | 0.679886517 |
| 942 | 0.744884027 | 0.680718643 | 0.643901792 | 0.685106308 |
| 943 | 0.750021409 | 0.69778628 | 0.675718916 | 0.643685567 |
| 944 | 0.756740815 | 0.68992511 | 0.658445937 | 0.678038748 |
| 945 | 0.745324144 | 0.690465945 | 0.656416655 | 0.712565213 |
| 946 | 0.759004507 | 0.707157381 | 0.682073199 | 0.690194217 |
| 947 | 0.771247756 | 0.699232481 | 0.672529786 | 0.644982822 |
| 948 | 0.758991886 | 0.671091093 | 0.645257964 | 0.657170313 |
| 949 | 0.719151335 | 0.681656926 | 0.659680422 | 0.658202005 |
| 950 | 0.746243276 | 0.687618385 | 0.666484059 | 0.677511206 |
| 951 | 0.74324323 | 0.699809752 | 0.667664477 | 0.678101244 |
| 952 | 0.774164593 | 0.702044664 | 0.67972139 | 0.687230616 |
| 953 | 0.77125049 | 0.694183365 | 0.670795658 | 0.700033156 |
| 954 | 0.755969666 | 0.70117844 | 0.671978463 | 0.694577619 |
| 955 | 0.713480018 | 0.669869367 | 0.656856421 | 0.666521511 |
| 956 | 0.776130298 | 0.706068251 | 0.684809164 | 0.685957133 |
| 957 | 0.767366859 | 0.692857412 | 0.669225241 | 0.687184376 |
| 958 | 0.766430965 | 0.700091974 | 0.683283011 | 0.666787342 |
| 959 | 0.737155907 | 0.704961838 | 0.668280588 | 0.652160648 |
| 960 | 0.751165662 | 0.703620927 | 0.675632706 | 0.702122745 |
| 961 | 0.772788092 | 0.699963255 | 0.677494616 | 0.696654449 |
| 962 | 0.765279353 | 0.681685417 | 0.665498596 | 0.723183828 |
| 963 | 0.743827914 | 0.698207499 | 0.671818114 | 0.651729287 |
| 964 | 0.755242422 | 0.700290148 | 0.675834273 | 0.673285073 |
| 965 | 0.750449979 | 0.675388902 | 0.664090818 | 0.683922422 |
| 966 | 0.759960602 | 0.685699958 | 0.669453917 | 0.667796749 |
| 967 | 0.732451667 | 0.666741885 | 0.644272069 | 0.648519267 |
| 968 | 0.741562411 | 0.701575433 | 0.679380136 | 0.698425229 |
| 969 | 0.755541922 | 0.69681865 | 0.670107367 | 0.702929021 |
| 970 | 0.761627237 | 0.695061472 | 0.663291705 | 0.686502699 |
| 971 | 0.756689858 | 0.69807041 | 0.677525341 | 0.702758348 |
| 972 | 0.749593642 | 0.68914794 | 0.659737868 | 0.692714067 |
| 973 | 0.76702194 | 0.701780256 | 0.682185353 | 0.650804042 |
| 974 | 0.776690818 | 0.702975215 | 0.685462614 | 0.68322423 |
| 975 | 0.769472374 | 0.71003595 | 0.688022333 | 0.667767193 |
| 976 | 0.775837091 | 0.698271509 | 0.684161087 | 0.682125505 |
| 977 | 0.772376184 | 0.699370469 | 0.678884849 | 0.697416424 |
| 978 | 0.743814071 | 0.703033424 | 0.675498857 | 0.719375813 |
| 979 | 0.765862861 | 0.69138377 | 0.666923737 | 0.662030192 |
| 980 | 0.748345488 | 0.695557526 | 0.669604656 | 0.686559809 |
| 981 | 0.754933262 | 0.68304729 | 0.654012243 | 0.64412168 |
| 982 | 0.76768396 | 0.698187545 | 0.680799899 | 0.698653849 |
| 983 | 0.752566663 | 0.701286711 | 0.669598774 | 0.66076908 |
| 984 | 0.769966216 | 0.696262294 | 0.667910095 | 0.70184765 |
| 985 | 0.727325404 | 0.681993429 | 0.648049035 | 0.678941642 |
| 986 | 0.767016601 | 0.694925026 | 0.669437952 | 0.676922512 |
| 987 | 0.759366961 | 0.707906012 | 0.68100494 | 0.703261095 |
| 988 | 0.763032347 | 0.691169825 | 0.670091697 | 0.70101104 |
| 989 | 0.762466361 | 0.704004814 | 0.680687615 | 0.651088736 |
| 990 | 0.762764652 | 0.695347528 | 0.680947799 | 0.66777693 |
| 991 | 0.745326706 | 0.697673774 | 0.66926884 | 0.685228638 |
| 992 | 0.758024689 | 0.712639026 | 0.687498859 | 0.696392841 |
| 993 | 0.733018997 | 0.683262805 | 0.656779942 | 0.692270763 |
| 994 | 0.685643314 | 0.657322892 | 0.639677537 | 0.639308082 |
| 995 | 0.737768677 | 0.677111898 | 0.660600328 | 0.71385273 |
| 996 | 0.731764195 | 0.686401734 | 0.660349695 | 0.676451109 |
| 997 | 0.752417478 | 0.695594017 | 0.683242916 | 0.679342194 |
| 998 | 0.764211752 | 0.704876789 | 0.684622213 | 0.696314968 |
| 999 | 0.736614872 | 0.668297404 | 0.648637664 | 0.66134944 |
| 1000 | 0.750955594 | 0.695793858 | 0.671375439 | 0.703246547 |

**Supplementary Table 5**. bootstrap 1000 times’ results.
